# Supplementary material for: The sharp structural switch of covalent cages mediated by subtle variation of directing groups
Source: Nat Commun. 2023 Aug 2;14:4627. doi: 10.1038/s41467-023-40255-4 (PMC10397198; doi:10.1038/s41467-023-40255-4)
Supplement: Supplementary file 1 — Supplementary Information [file 41467_2023_40255_MOESM1_ESM.pdf]

Supplementary Information

The Sharp Structural Switch of Covalent Cages Mediated by Subtle Variation of Directing Groups

Qiong Chen[1,4], Zhaoyong Li[1,3,4], Ye Lei[1,4], Yixin Chen[1], Hua Tang[1], Guangcheng Wu[1], Bin Sun[2], Yuxi Wei[1], Tianyu Jiao[1], Songna Zhang[2*], Feihe Huang[1,2*], Linjun Wang[1,3*], and Hao Li[1,2*]

[1]Department of Chemistry, Zhejiang University, Hangzhou 310058, P. R. China. [2]ZJU-Hangzhou Global Scientific and Technological Innovation Center, Zhejiang University, Hangzhou 311215, P. R. China. [3]Key Laboratory of Excited-State Materials of Zhejiang Province, Zhejiang University, Hangzhou 310058, P. R. China. [4]These authors contributed equally: Qiong Chen, Zhaoyong Li. Ye Lei. *Email: zsnchem@zju.edu.cn; fhuang@zju.edu.cn; ljwang@zju.edu.cn; lihao2015@zju.edu.cn

Table of Contents

1. Supplementary materials and general methods 3

2. Supplementary synthetic procedures 4

3. Self-assembly of products 15

4. Characterization of $\mathbf{1}_3\mathbf{S}_6$ 25

5. Characterization of $\mathbf{2}_2\mathbf{R}_2\mathbf{S}_2$ and $\mathbf{2}_2(EDA)_4$ 29

6. Characterization of $\mathbf{3}_2\mathbf{R}_2\mathbf{S}_2$, $\mathbf{3}_2(EDA)_4$ and $\mathbf{3}_3\mathbf{S}_6$ 36

7. Characterization of $\mathbf{4}_2\mathbf{R}_2\mathbf{S}_2$ and $\mathbf{4}_2(EDA)_4$ 46

8. Transformation between cages 52

9. The self-assembly yields of cages 55

10. X-ray Crystallography 64

11. Supplementary theoretical calculations 69

12. Supplementary References 70

1. Supplementary materials and general methods

All reagents and solvents were purchased from commercial sources and used without further purification. Terephthaloyl Chloride, bromobenzene, triethylsilane, trifluoromethanesulfonic acid, trifluoroacetic acid and bis(pinacolato)diboron were purchased from Energy Chemical platform. 4-Bromo-bromophenol, hexamethylenetetramine, 1-iodobutane, ethyl bromoacetate and tert-butyl bromoacetate were purchased from Bidepharm platform. $AlCl_3$, Pd(dppf)Cl_2, Cs_2CO_3, Pd(PPh_3)$_4$ and 1,2-diaminocyclohexane were purchased from Tansoole platform. KOAc, K_2CO_3 and hexamethylenetetramine were purchased from Sinopharm Chemical Reagent platform. The purity of all these commercially obtained chemicals is no less than 99% (≥99%). All solvents were purchased from Sinopharm Chemical Reagent platform and they are all analytically pure. Manipulations were performed under a normal laboratory atmosphere unless otherwise noted. Nuclear magnetic resonance (NMR) spectra were recorded at ambient temperature using Bruker AVANCE III 400, Bruker AVANCE III 500, or Agilent DD2 600 spectrometers, with working frequencies of 400/500/600 and 100/125/150 MHz for ^{1}H and ^{13}C, respectively. Chemical shifts are reported in ppm relative to the residual internal non deuterated solvent signals ($CDCl_3$: δ = 7.26 ppm, DMSO-d_6: δ = 2.50 ppm). High-resolution mass spectra (HRMS) were measured by using a SHIMADZU liquid chromatograph mass spectrometry ion trap time of flight (LCMS-IT-TOF) instrument and Bruker Daltonics Autoflex III (MALDI-TOF). X-ray crystallographic data were collected on a Bruker D8 Venture diffractometer. CD spectra were recorded on a Circular Dichroism Spectrometer (Chirascan V100, Applied Photophysics Ltd). IR-Spectra were recorded on a Thermofisher Nicolet IS50 FT-IR spectrometer. Elemental analysis was determined with a ThermoFisher FlashSmart Element Analyzer. PXRD measurement was taken on a Bruker D2 PHASER. Thermal gravimetric analysis was measured on a Discovery TGA 550 with a heating rate of 10 ^{o}C/min and a nitrogen flow of 50 mL/min.

2. Supplementary synthetic procedures

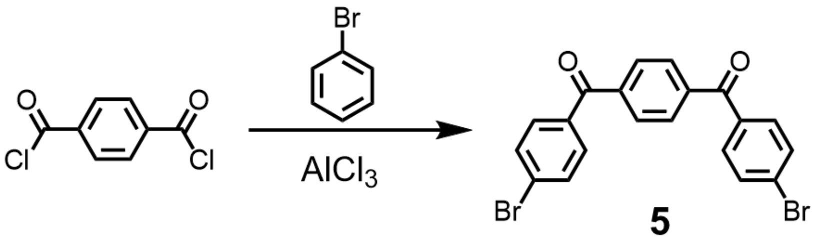

Supplementary Figure 1. Synthesis of 5.

5: Terephthaloyl chloride (404.0 mg, 2.0 mmol) and bromobenzene (10 mL) were mixed in a 25 mL round bottom flask. In a nitrogen atmosphere, $AlCl_3$ (532 mg, 4.0 mmol) was added into the flask slowly. The mixture was stirred at room temperature for 9 h. After that, it was heated at 90 °C for 2 h. After completion of reaction, the solution was cooled to room temperature and was poured into ice-cold methanol to form a precipitate, which was collected by filtration. The precipitate was underwent recrystallization by using methanol/chloroform to get the white solid **5** (671.8mg, 76%). **^{1}H NMR** (600 MHz, $CDCl_3$): δ = 7.87 (s, 4H), 7.70 (d, J = 6.0 Hz, 4H), 7.66 (d, J = 6.0 Hz, 4H). **^{13}C NMR** (150 MHz, $CDCl_3$): δ = 194.8, 140.5, 135.6, 131.9, 131.6, 139.7, 128.3. **HRMS**: *m/z* calculated for $C_{20}H_{12}Br_2O_2Na^+$ ($[M + Na]^+$): 464.9096; found: 464.9100.

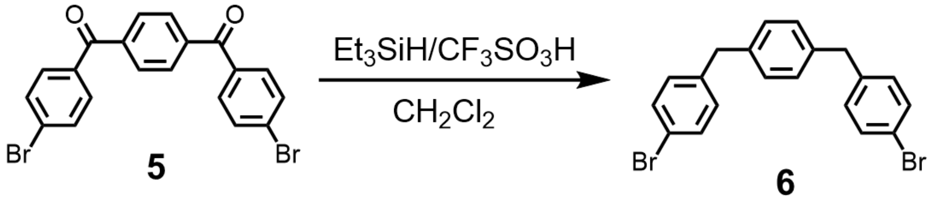

Supplementary Figure 2. Synthesis of 6.

6: **5** (441.9 mg, 1.0 mmol) was dissolved in 30 mL dry dichloromethane placed in a 50 mL round bottom flask. In a nitrogen atmosphere, Et_3SiH (1.5 mL) and CF_3SO_3H (0.8 mL) was added into the flask slowly. The mixture was stirred at room temperature overnight. After completion of reaction, the solution was quenched with $NaHCO_3$ aqueous solution

and extracted with DCM. The resulting organic layer was combined, and washed with water (3 x 50 mL), dried over anhydrous Na_2SO_4, and then concentrated to give the crude product. Purification by flash column chromatography (petroleum ether/ethyl acetate (10:1); silica gel, 200-300 mesh) yielded the product **6** as a white solid. (343.6mg, 83%). ^{1}**H NMR** (600 MHz, $CDCl_3$): δ = 7.38 (d, *J* = 6.0 Hz, 4H), 7.07 (s, 4H),7.04 (d, *J* = 6.0 Hz, 4H), 3.89 (s, 4H). ^{13}**C NMR** (150 MHz, $CDCl_3$): δ = 140.1, 138.5, 131.5, 130.6, 129.0, 120.0, 40.9. **HRMS**: *m/z* calculated for $C_{20}H_{16}Br_2Na^+$ ($[M + Na]^+$): 436.9511; found: 436.9512.

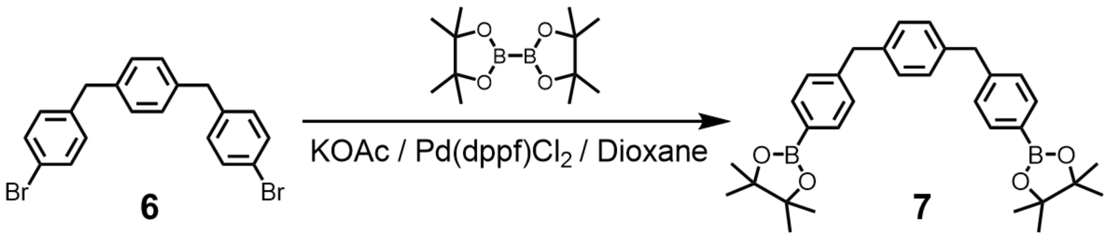

Supplementary Figure 3. Synthesis of 7.

7: **6** (414.0 mg, 1.0 mmol) and bis(pinacolato)diboron (558.7 mg, 2.2 mmol) were dissolved in 40 mL dioxane placed in a 100 mL round bottom flask. In a nitrogen atmosphere, KOAc (588.8 mg, 6.0 mmol) and Pd(dppf)Cl_2 (43.9 mg, 0.06 mmol) were then added into the flask. The mixture was heated at 90 °C for 12 h. After completion of reaction, the solution was cooled to room temperature and was poured into brine, which was then extracted with ethyl acetate. The resulting organic layer was combined, and washed with water (3 x 50 mL), dried over anhydrous Na_2SO_4, and then concentrated to give the crude product. Purification by flash column chromatography (petroleum ether/ethyl acetate (4:1); silica gel, 200-300 mesh) yielded the product **7** (397.9mg, 65%) as a white solid. ^{1}**H NMR** (400 MHz, $CDCl_3$): δ = 7.66 (d, *J* = 8.0 Hz, 4H), 7.12 (d, *J* = 8.0 Hz, 4H), 6.99 (s, 4H), 3.87 (s, 4H), 1.25 (s, 24H). ^{13}**C NMR** (100 MHz, $CDCl_3$): δ = 144.5, 138.7, 135.0, 129.0, 128.4, 83.7, 41.7, 24.9. **HRMS**: *m/z* calculated for $C_{32}H_{40}B_2O_4K^+$ ($[M + K]^+$): 549.2744; found: 549.2748.

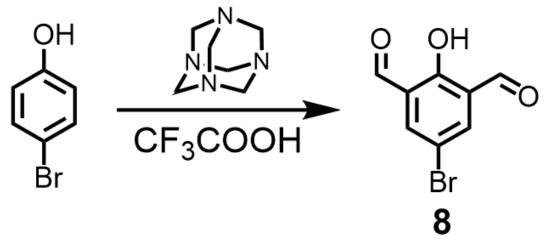

Supplementary Figure 4. Synthesis of 8.

8: 4-bromo-bromophenol (5.0 g, 29.0 mmol) and hexamethylenetetramine (30.0 g, 214.3 mmol) were dissolved in anhydrous CF_3COOH (100 mL) in a 250 mL round bottom flask. The yellow solution was heated at 110 °C for 5 d. After completion of reaction, the solution was cooled to room temperature and then poured into aqueous HCl (4 M, 500 mL). After that, the solution was stirred for 5 h at room temperature to obtain a precipitate, which was collected by filtration. The precipitate was washed with H_2O to obtain yellow solid **8** (5.3 g, 80 %). **^{1}H NMR** (600 MHz, $CDCl_3$): δ = 11.54 (s, 1H), 10.19 (s, 2H), 8.06 (s, 2H). **^{13}C NMR** (150 MHz, $CDCl_3$): δ = 191.0, 162.3, 139.8, 124.7, 112.2. **HRMS**: *m/z* calculated for $C_8H_5BrO_3Na^+$ ($[M + Na]^+$): 250.9314; found: 250.9310.

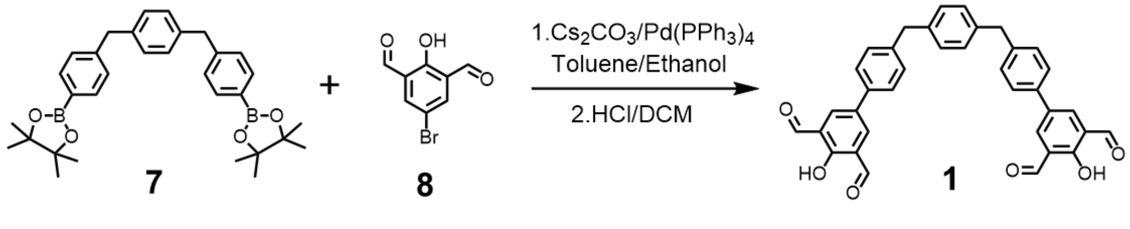

Supplementary Figure 5. Synthesis of 1.

1: In a 100mL round bottom flask, **7** (510.0 mg, 1.0 mmol) and **8** (501.6 mg, 2.2 mmol) were dissolved in the mixture of 40 ml toluene and 10 ml ethyl alcohol. In a nitrogen atmosphere, Cs_2CO_3 (1.4 g, 4.4 mmol) and $Pd(PPh_3)_4$ (115.5 mg, 0.1 mmol) were then added into the flask. The mixture was refluxed for 48 h at 105 °C. After completion of reaction, the solution was cooled to room temperature. The precipitate was collected via filtration and washed first with toluene. The solid was then washed with methanol. The methanol phases were collected, and the solvent was then removed via evaporation,

yielding a reddish brown solid. The solid was suspended in hydrochloric acid, and the corresponding aqueous suspension was extracted with DCM. After evaporating DCM, crude product was obtained, which underwent recrystallization by using n-hexane to get the light yellow solid **1** (273.9 mg, 50 %). **^{1}H NMR** (400 MHz, $CDCl_3$): δ = 11.60 (s, 2H), 10.30 (s, 4H), 8.15 (s, 4H), 7.51 (d, *J* = 8.0 Hz, 4H), 7.30 (d, *J* = 8.0 Hz, 4H), 7.16 (s, 4H), 4.01 (s, 4H). **^{13}C NMR** (100 MHz, $CDCl_3$): δ = 192.1, 162.8, 141.2, 138.7, 135.9, 135.7, 133.3, 129.7, 129.1, 126.7, 123.3, 41.1. **HRMS**: *m/z* calculated for $C_{36}H_{26}O_6$ Na^+ ([M + Na]$^+$): 557.1622; found: 557.1622. **Elemental anal**. calculated for $C_{36}H_{26}O_6$: C 77.97 H 4.73; found: C 76.85 H 4.56.

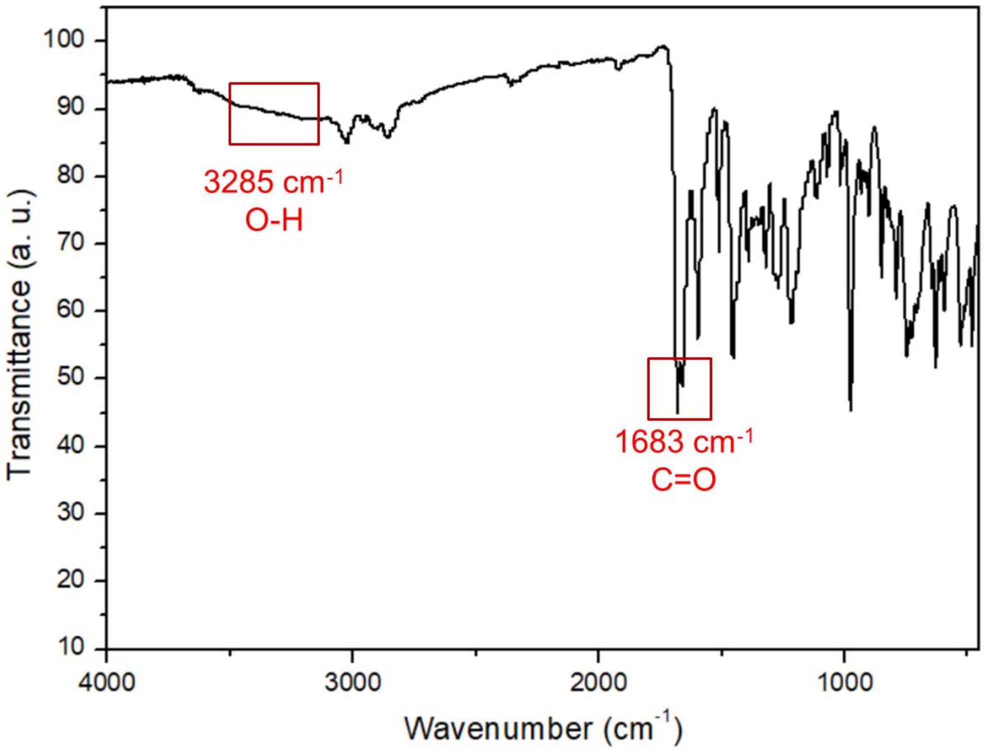

Supplementary Figure 6. FT-IR characterization. FT-IR spectrum of precursor **1**.

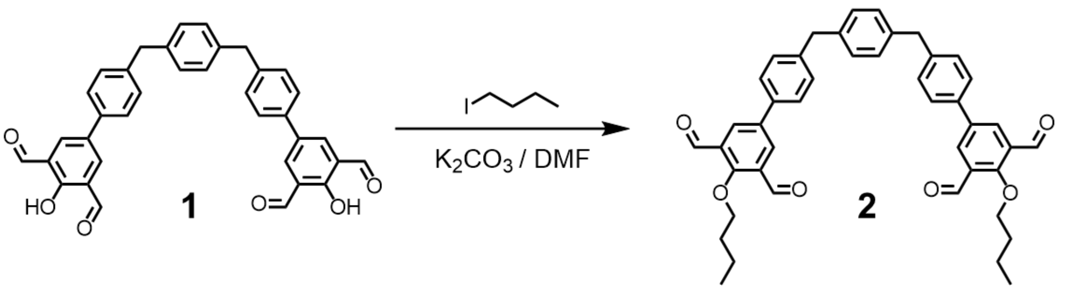

Supplementary Figure 7. Synthesis of 2.

2: **1** (277.0 mg, 0.5 mmol) and 1-Iodobutane (552.0 mg, 3 mmol) were dissolved in dry DMF (15 ml) placed in a 25 ml round bottom flask containing excess amount of K_2CO_3 (829.3 mg, 6 mmol). The reaction mixture was stirred at 60 °C for 12 h. After completion of reaction, the reaction mixture was cooled to room temperature and poured into water. The aqueous mixture was then extracted with ethyl acetate. The resulting organic layers were combined, and washed with water (3 x 50 mL), dried over anhydrous Na_2SO_4. Evaporation of the solvent yielded the crude product. Purification by flash column chromatography (dichloromethane; silica gel, 200-300 mesh) yielded the pure product **2** (159.8mg, 48%) as a white solid. **^{1}H NMR** (400 MHz, $CDCl_3$): δ = 10.37 (s, 4H), 8.22 (s, 4H), 7.47 (d, *J* = 8.0 Hz, 4H), 7.22 (d, *J* = 8.0 Hz, 4H), 7.08 (s, 4H), 4.08 (t, *J* = 8.0 Hz, 4H), 3.93 (s, 4H), 1.82 (m, 4H), 1.47 (m, 4H), 0.94 (t, *J* = 8.0 Hz, 6H). **^{13}C NMR** (100 MHz, $CDCl_3$): δ = 188.7, 163.9, 141.5, 138.7, 137.7, 136.0, 132.7, 130.4, 129.7, 129.1, 127.0, 80.6, 41.2, 32.0, 19.1, 13.9. **HRMS**: *m/z* calculated for $C_{44}H_{42}O_6Na^+$ ($[M + Na]^+$): 689.2874; found: 689.2875. **Elemental anal**. calculated for $C_{44}H_{42}O_6$: C 79.26 H 6.35; found: C 79.21 H 6.42.

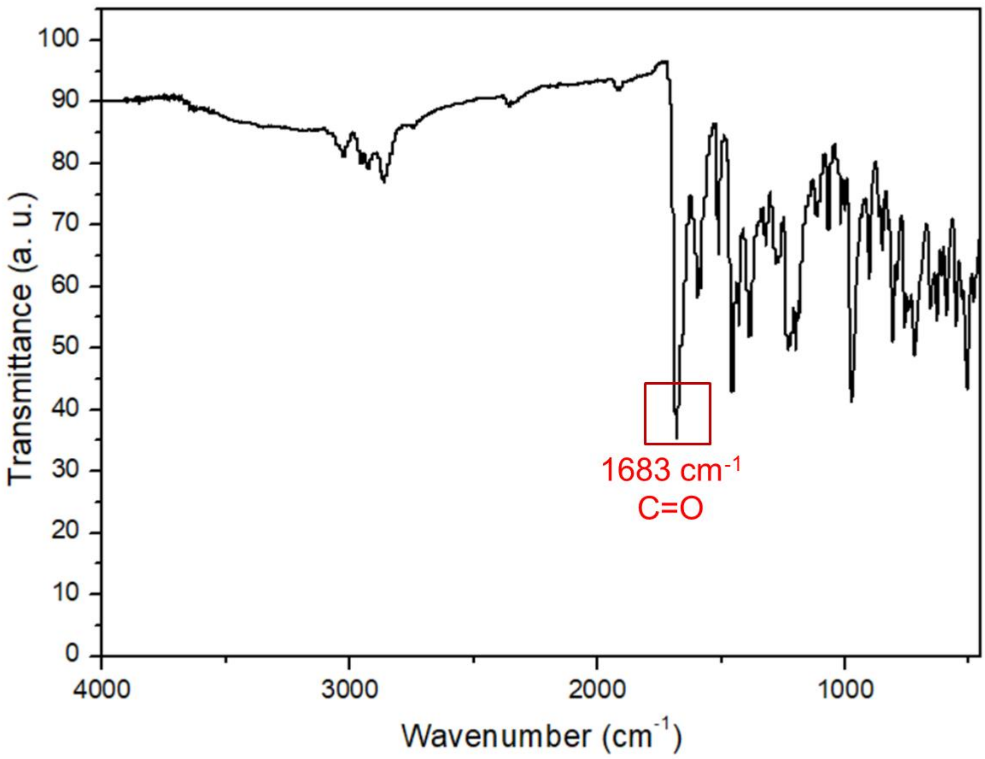

Supplementary Figure 8. FT-IR characterization. FT-IR spectrum of precursor **2**.

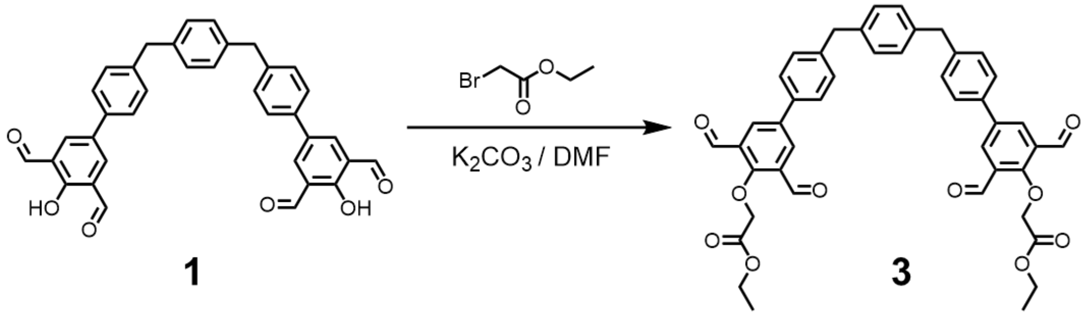

Supplementary Figure 9. Synthesis of 3.

3: **1** (277.0 mg, 0.5 mmol) and ethyl bromoacetate (501.0 mg, 3 mmol) were dissolved in dry DMF (15 ml) placed in a 25 ml round bottom flask containing excess amount of K_2CO_3 (829.3 mg, 6 mmol). The reaction mixture was stirred at 50 °C for 12 h. After completion of reaction, the solution was cooled to room temperature and poured into water. The aqueous mixture was then extracted with ethyl acetate. The resulting organic layers were combined, and washed with water (3 x 50 mL), dried over anhydrous Na_2SO_4. Evaporation of the solvent yielded the crude product. Purification by flash column

chromatography (dichloromethane; silica gel, 200-300 mesh) yielded the pure product **3** (152.4 mg, 42%) as a white solid. **^{1}H NMR** (600 MHz, $CDCl_3$): δ = 10.05 (s, 4H), 8.28 (s, 4H), 7.54 (d, *J* = 6.0 Hz, 4H), 7.30 (d, *J* = 6.0 Hz, 4H), 7.15 (s, 4H), 4.82 (s, 4H), 4.24 (q, *J* = 6.0 Hz, 4H), 4.01 (s, 4H), 1.28 (t, *J* = 6.0 Hz, 6H). **^{13}C NMR** (150 MHz, $CDCl_3$): δ = 191.6, 170.9, 163.8, 144.3, 141.3, 140.8, 138.4, 136.6, 132.7, 132.4, 131.8, 129.7, 76.2, 64.4, 43.8, 16.8. **HRMS**: *m/z* calculated for $C_{44}H_{38}O_{10}Na^+$ ($[M + Na]^+$): 749.2357; found: 749.2358. **Elemental anal**. calculated for $C_{44}H_{38}O_{10}$: C 72.72 H 5.27; found: C 72.58 H 5.48.

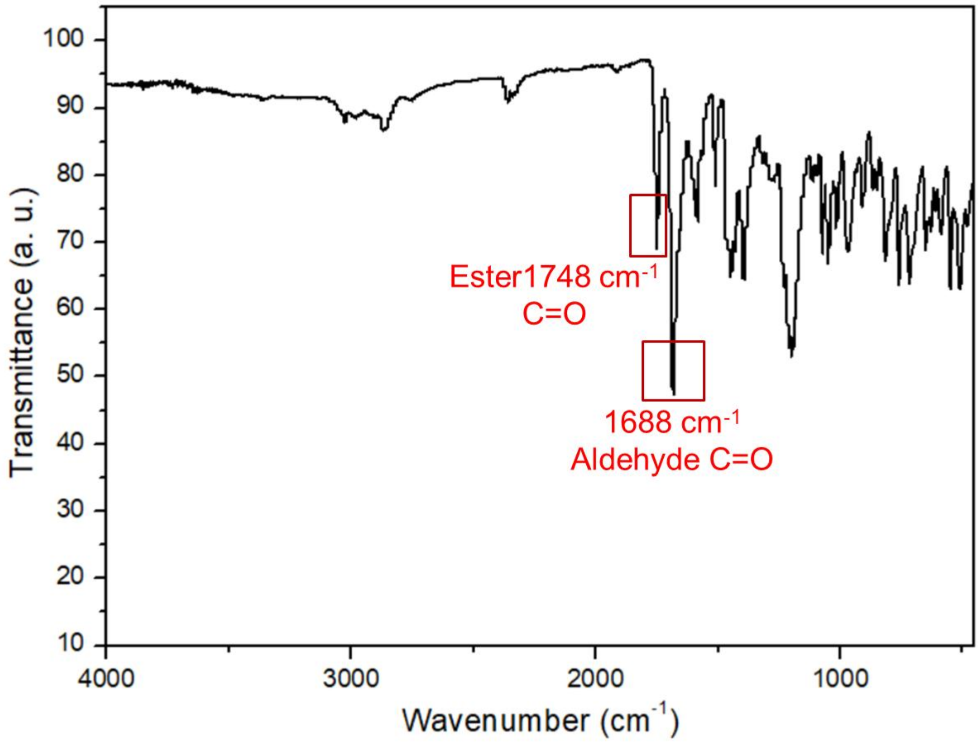

Supplementary Figure 10. FT-IR characterization. FT-IR spectrum of precursor **3**.

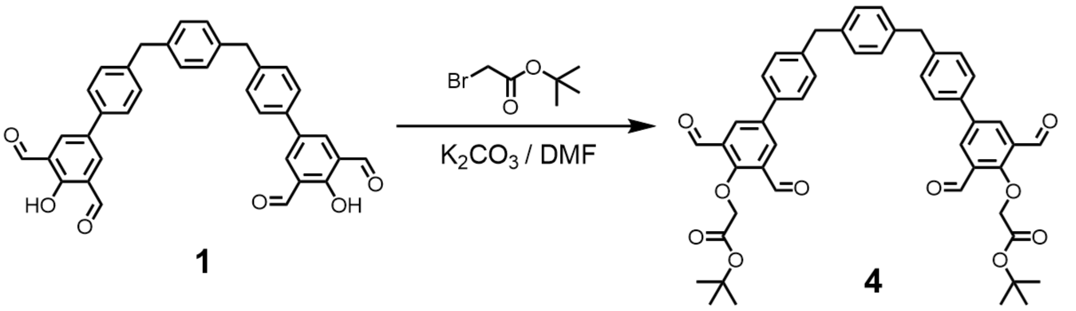

Supplementary Figure 11. Synthesis of 4.

4: **1** (277.0 mg, 0.5 mmol) and tert-butyl bromoacetate (582.0 mg, 3 mmol) were dissolved in dry DMF (15 ml) placed in a 25 ml round bottom flask containing excess amount of K_2CO_3 (829.3 mg, 6 mmol). The reaction mixture was stirred at 50 °C for 12 h. After completion of reaction, the solution was cooled to room temperature and poured into water. The aqueous mixture was extracted with ethyl acetate. The combined organic layers were washed with water and dried over anhydrous Na_2SO_4. Evaporation of the solvent produced the crude product. Purification by flash column chromatography (dichloromethane; silica gel, 200-300 mesh) yielded product **4** (156.3 mg, 40%) as a white solid. **^{1}H NMR** (400 MHz, $CDCl_3$): δ = 10.51 (s, 4H), 8.28 (s, 4H), 7.54 (d, *J* = 8.0 Hz, 4H), 7.29 (d, *J* = 8.0 Hz, 4H), 7.15 (s, 4H), 4.70 (s, 4H), 4.01 (s, 4H), 1.48 (s, 18H). **^{13}C NMR** (100 MHz, $CDCl_3$): δ = 189.1, 167.1, 161.7, 141.6, 138.6, 138.0, 135.8, 133.7, 130.0, 129.7, 129.1, 127.0, 83.2, 74.2, 41.2, 28.1. **HRMS**: *m/z* calculated for $C_{48}H_{46}O_{10}Na^+$ ($[M + Na]^+$): 805.2983; found: 805.2984. **Elemental anal**. calculated for $C_{48}H_{46}O_{10}$: C 73.65 H 5.92; found: C 73.44 H 5.80.

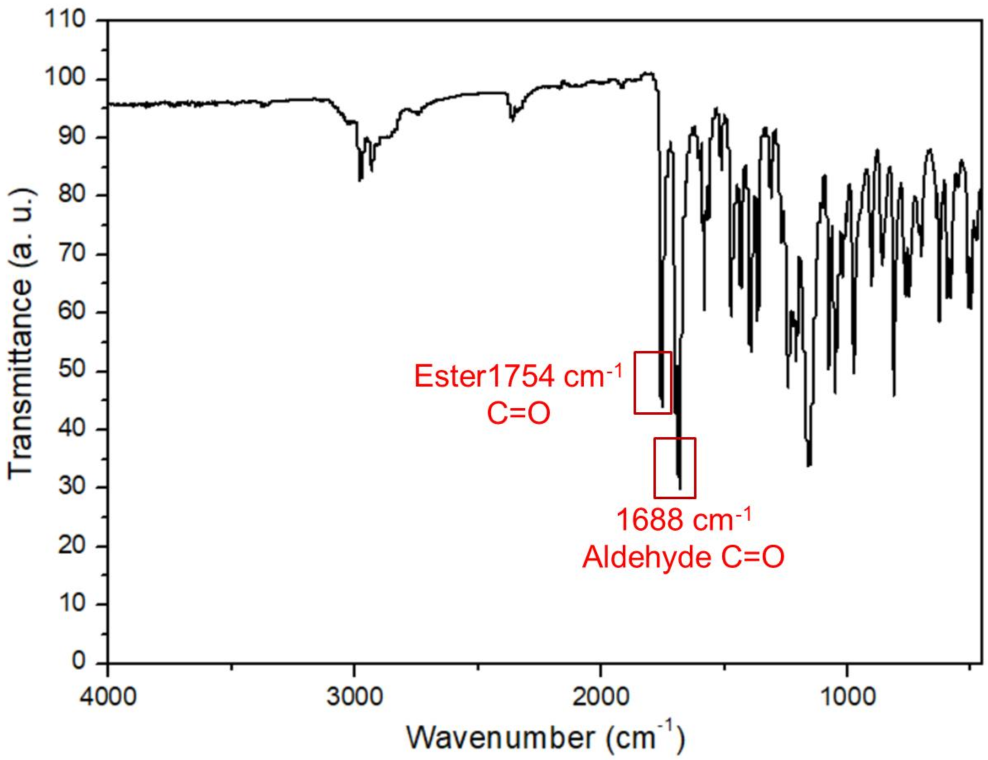

Supplementary Figure 12. FT-IR characterization. FT-IR spectrum of precursor **4**.

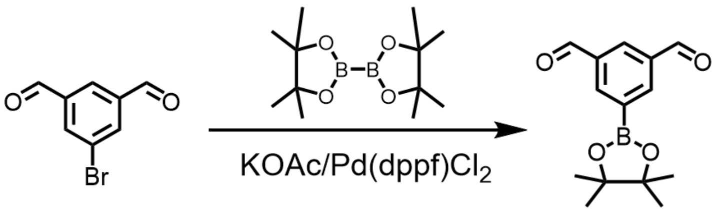

Supplementary Figure 13. Synthesis of 9.

9: 5-Bromobenzene-1,3-dicarbaldehyde (424.0 mg, 2.0 mmol) and bis(pinacolato)diboron (1.1g, 4.2 mmol) were dissolved in dioxane (50 mL) placed in a 100 mL round bottom flask. In a nitrogen atmosphere, KOAc (588.8 mg, 6.0 mmol) and Pd(dppf)Cl_2 (43.9 mg, 0.06 mmol) were then added into the flask. The mixture was heated at 90 °C for 12 h. After completion of reaction, the solution was cooled to room temperature and poured into brine, which was then extracted with ethyl acetate. The resulting organic layer was combined, and washed with water (3 x 50 mL), dried over anhydrous Na_2SO_4, and then concentrated to give the crude product. Purification by flash column chromatography

(petroleum ether/ethyl acetate (4:1); silica gel, 200-300 mesh) yielded the product **9** (405.6 mg, 78%) as a white solid. **^{1}H NMR** (600 MHz, $CDCl_3$): δ = 10.13 (s, 2H), 8.55 (s, 2H), 8.46 (s, 1H), 1.38 (s, 12H) **^{13}C NMR** (150 MHz, $CDCl_3$): δ = 191.3, 141.4, 136.4, 132.2, 84.8, 25.0. **HRMS**: *m/z* calculated for $C_{14}H_{17}BO_4Na^+$ ($[M + K]^+$): 283.1118; found: 283.1120.

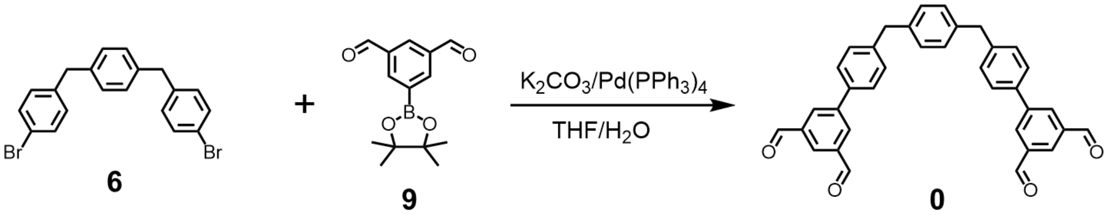

Supplementary Figure 14. Synthesis of 0.

0: **6** (414.0 mg, 1.0 mmol) and **9** (1.0 g, 4.0 mmol) were dissolved in the mixture of THF (40 ml) and H_2O (10 ml) placed in a 100 ml round bottom flask. In a nitrogen atmosphere, K_2CO_3 (829.3 mg, 6.0 mmol) and $Pd(PPh_3)_4$ (115.5 mg, 0.1 mmol) were then added into the flask. The mixture was heated at 80 °C for 24 h. After completion of reaction, the solution was cooled to room temperature. After vacuum evaporation of THF, the residue was poured into brine, which was then extracted with DCM by three times. The combined organic layers were washed with water and dried over anhydrous Na_2SO_4. Evaporation of the solvent produced the crude product. Purification by flash column chromatography (petroleum ether/ethyl acetate (4:1); silica gel, 200-300 mesh) yielded the white solid-state product **0** (172.3 mg, 33%). **^{1}H NMR** (600 MHz, $CDCl_3$): δ = 10.16 (s, 4H), 8.34 (s, 4H), 8.32 (s, 2H), 7.60 (d, *J* = 6.0 Hz, 4H), 7.34 (d, *J* = 6.0 Hz, 4H), 7.18 (s, 4H), 4.03 (s, 4H). **^{13}C NMR** (125 MHz, $CDCl_3$): δ = 191.4, 143.3, 142.3, 138.9, 137.8, 136.4, 133.0, 130.1, 129.9, 129.4, 127.5, 41.4. **HRMS**: *m/z* calculated for $C_{36}H_{26}O_4Na^+$ ($[M + Na]^+$): 545.1723; found: 545.1725. **Elemental anal**. calculated for $C_{36}H_{26}O_4$: C 82.74 H 5.01; found: C 82.79 H 5.11.

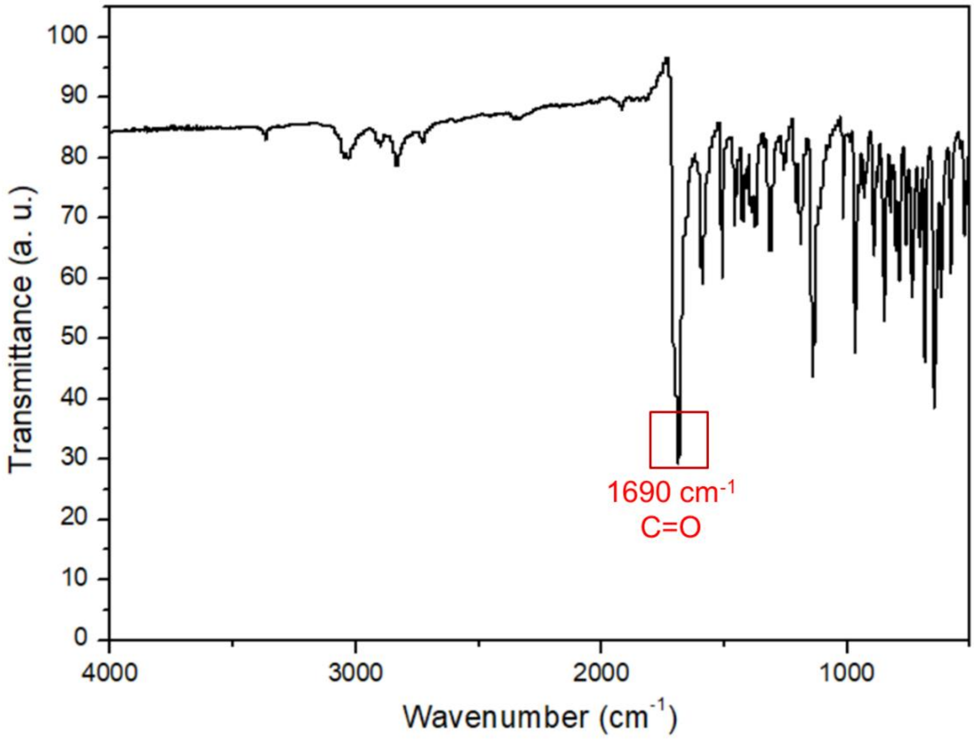

Supplementary Figure 15. FT-IR characterization. FT-IR spectrum of precursor **0**.

3. Self-assembly of products

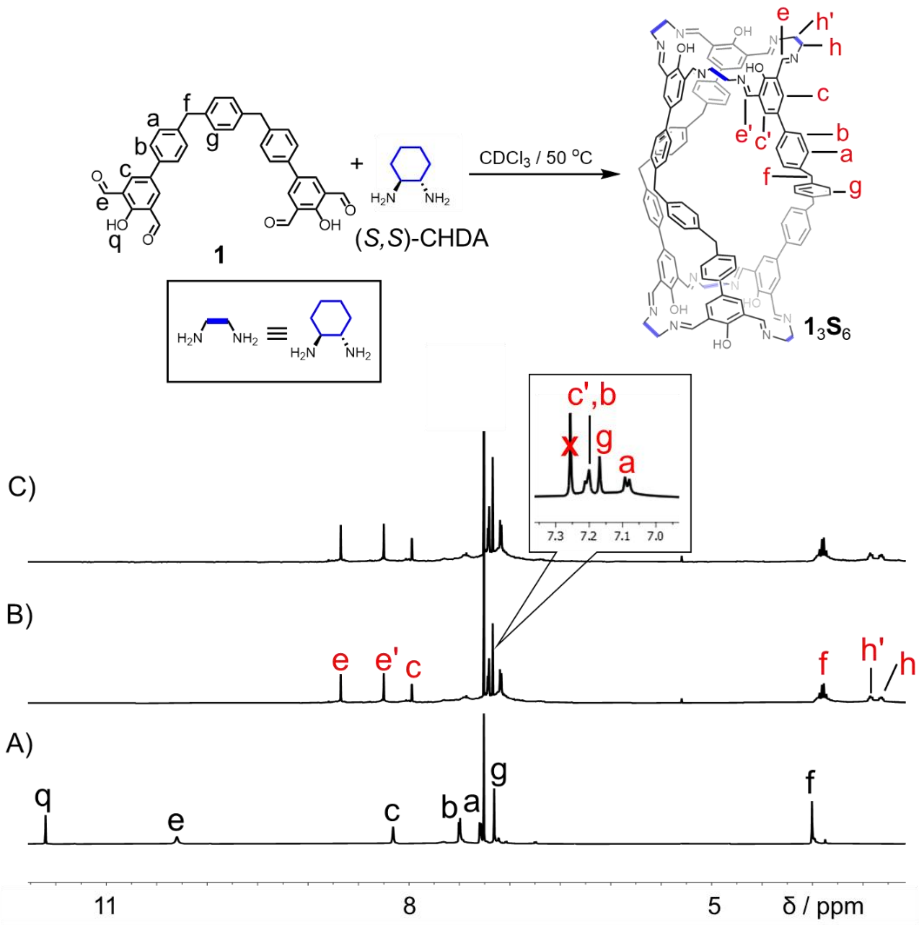

Supplementary Figure 16. NMR characterization. Partial ^{1}H NMR spectra (600 MHz, $CDCl_3$, 298 K) of A) **1**, as well as the reaction solutions by condensing a 1:2 mixture of **1** and the corresponding bisamine including B) (*S,S*)-CHDA and C) (*R,R*)-CHDA.

A 1:2 mixture of **1** (2.77 mg, 0.005 mmol) and (*S,S*)-CHDA (1.14 mg, 0.01 mmol) was combined and dissolved in $CDCl_3$ (2 mL). After heating the corresponding solutions at 50 ^{o}C for 6 h, the ^{1}H NMR and mass spectrum of the corresponding solution was recorded. It was demonstrated that a [3+6] cage $\mathbf{1_3S_6}$ was produced as the major product (Supplementary Figure 16B), which was fully characterized in $CDCl_3$ by ^{1}H NMR spectroscopy, 2D NMR spectroscopy, as well as mass spectrometry (Supplementary Figures 28-32). The yield of $\mathbf{1_3S_6}$ was determined to be 60 % (Supplementary Figure 76), calculated by using an internal standard in the corresponding ^{1}H NMR sample.

When we use (*R,R*)-CHDA instead of (*S,S*)-CHDA, a [3+6] cage **1**$_3$**R**$_6$ was obtained, whose ^{1}H NMR spectrum (Supplementary Figure 16C) is almost identical as that of **1**$_3$**S**$_6$. The CD spectra of **1**$_3$**R**$_6$ and **1**$_3$**S**$_6$ show mirror images (Supplementary Figure 33). However, when we use ethylenediamine (EDA) instead of (*S,S*)-CHDA, a bunch of precipitates would be obtained, indicating the failure of self-assembly.

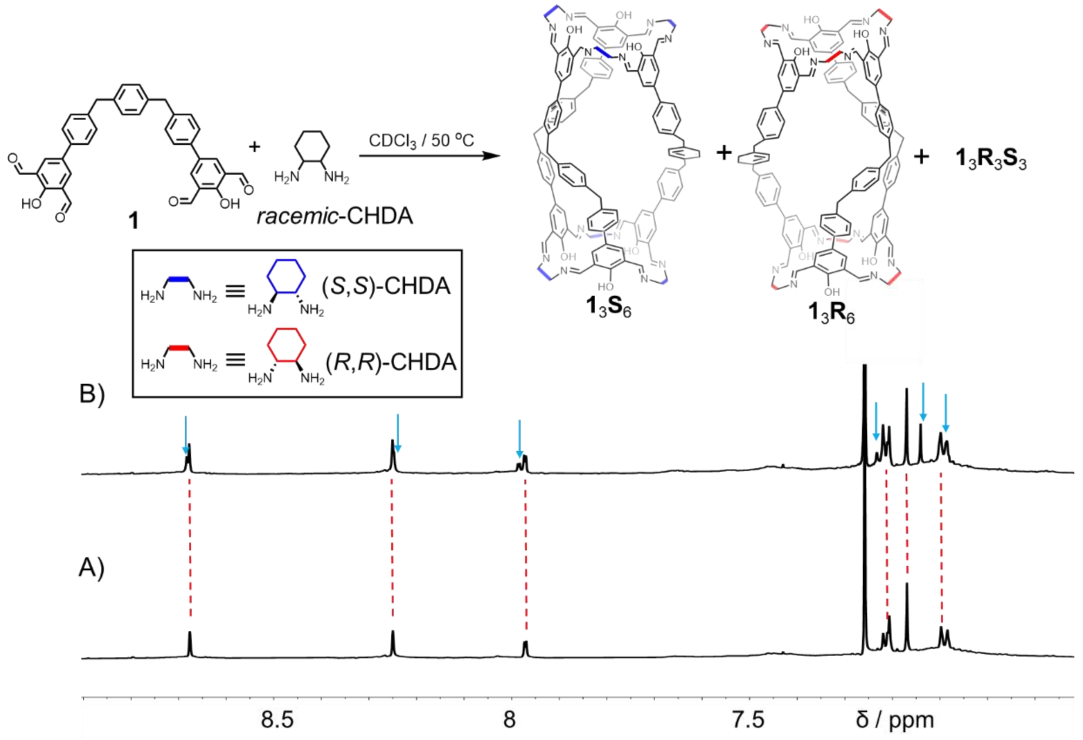

Supplementary Figure 17. NMR characterization. Partial ^{1}H NMR spectra (600 MHz, $CDCl_3$, 298 K) of A) **1**$_3$**S**$_6$ and B) the reaction mixture by condensing a 1:2 mixture of **1** and racemic *trans*-CHDA.

A 1:2 mixture of **1** (2.77 mg, 0.005 mmol) and racemic *trans*-CHDA (1.14 mg, 0.01 mmol) was combined and dissolved in $CDCl_3$ (2 mL). After heating the corresponding solutions at 50 ^{o}C for 6 h, the ^{1}H NMR spectrum of the corresponding solution was recorded (Supplementary Figure 17B). There are two sets of peaks in the ^{1}H NMR spectrum. One set of major peaks is identical as the resonances of **1**$_3$**S**$_6$ or **1**$_3$**R**$_6$, labelled with red dotted lines in Supplementary Figure 17. Another set of minor resonances might be assigned to a compound **1**$_3$**S**$_3$**R**$_3$, which are labelled with blue arrows in Supplementary Figure 17B.

The CD spectrum of the product from racemic *trans*-CHDA is CD silent (Supplementary Figure 33).

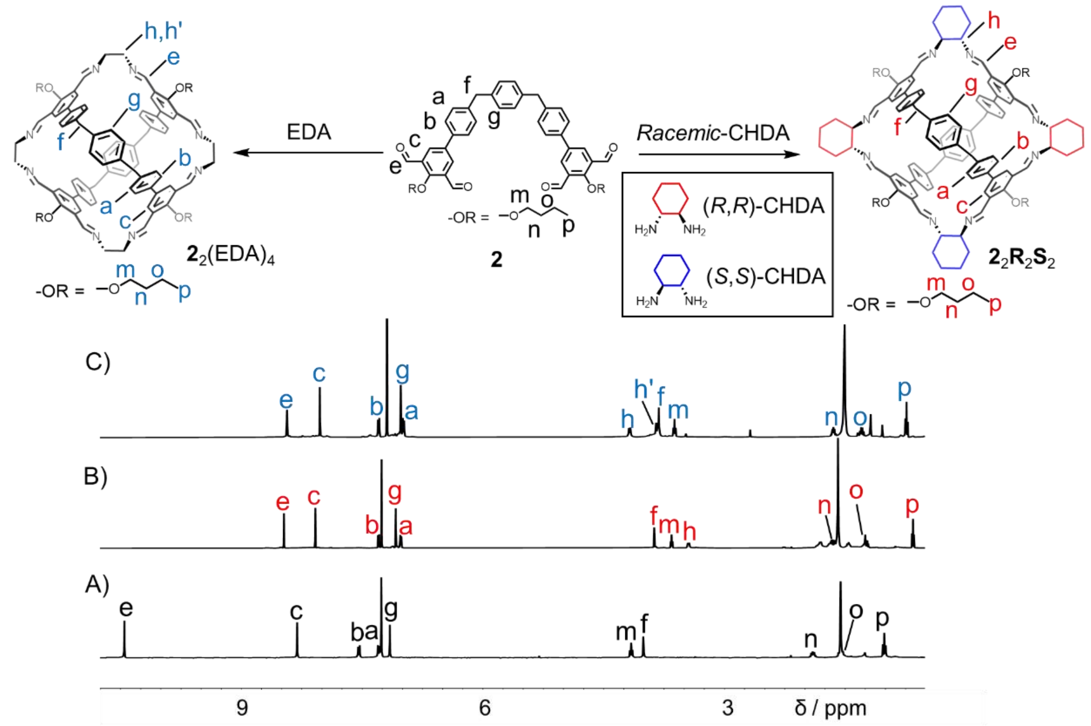

Supplementary Figure 18. NMR characterization. Partial ^{1}H NMR spectra (600 MHz, $CDCl_3$, 298 K) of A) **2**, as well as the reaction mixtures by condensing a 1:2 mixture of **2** and a bisamine partner, namely either B) racemic *trans*-CHDA, or C) EDA.

A 1:2 mixture of **2** (3.33 mg, 0.005 mmol) and racemic *trans*-CHDA (1.14 mg, 0.01 mmol) was combined and dissolved in $CDCl_3$ (2 mL). After heating the corresponding solutions at 50 °C for 6 h, the ^{1}H NMR and mass spectra of the corresponding solution were recorded. It was demonstrated that an achiral cage $\mathbf{2_2R_2S_2}$ was produced as the major product (Supplementary Figure 18B), which was fully characterized in $CDCl_3$ by ^{1}H NMR spectroscopy, 2D NMR spectroscopy, as well as mass spectrometry (Supplementary Figures 34-38). The NMR yield of $\mathbf{2_2R_2S_2}$ was determined to be 57% (Supplementary Figure 77).

Another achiral cage $\mathbf{2_2}$(EDA)$_4$ was obtained in a 50% yield (Supplementary Figure 78), by using a similar procedure as that of $\mathbf{2_2R_2S_2}$, in which EDA was used as the bisamino

partner to (Supplementary Figure 18C). It was fully characterized in $CDCl_3$ by ^1H NMR spectroscopy, 2D NMR spectroscopy, as well as mass spectrometry (Supplementary Figures 42-46).

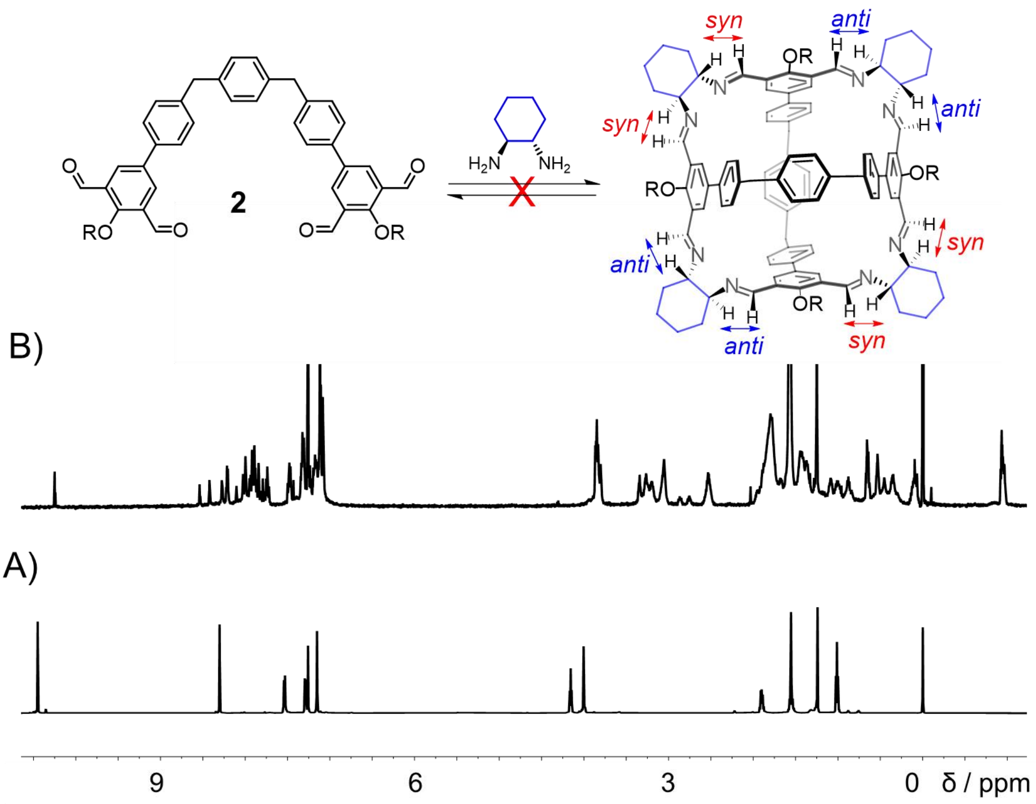

Supplementary Figure 19. NMR characterization. Partial ^1H NMR spectra (600 MHz, $CDCl_3$, 298 K) of A) **2**, B) reaction mixture by condensing **2** and (*S,S*)-CHDA in the ratio of 2:4.

When **2** and (*S,S*)-CHDA were combined and dissolved in $CDCl_3$ in a 2:4 ratio, the ^1H NMR spectrum became quite complicated (Supplementary Figure 19B). The resonances corresponding to unreacted aldehyde units were still observed, indicating that the self-assembly of a putative cage $\mathbf{2}_2\mathbf{S}_4$ was unsuccessful.

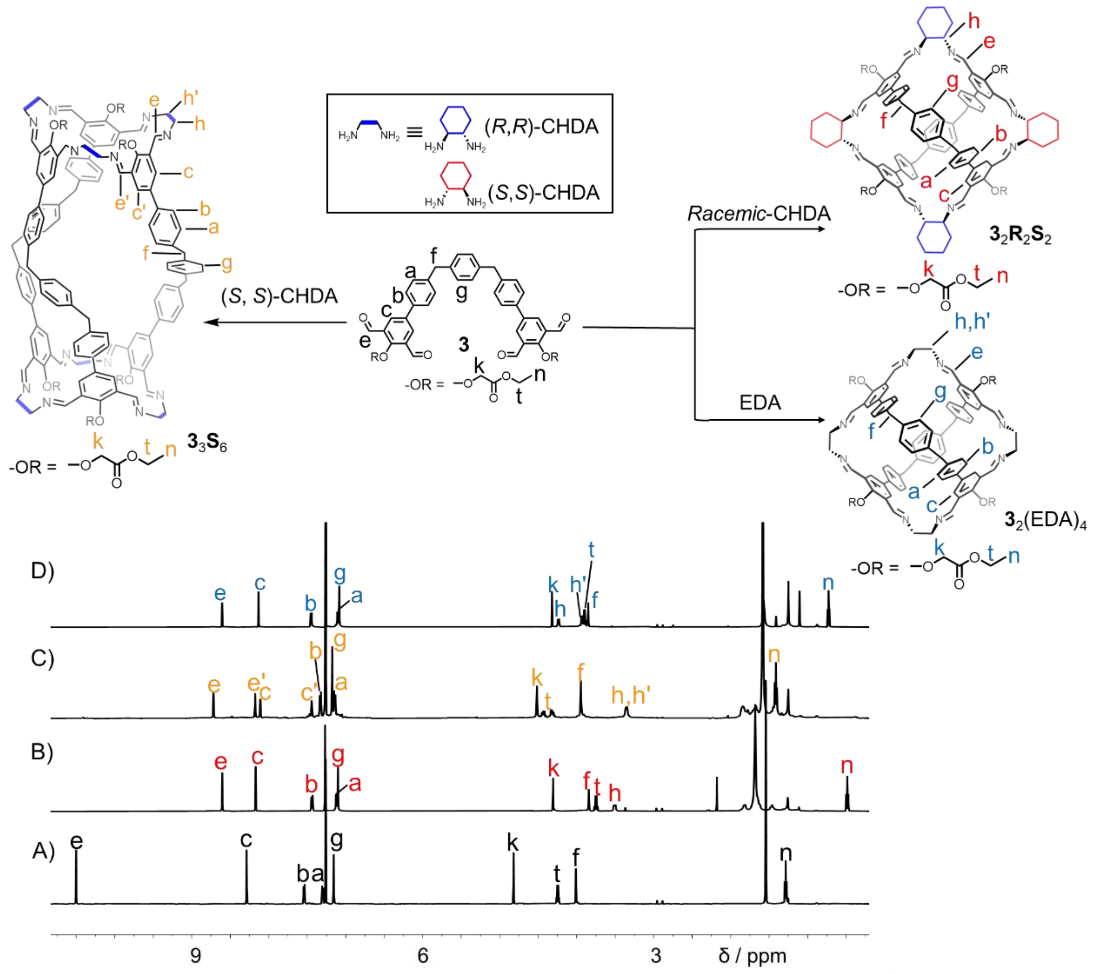

Supplementary Figure 20. NMR characterization. Partial ^{1}H NMR spectra (600 MHz, $CDCl_3$, 298 K) of A) **3**, as well as a series of 1:2 reaction mixtures of 3 and different bisamino partners including B) racemic *trans*-CHDA, C) (*S,S*)-CHDA, and D) EDA.

A 1:2 mixture of **3** (3.63 mg, 0.005 mmol) and racemic *trans*-CHDA (1.14 mg, 0.01 mmol) was combined and dissolved in $CDCl_3$ (2 mL). After heating the corresponding solutions at 50 °C for 6 h, the ^{1}H NMR and mass spectra of the corresponding solutions were recorded. It was demonstrated that an achiral cage $\mathbf{3_2R_2S_2}$ was produced as the major product (Supplementary Figure 20B), which was fully characterized in $CDCl_3$ by ^{1}H NMR spectroscopy, 2D NMR spectroscopy, as well as mass spectrometry (Supplementary Figures 47-51). The NMR yield of $\mathbf{3_2R_2S_2}$ was determined to be 75% (Supplementary Figure 79). When we use (*S,S*)-CHDA instead of *racemic*-CHDA, a chiral [3+6] cage $\mathbf{3_3S_6}$ was obtained (Supplementary Figure 20C). The NMR yield of $\mathbf{3_3S_6}$ was determined to be

63% (Supplementary Figure 81).

An achiral cage **3**$_2$(EDA)$_4$ was obtained in 85 % yield (Supplementary Figure 80), by combining **3** and EDA in a 1:2 ratio in $CDCl_3$. It was fully characterized in $CDCl_3$ by ^{1}H NMR spectroscopy, 2D NMR spectroscopy, as well as mass spectrometry (Supplementary Figures 46-50).

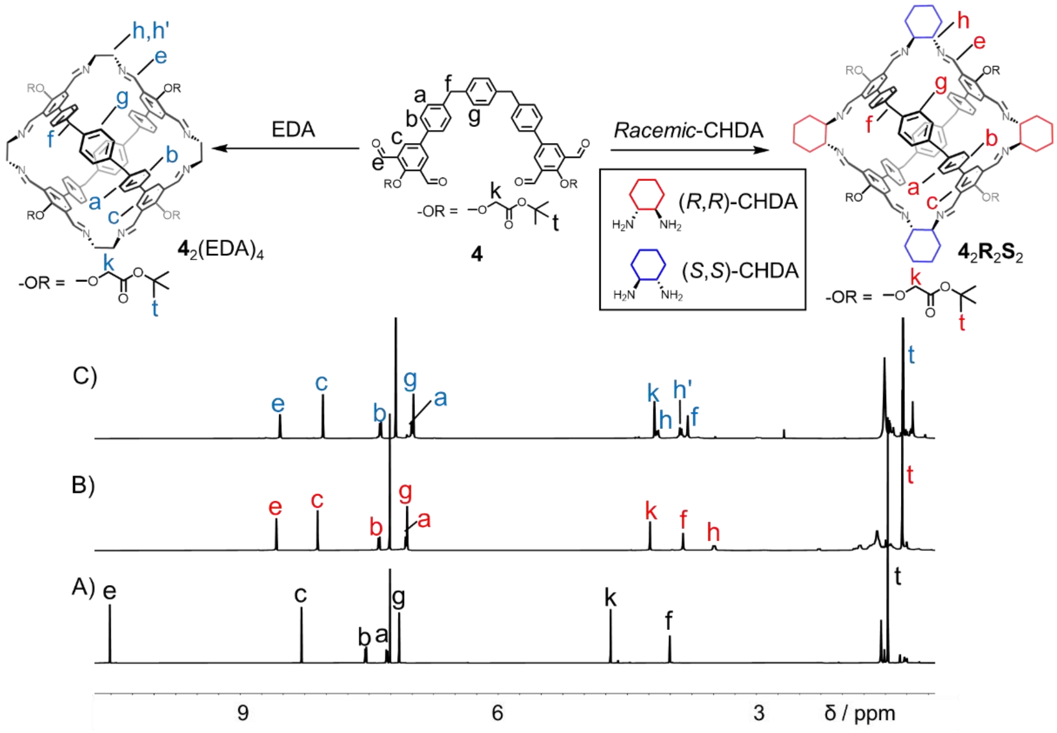

Supplementary Figure 21. NMR characterization. Partial ^{1}H NMR spectra (600 MHz, $CDCl_3$, 298 K) of A) **4**, and two 1:2 reaction mixtures by condensing **4** and different biamino partners including B) racemic *trans*-CHDA and C) EDA.

Two achiral cages **4**$_2$**R**$_2$**S**$_2$ and **4**$_2$(EDA)$_4$ were obtained. They were fully characterized in $CDCl_3$ by ^{1}H NMR spectroscopy, 2D NMR spectroscopy, as well as mass spectrometry (Supplementary Figures 57-66).

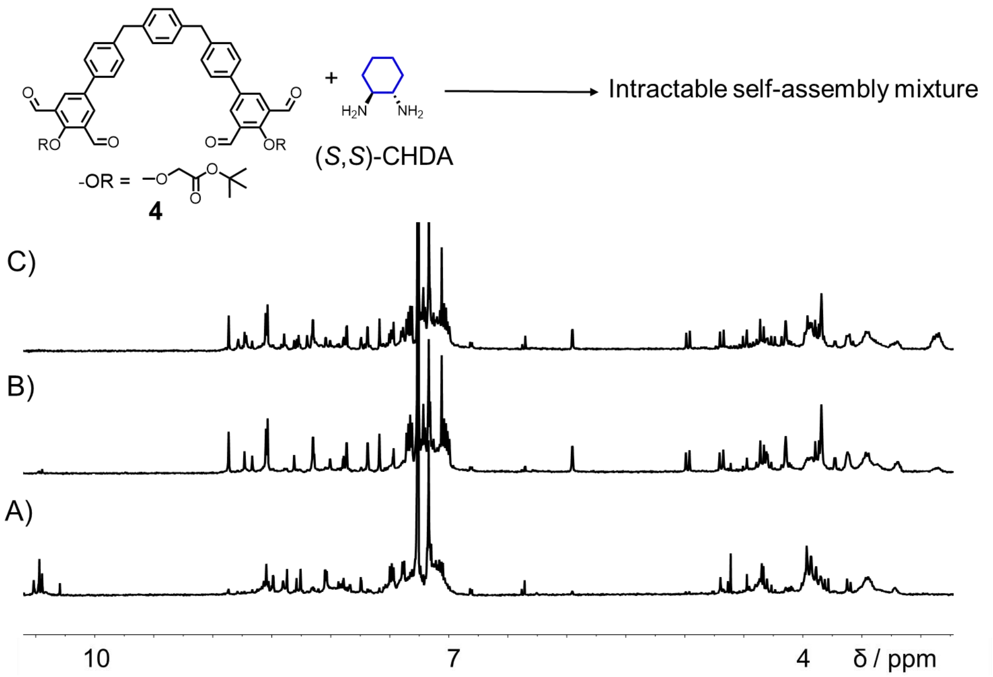

Supplementary Figure 22. NMR characterization. Partial ^{1}H NMR spectra (500 MHz, $CDCl_3$, 298 K) of the reaction mixtures of **4** and (*S,S*)-CHDA in different ratios including A) 2:3, B) 2:4 and C) 2:5.

Condensing **4** and (*S,S*)-CHDA yielded a library of intractable mixtures (Supplementary Figure 22), whose structures are not well understood at the present time.

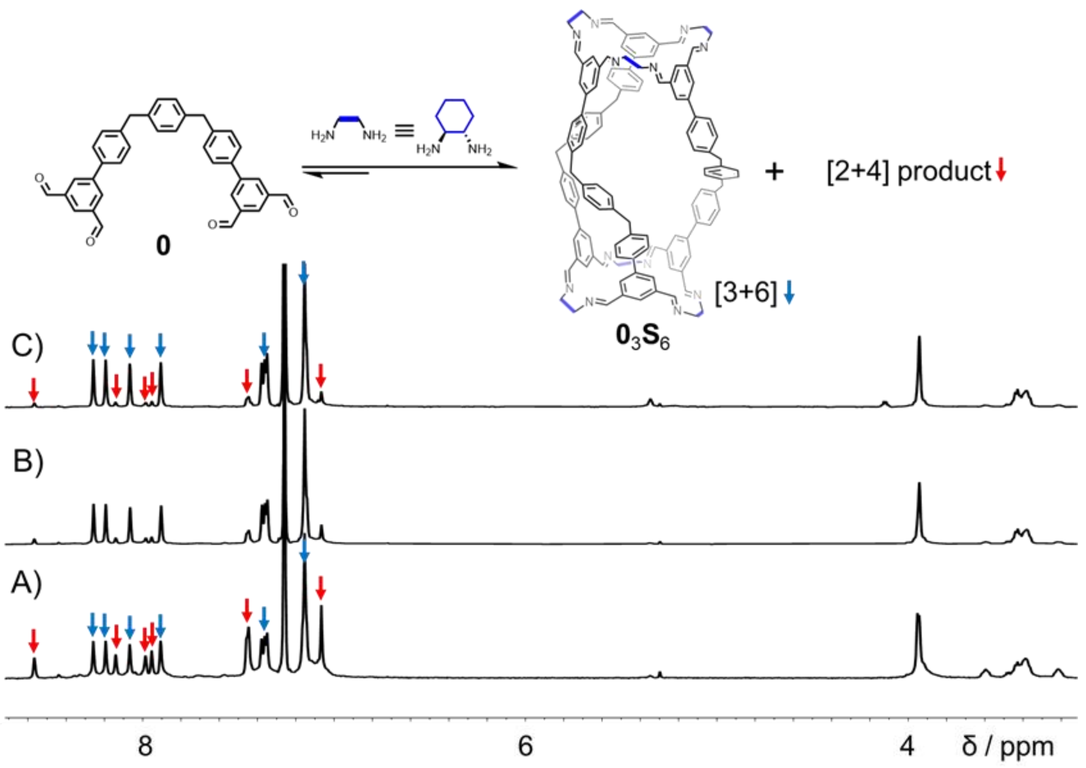

Supplementary Figure 23. NMR characterization. Partial ^{1}H NMR spectra (400 MHz, $CDCl_3$, 298 K) of products by condensing **0** and (*S,S*)-CHDA in a 1:2 ratio at 50 ^oC with different reaction time, including A) 4 h, B) 24 h and C) 120 h. The resonances of [3+6] cage and [2+4] product are marked with blue and red arrows, respectively.

A 1:2 mixture of **0** (3.13 mg, 0.006 mmol) and (*S,S*)-CHDA (1.37 mg, 0.012 mmol) was combined and dissolved in $CDCl_3$ (2 mL). After heating the corresponding solutions at 50 ^oC for 4 h, 24 h and 120 h, respectively, the ^{1}H NMR spectra (Supplementary Figure 23) of the corresponding solutions were recorded. It was demonstrated that a chiral [3+6] cage **0_3S_6** was produced as the major product, accompanied with a [2+4] product as a kinetic product. As the reaction proceeding, the [2+4] kinetic product would transfer to the [3+6] chiral cage, which is fully convinced by ^{1}H NMR spectra and mass spectra (Supplementary Figures 17-19). The conversion was not complete even after the solution was heated by 120 h. This phenomenon indicates that the absence of directing groups in the precursor **0** resulted from the system requiring a longer reaction time to reach equilibrium. The NMR yield of **0_3S_6** was determined to be 70% (Supplementary Figure 84) by using internal standard.

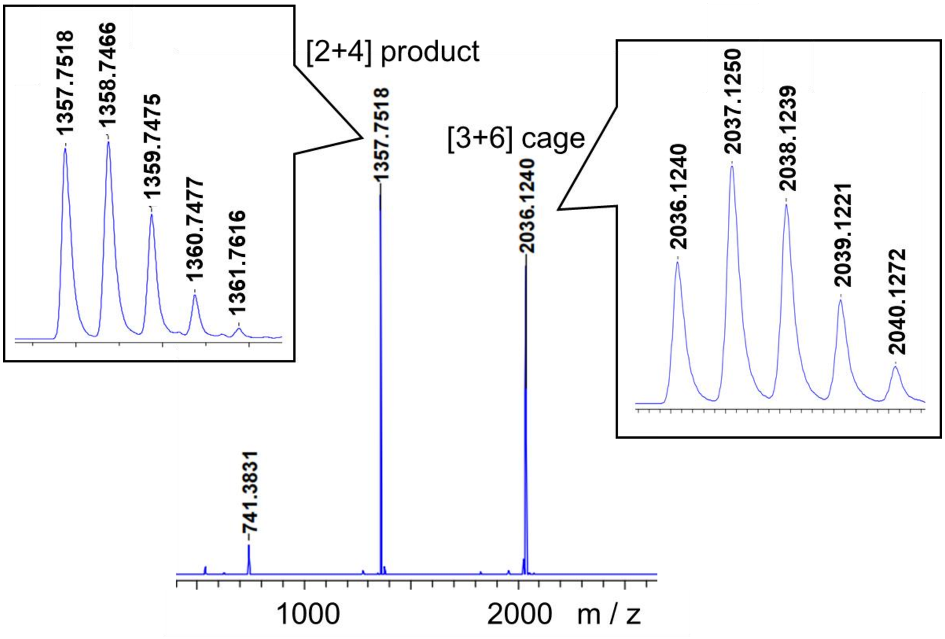

Supplementary Figure 24. MS characterization. High-resolution MALDI-TOF of the self-assembly product by condensing **0** and (*S,S*)-CHDA at 50 °C for 4 h.

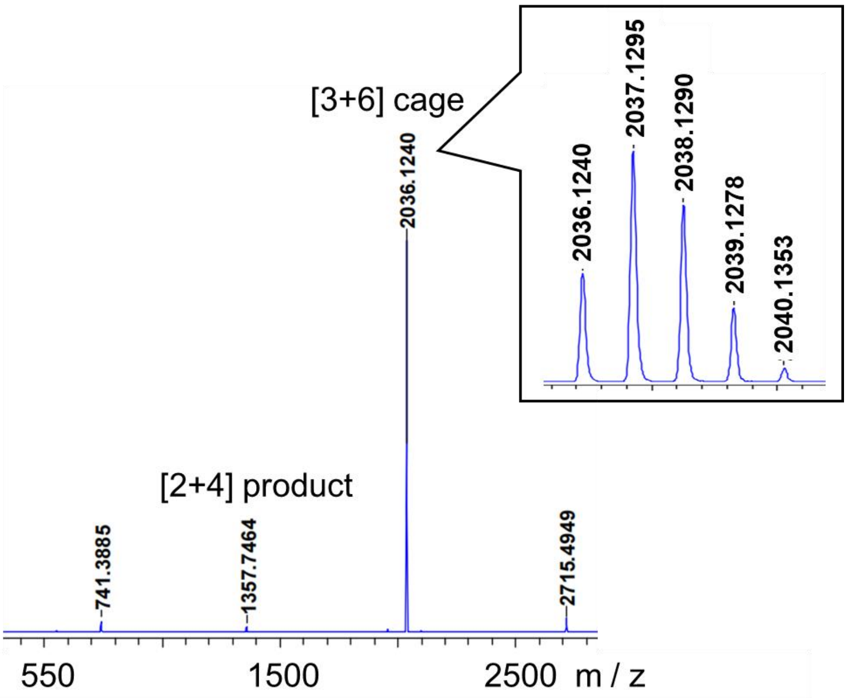

Supplementary Figure 25. MS characterization. High-resolution MALDI-TOF of the self-assembly product by condensing **0** and (*S,S*)-CHDA at 50 °C for 120 h.

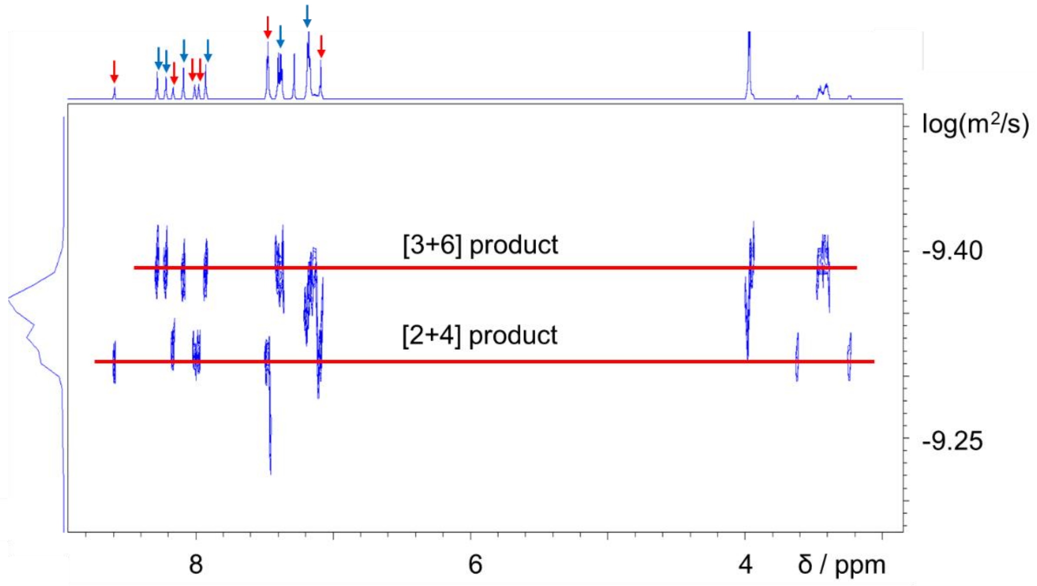

Supplementary Figure 26. NMR characterization. DOSY spectrum (600 MHz, $CDCl_3$, 298 K) of the mixed self-assembly products by condensing **0** and (*S,S*)-CHDA at 50 °C for 5 h.

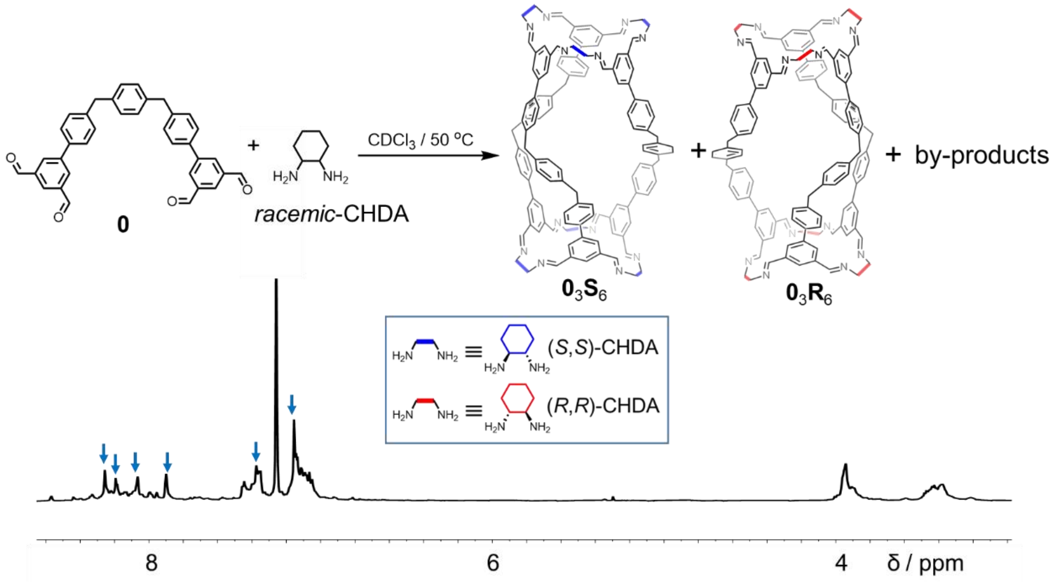

Supplementary Figure 27. NMR characterization. Partial ^1H NMR spectrum (400 MHz, $CDCl_3$, 298 K) of products by condensing **0** and racemic *trans*-CHDA in a 1:2 ratio at 50 °C for 24 h. The resonances of [3+6] cages are marked with blue arrows.

When we use racemic *trans*-CHDA instead of (*S,S*)-CHDA, the [3+6] chiral cage also becomes the major product. The corresponding 1H NMR spectrum showed more complicated resonances, indicating more byproducts were generated compared to the case with enantiomeric pure bisamine (Supplementary Figure 27).

4. Characterization of 1_3S_6

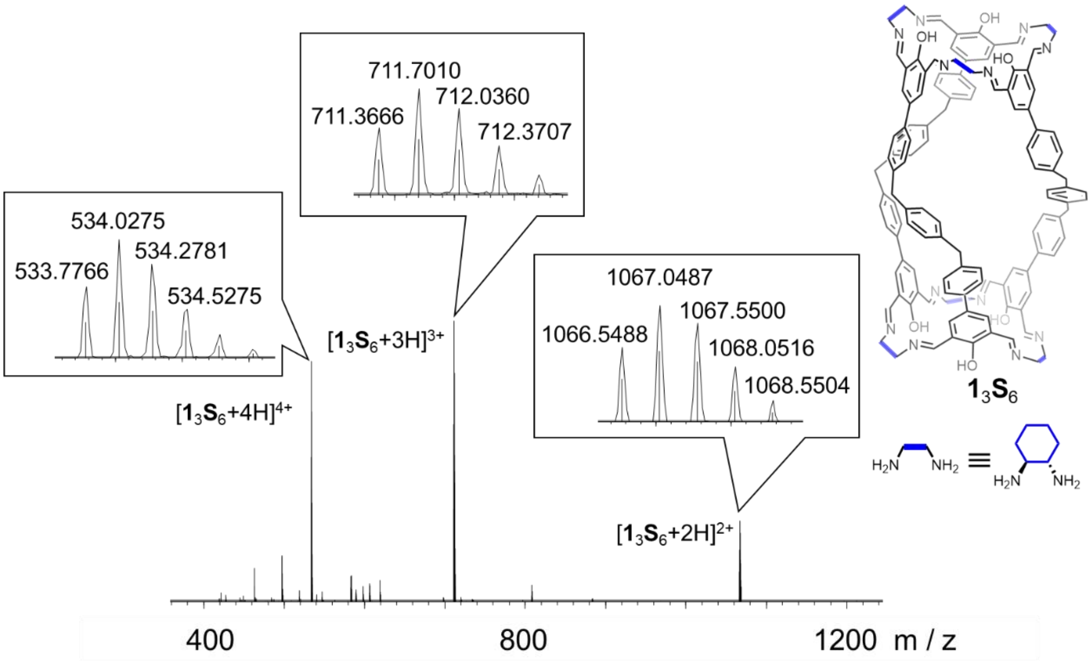

Supplementary Figure 28. High-resolution LCMS-IT-TOF of 1_3S_6. The signals labeled in the spectrum correspond to molecular cations that contain four, three, two positive charges, respectively. *m/z* [$\mathbf{1_3S_6}$ + 4H]$^{4+}$ calculated for $C_{144}H_{142}N_{12}O_6^{4+}$: 534.0297; found: 534.0275; [$\mathbf{1_3S_6}$ + 3H]$^{3+}$ calculated for $C_{144}H_{141}N_{12}O_6^{3+}$: 711.7038; found: 711.7010; [$\mathbf{1_3S_6}$ + 2H]$^{2+}$ calculated for $C_{144}H_{140}N_{12}O_6^{2+}$: 1067.0521; found: 1067.0487.

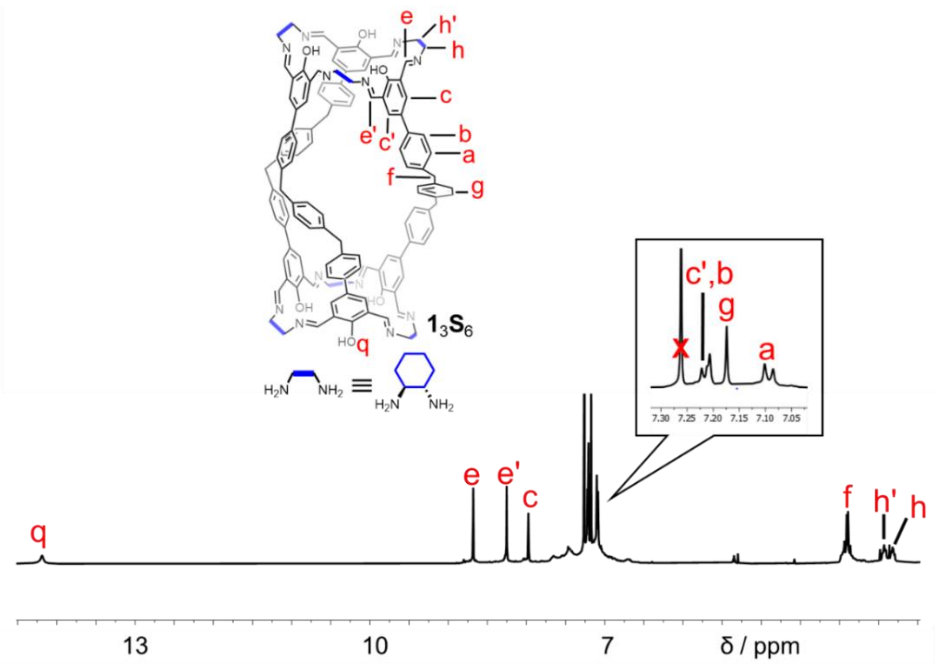

Supplementary Figure 29. NMR characterization. Partial ^{1}H NMR spectrum of $\mathbf{1_3S_6}$ (500 MHz, $CDCl_3$, 298 K).

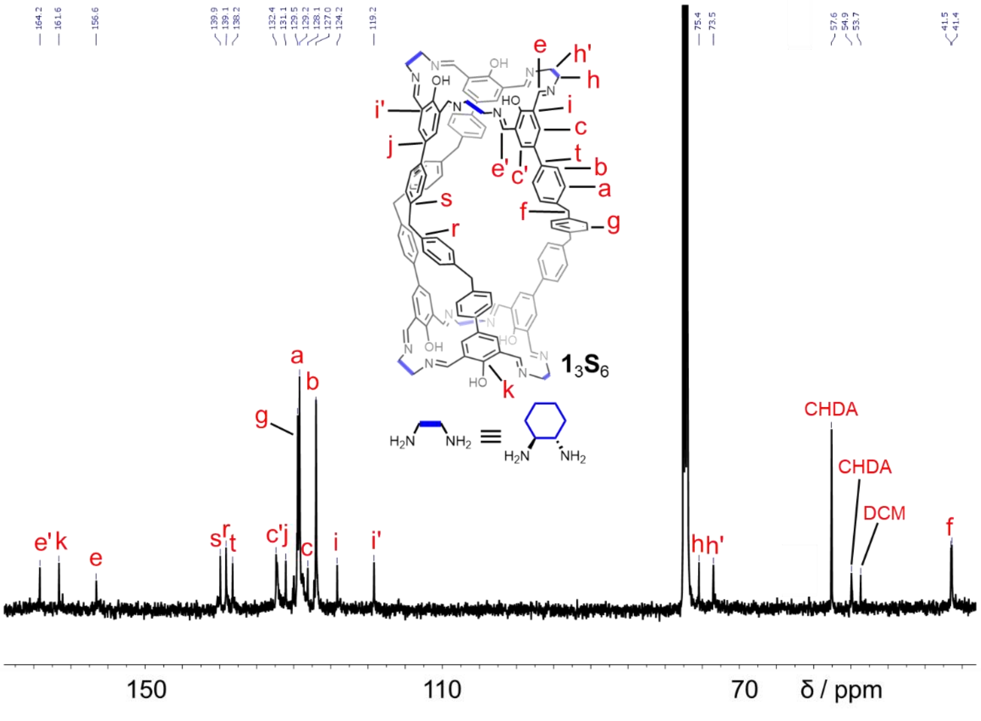

Supplementary Figure 30. NMR characterization. ^{13}C NMR spectrum (125 MHz, $CDCl_3$, 298 K) of $\mathbf{1_3S_6}$.

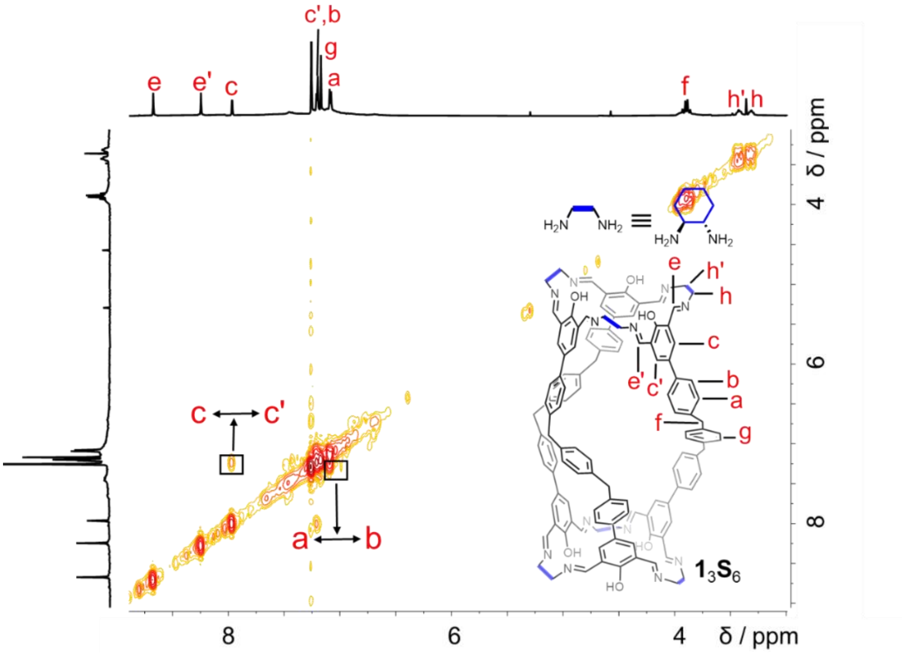

Supplementary Figure 31. NMR characterization. $^1H-^1H$ COSY spectrum (500 MHz, $CDCl_3$, 298 K) of $\mathbf{1_3S_6}$. Key correlation peaks are labeled in the spectrum.

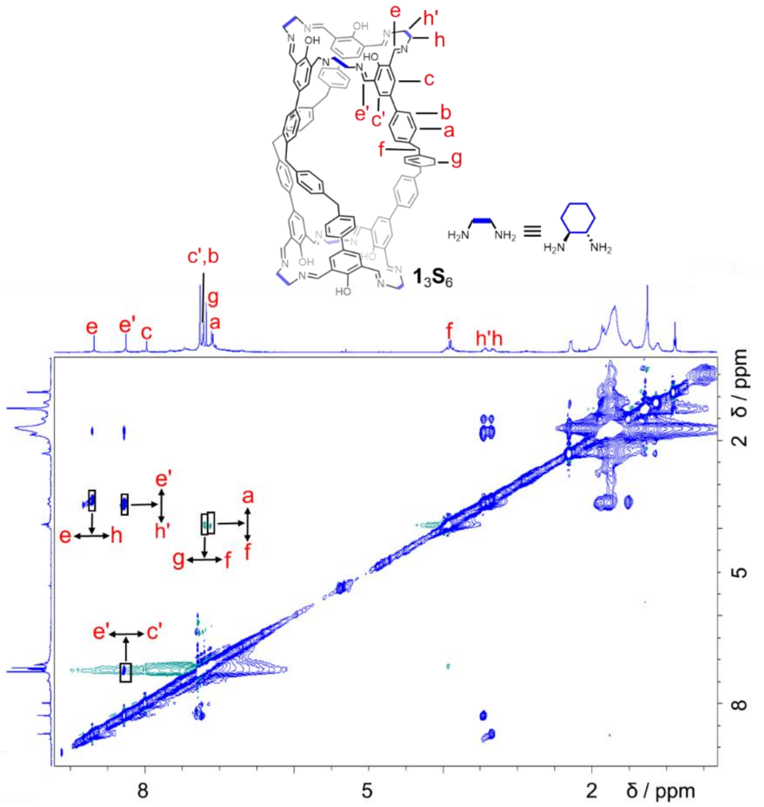

Supplementary Figure 32. NMR characterization. $^1H-^1H$ NOESY spectrum (500 MHz, $CDCl_3$, 298 K) of $\mathbf{1_3S_6}$. Key correlation peaks are labeled in the spectrum.

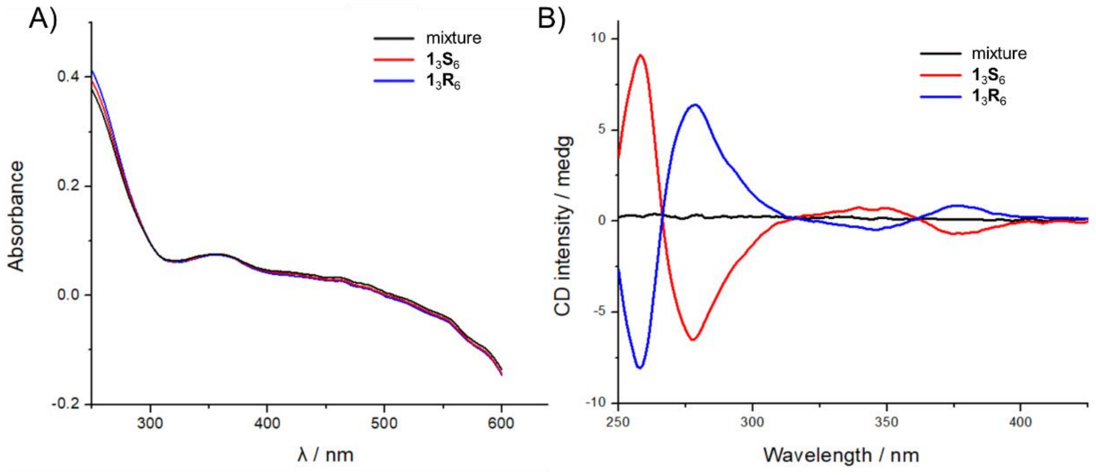

Supplementary Figure 33. UV-Vis absorption and CD spectra. A) UV-Vis absorption spectra and B) CD spectra of the products (0.06 mM in $CDCl_3$) including **1**$_3$**S**$_6$, **1**$_3$**R**$_6$ and the reaction mixture by condensing a 1:2 mixture of **1** and racemic *trans*-CHDA. The red, blue and black traces correspond to the cage **1**$_3$**S**$_6$, **1**$_3$**R**$_6$ and the reaction mixture of **1** and racemic *trans*-CHDA, respectively.

5. Characterization of $2_2R_2S_2$ and $2_2(EDA)_4$

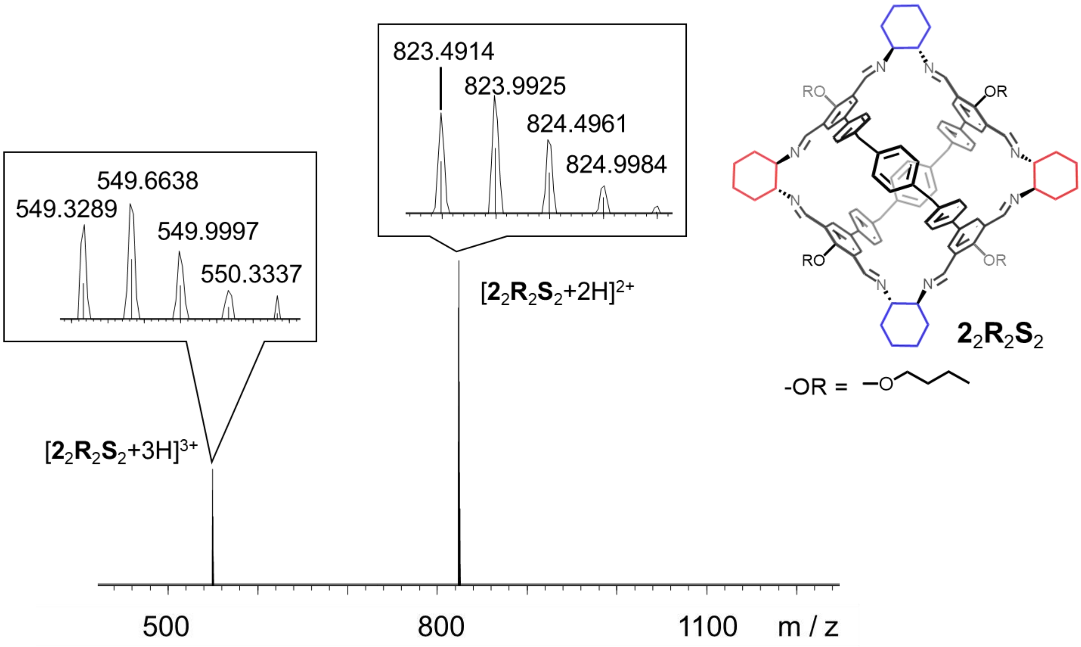

Supplementary Figure 34. High-resolution LCMS-IT-TOF of $\mathbf{2_2R_2S_2}$. The signals labeled in the spectrum correspond to molecular cations that contain three and two positive charges, respectively. *m/z* [$\mathbf{2_2R_2S_2}$ + 3H]$^{3+}$ calculated for $C_{112}H_{127}N_8O_4^{3+}$: 549.6666; found: 549.6638. [$\mathbf{2_2R_2S_2}$ + 2H]$^{2+}$ calculated for $C_{112}H_{126}N_8O_4^{2+}$: 823.9963; found: 823.9925.

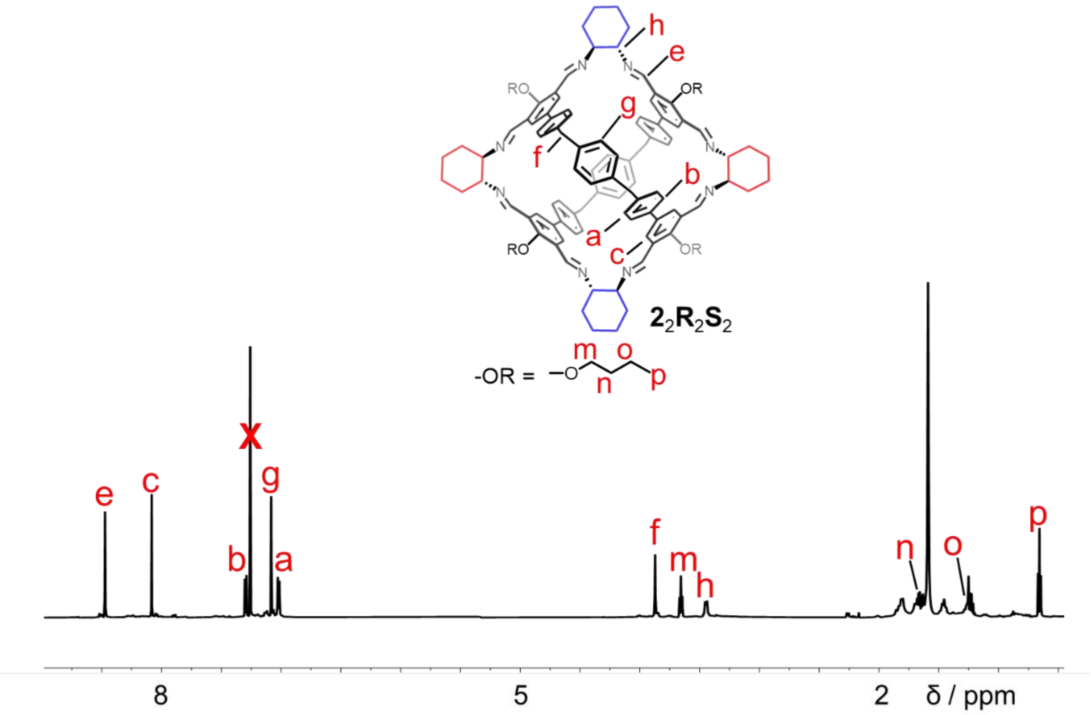

Supplementary Figure 35. NMR characterization. Partial ^{1}H NMR spectrum (500 MHz, $CDCl_3$, 298 K) of $\mathbf{2_2R_2S_2}$.

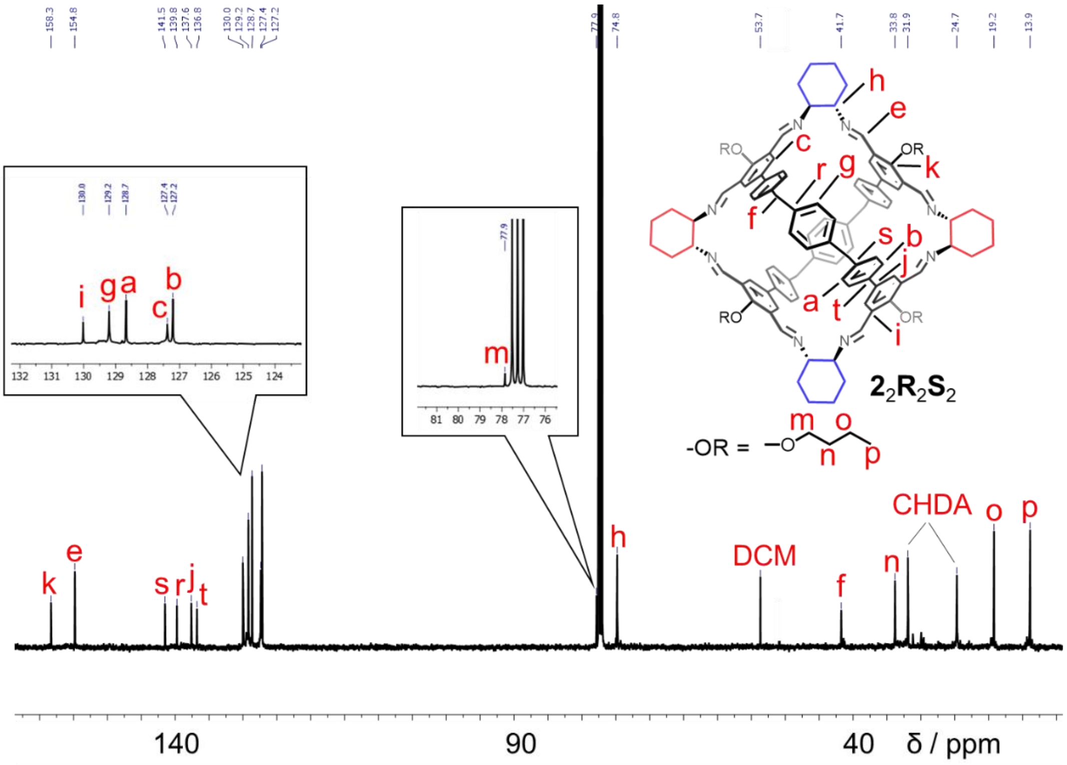

Supplementary Figure 36. NMR characterization. ^{13}C NMR spectrum (125 MHz, $CDCl_3$, 298 K) of $\mathbf{2_2R_2S_2}$.

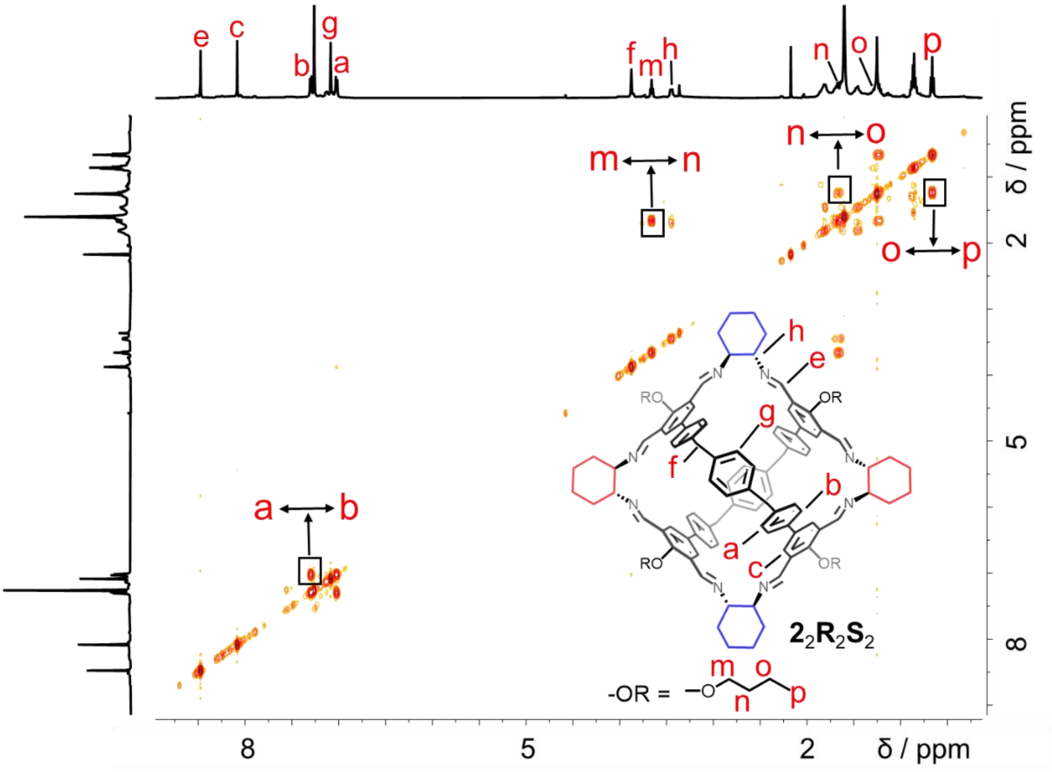

Supplementary Figure 37. NMR characterization. $^1H–^1H$ COSY spectrum (500 MHz, $CDCl_3$, 298 K) of $\mathbf{2_2R_2S_2}$. Key correlation peaks are labeled in the spectrum.

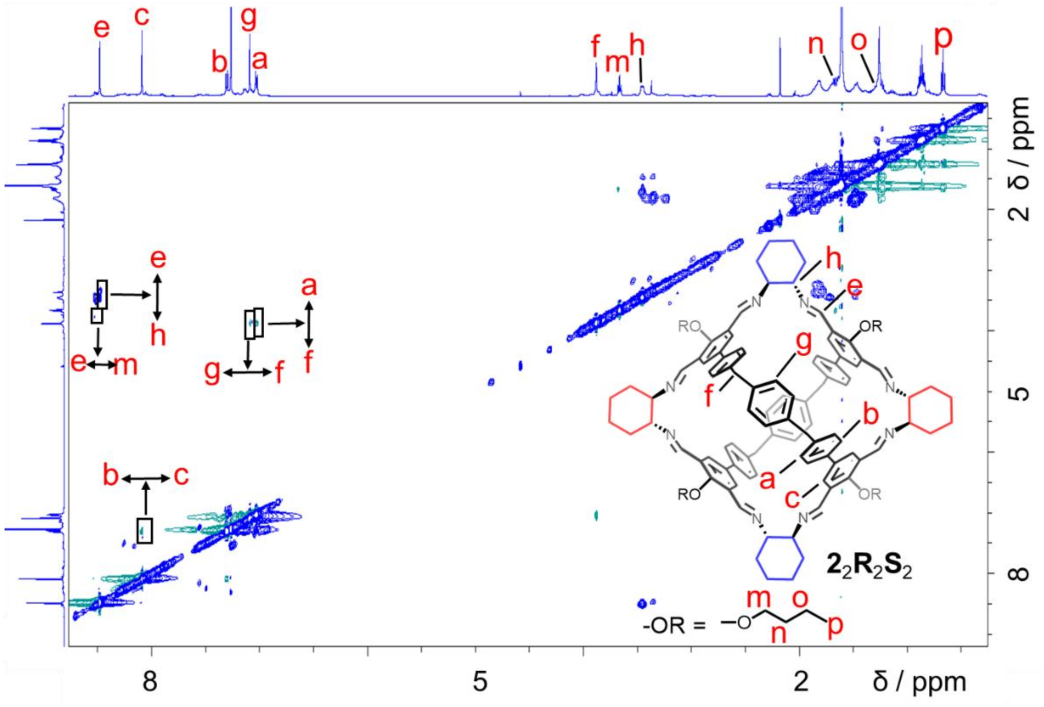

Supplementary Figure 38. NMR characterization. $^1H–^1H$ NOESY spectrum (500 MHz, $CDCl_3$, 298 K) of $\mathbf{2_2R_2S_2}$. Key correlation peaks are labeled in the spectrum.

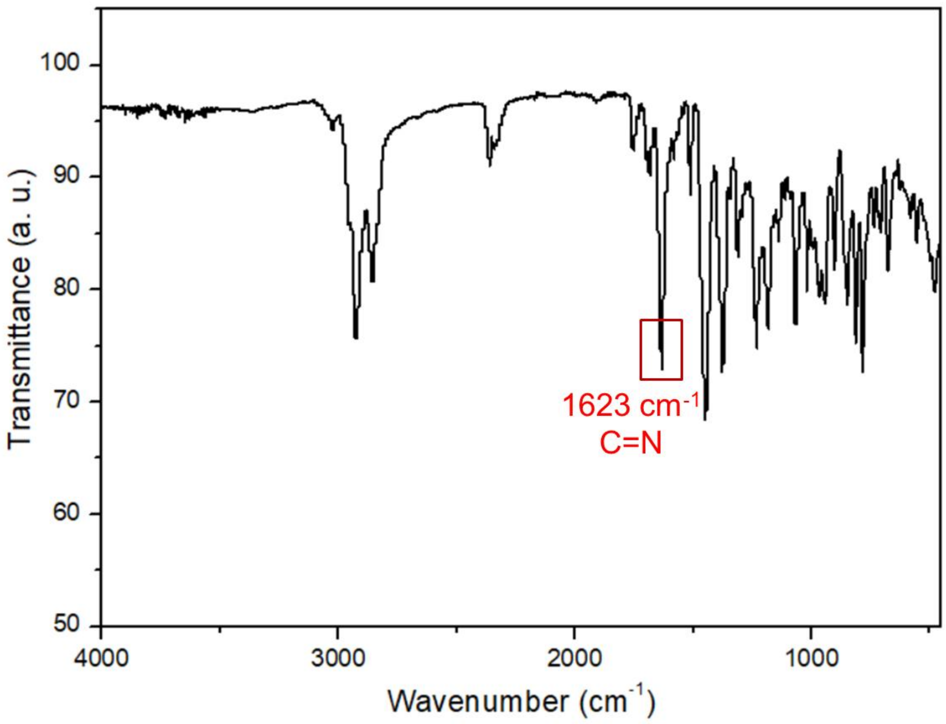

Supplementary Figure 39. FT-IR characterization. FT-IR spectrum of $\mathbf{2_2R_2S_2}$.

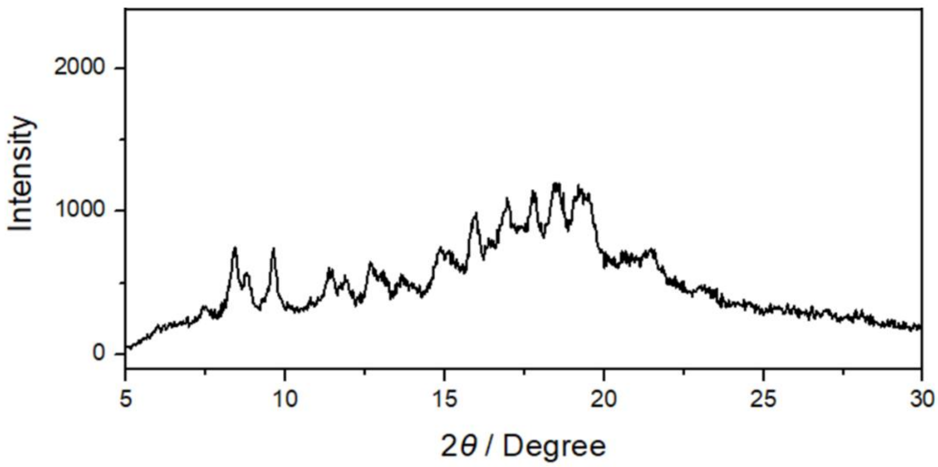

Supplementary Figure 40. PXRD characterization. PXRD pattern of.$\mathbf{2_2R_2S_2}$.

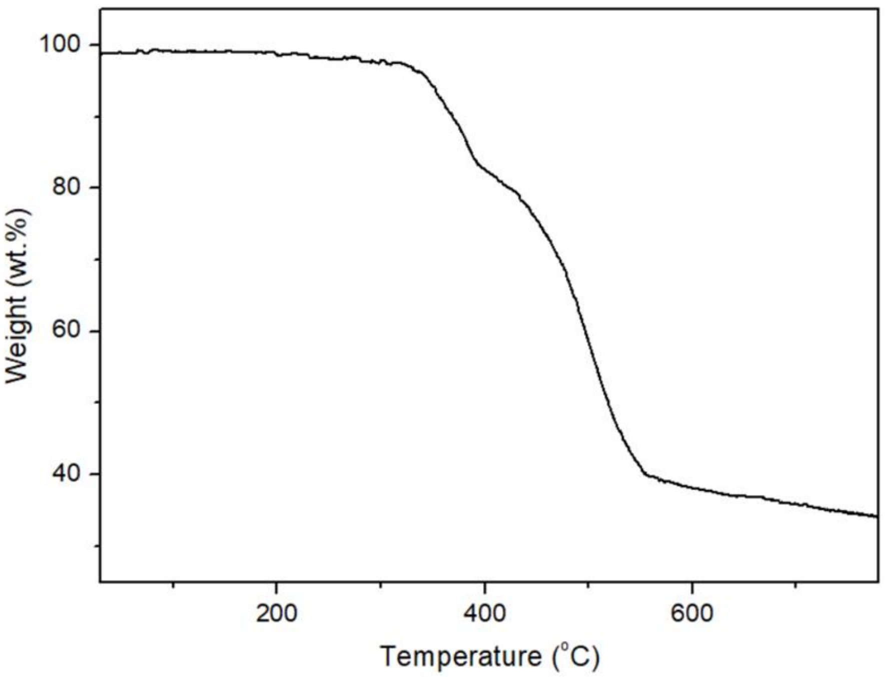

Supplementary Figure 41. TGA characterization. TGA of.$\mathbf{2_2R_2S_2}$ solid.

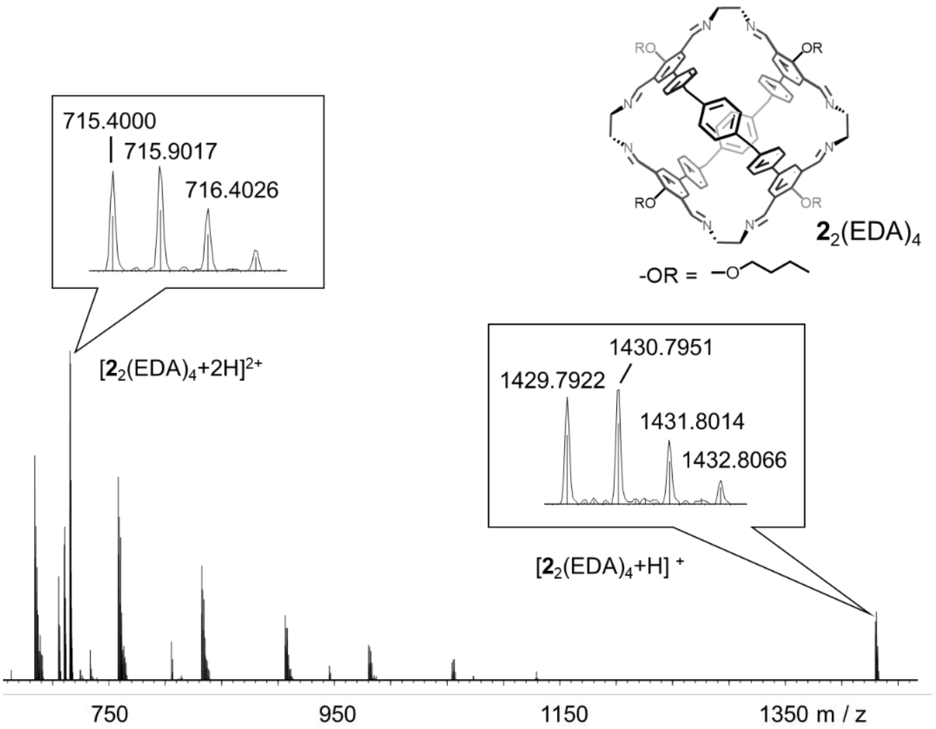

Supplementary Figure 42. High-resolution LCMS-IT-TOF of $\mathbf{2_2}$(EDA)$_4$. The signal labeled in the spectrum correspond to molecular cations that contains two or one positive charges. *m/z* [**2**$_2$(EDA)$_4$+2H]$^{2+}$calculated for $C_{96}H_{102}N_8O_4{}^{2+}$: 715.9024; found: 715.9017. [**2**$_2$(EDA)$_4$+H]$^+$ calculated for $C_{96}H_{101}N_8O_4{}^{2+}$: 1430.7974; found: 1430.7951.

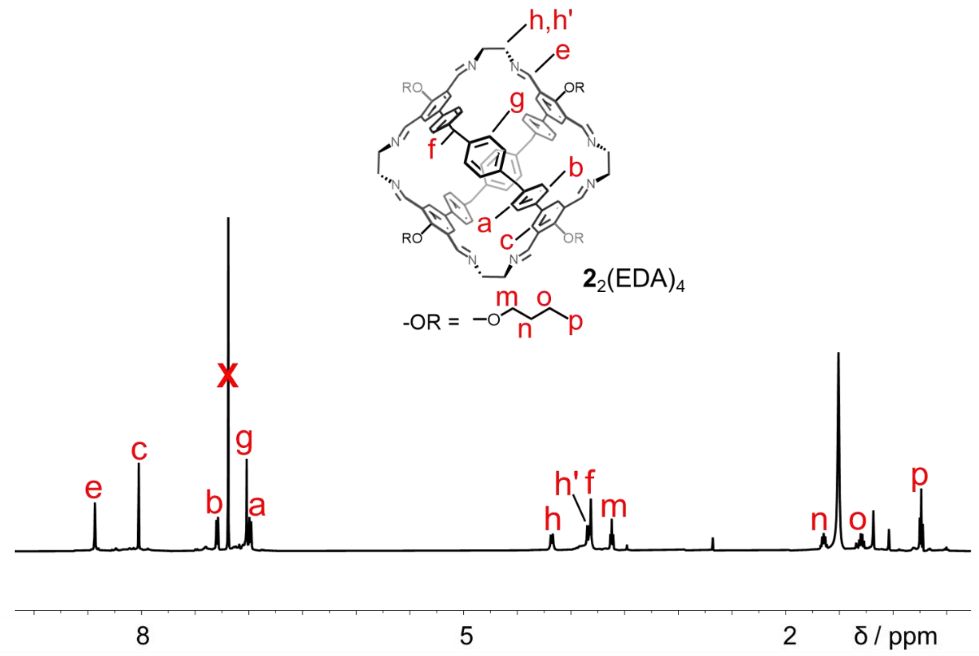

Supplementary Figure 43. NMR characterization. Partial ^{1}H NMR spectrum of **2**$_2$(EDA)$_4$ (500 MHz, $CDCl_3$, 298 K).

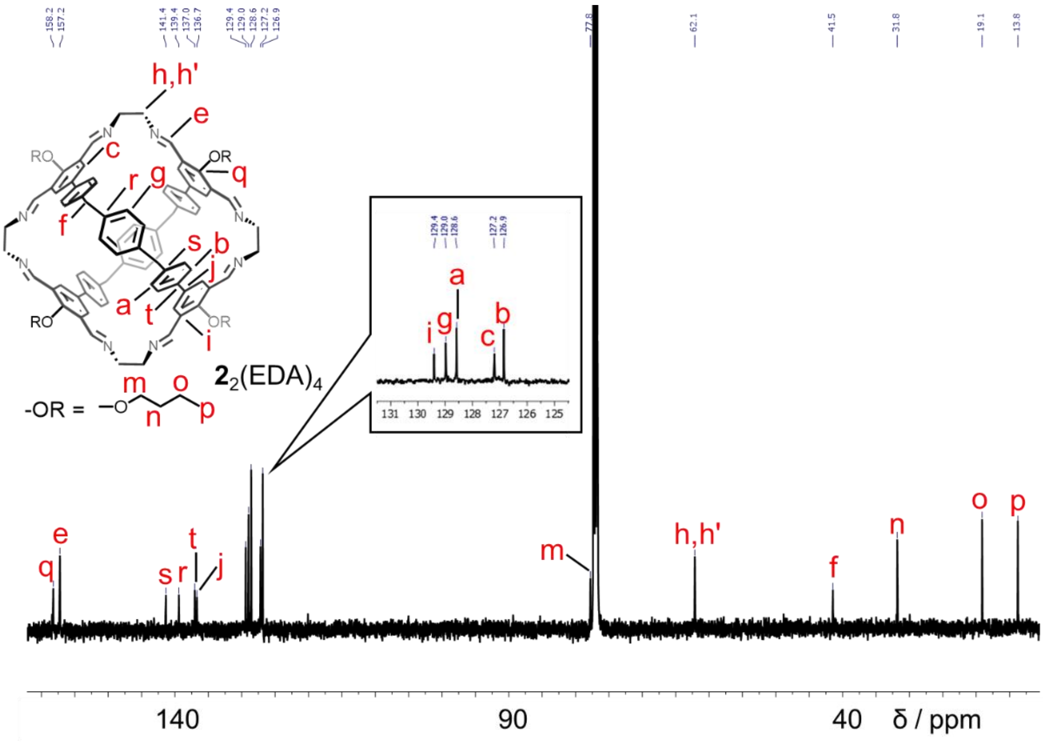

Supplementary Figure 44. NMR characterization. ^{13}C NMR spectrum (125 MHz, $CDCl_3$, 298 K) of **2**$_2$(EDA)$_4$.

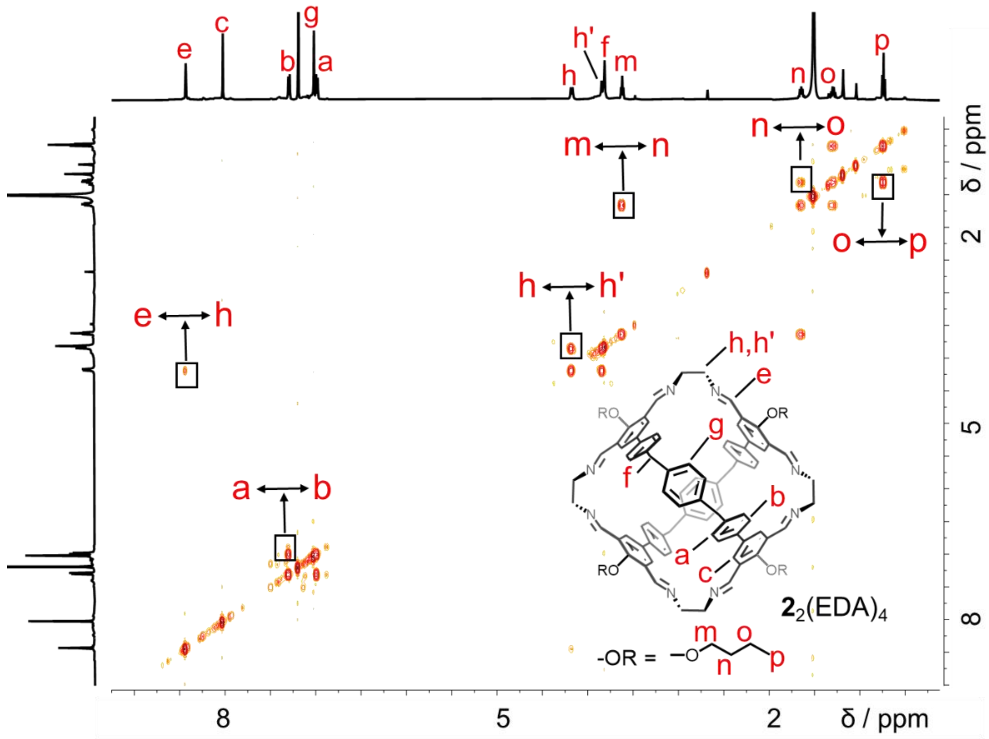

Supplementary Figure 45. NMR characterization. ^{1}H−^{1}H COSY spectrum (500 MHz, $CDCl_3$, 298 K) of **2**$_2$(EDA)$_4$. Key correlation peaks are labeled in the spectrum.

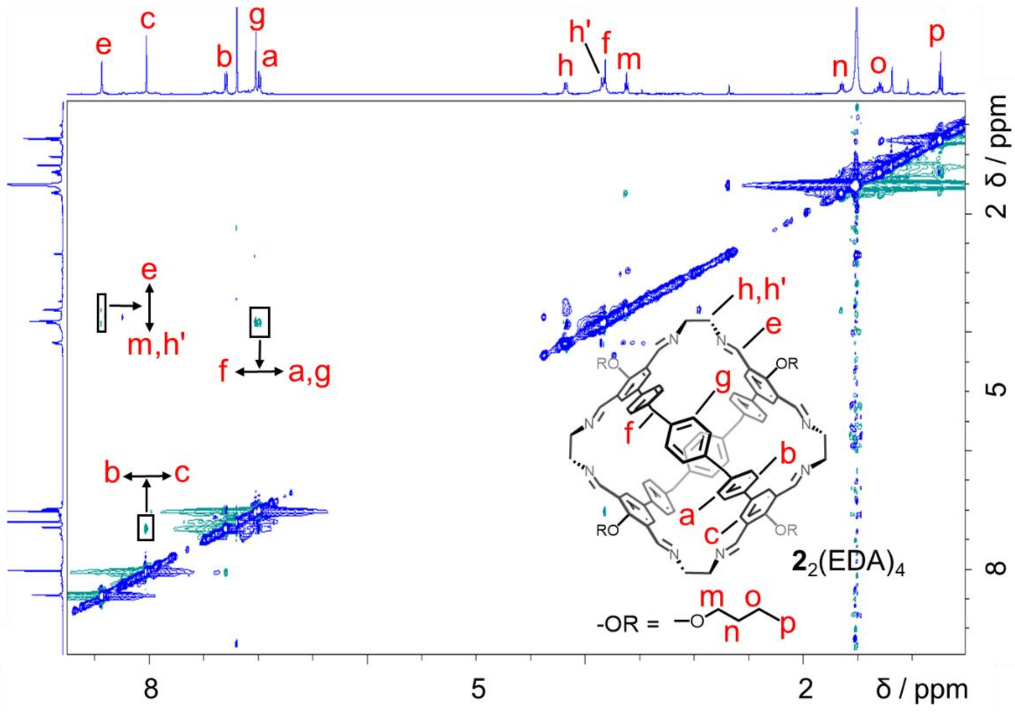

Supplementary Figure 46. NMR characterization. ^{1}H−^{1}H NOESY spectrum (500 MHz, $CDCl_3$, 298 K) of **2**$_2$(EDA)$_4$. Key correlation peaks are labeled in the spectrum.

6. Characterization of $3_2R_2S_2$, $3_2(EDA)_4$ and 3_3S_6

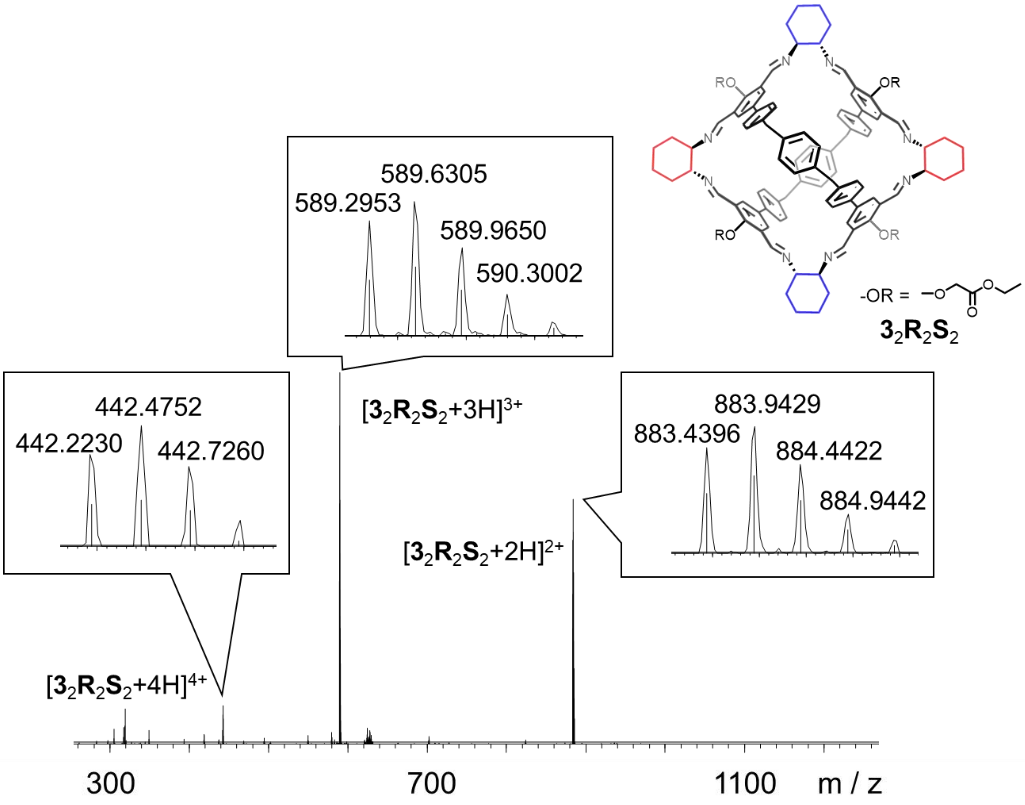

Supplementary Figure 47. High-resolution LCMS-IT-TOF of $\mathbf{3_2R_2S_2}$. The signals labeled in the spectrum correspond to molecular cations that contain four, three and two positive charges, respectively. *m/z* [$\mathbf{3_2R_2S_2}$ + 4H]$^{4+}$ calculated for $C_{112}H_{120}N_8O_{12}{}^{4+}$: 442.4759; found: 442.4752. [$\mathbf{3_2R_2S_2}$ + 3H]$^{3+}$ calculated for $C_{112}H_{119}N_8O_{12}{}^{3+}$: 589.6322; found: 589.6305. [$\mathbf{3_2R_2S_2}$ + 2H]$^{2+}$ calculated for $C_{112}H_{118}N_8O_{12}{}^{2+}$: 883.9446; found: 883.9429.

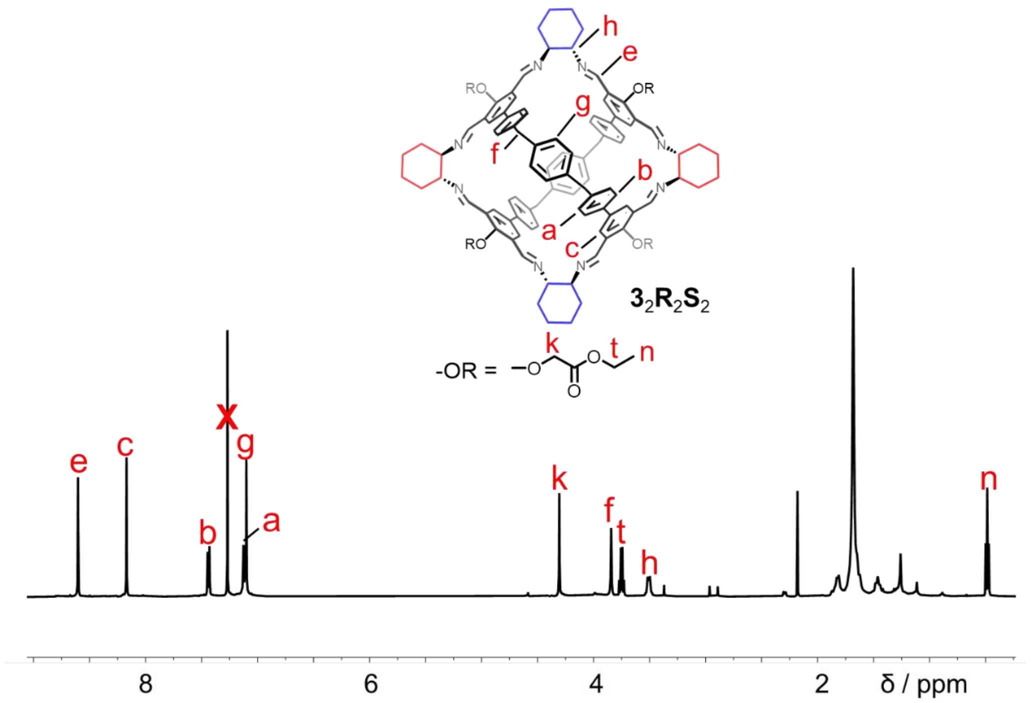

Supplementary Figure 48. NMR characterization. Partial ^{1}H NMR spectrum of $\mathbf{3_2R_2S_2}$ (500 MHz, $CDCl_3$, 298 K).

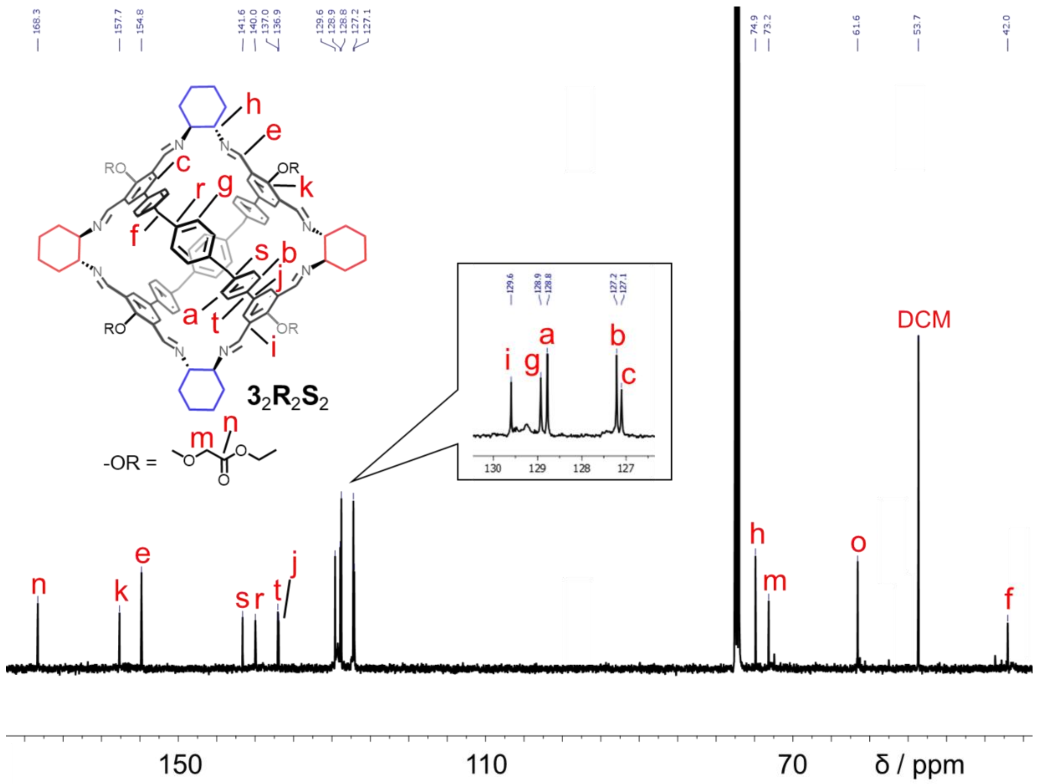

Supplementary Figure 49. NMR characterization. ^{13}C NMR spectrum (125 MHz, $CDCl_3$, 298 K) of $\mathbf{3_2R_2S_2}$.

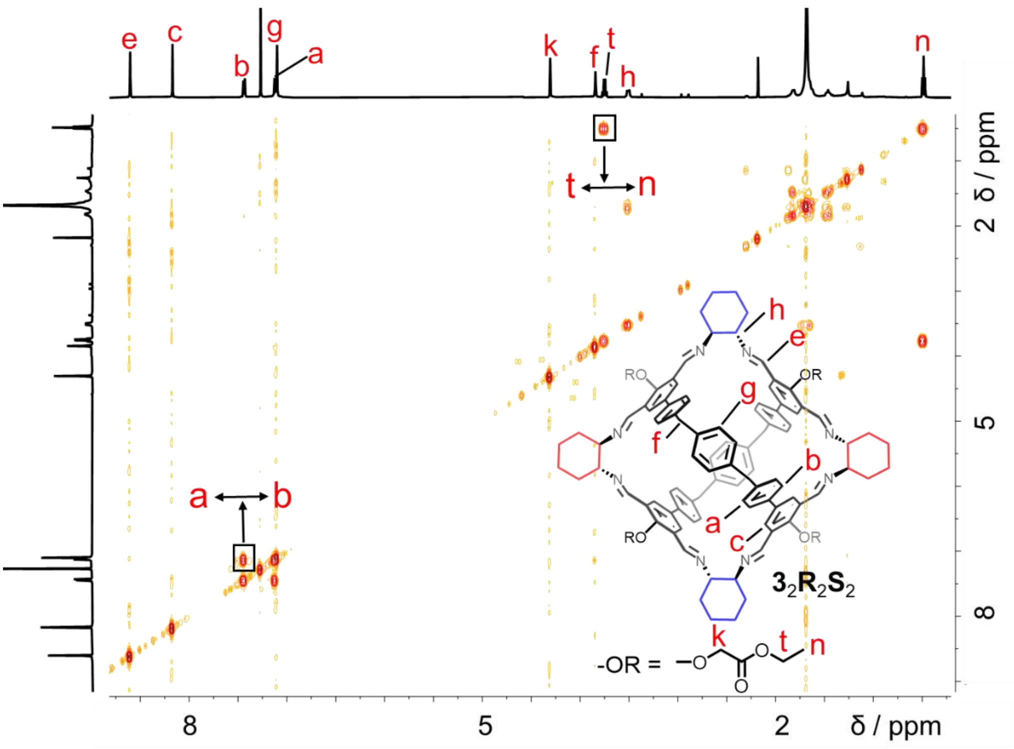

Supplementary Figure 50. NMR characterization. ^1H–^1H COSY spectrum (500 MHz, $CDCl_3$, 298 K) of $\mathbf{3_2R_2S_2}$. Key correlation peaks are labeled in the spectrum.

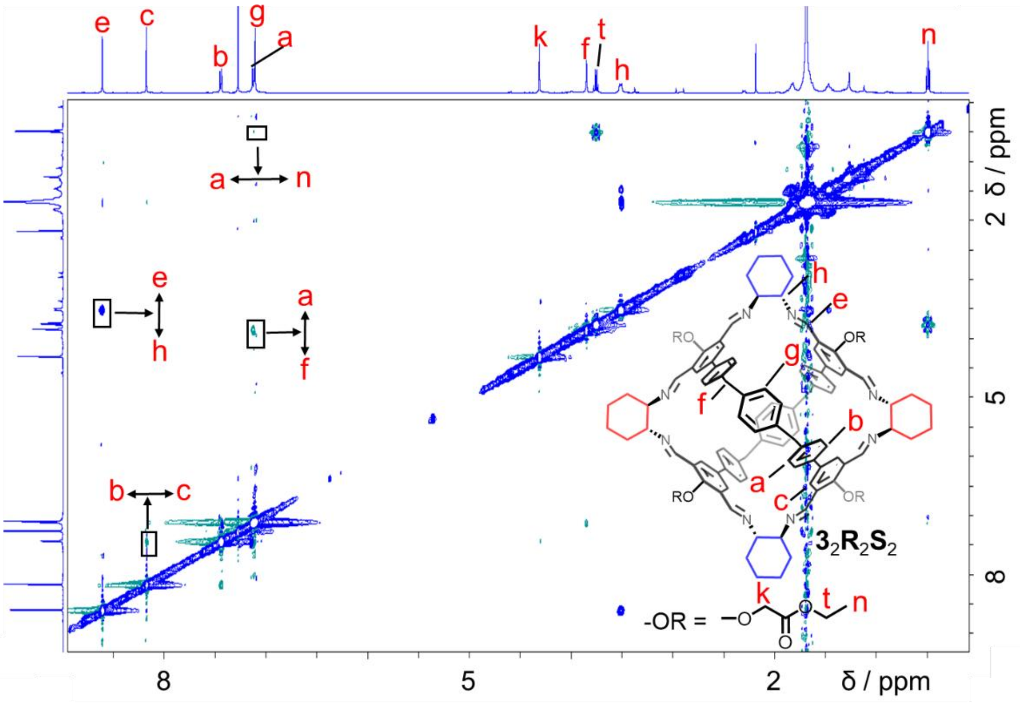

Supplementary Figure 51. NMR characterization. ^1H–^1H NOESY spectrum (500 MHz, $CDCl_3$, 298 K) of $\mathbf{3_2R_2S_2}$. Key correlation peaks are labeled in the spectrum.

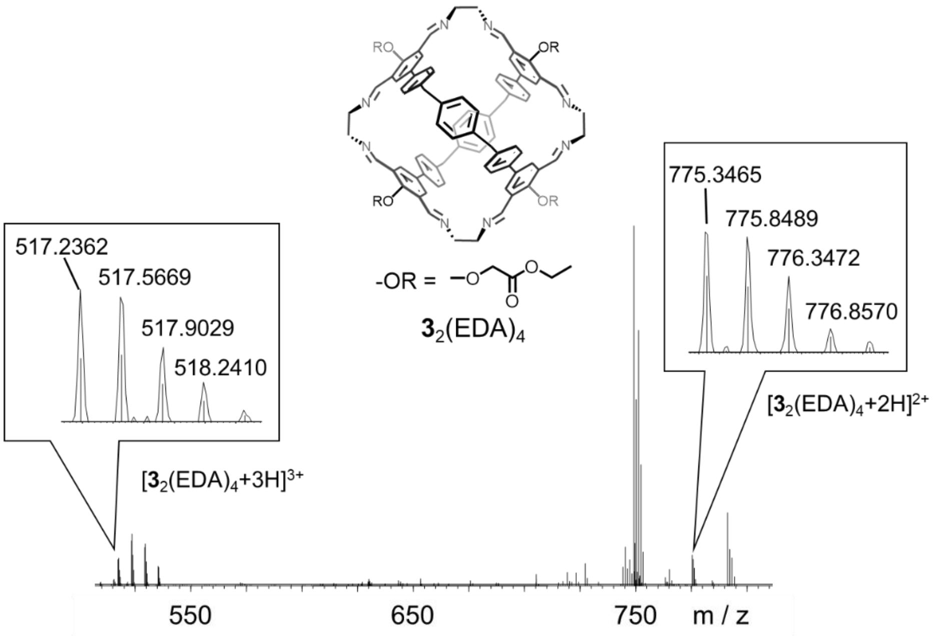

Supplementary Figure 52. High-resolution LCMS-IT-TOF of $\mathbf{3_2(EDA)_4}$. The signals labeled in the spectrum correspond to molecular cations that contain three and two positive charges, respectively. *m/z* $[\mathbf{3}_2(EDA)_4+3H]^{3+}$ calculated for $C_{96}H_{95}N_8O_{12}^{3+}$: 517.5696; found: 517.5669. $[\mathbf{3}_2(EDA)_4+2H]^{2+}$ calculated for $C_{96}H_{94}N_8O_{12}^{2+}$: 775.8507; found: 775.8489.

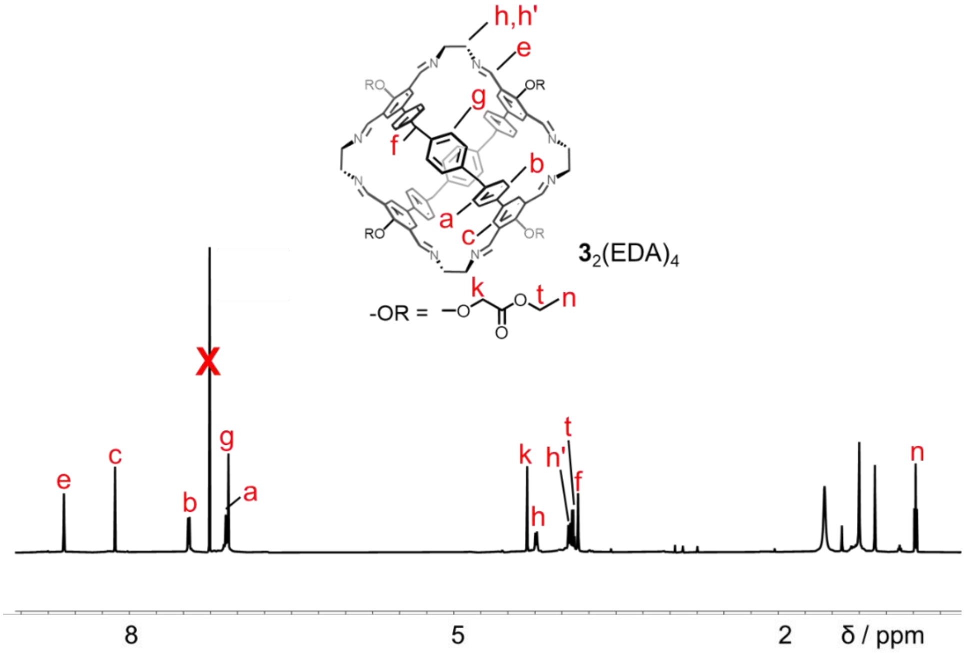

Supplementary Figure 53. NMR characterization. Partial ^1H NMR spectrum of $\mathbf{3}_2(EDA)_4$ (500 MHz, $CDCl_3$, 298 K).

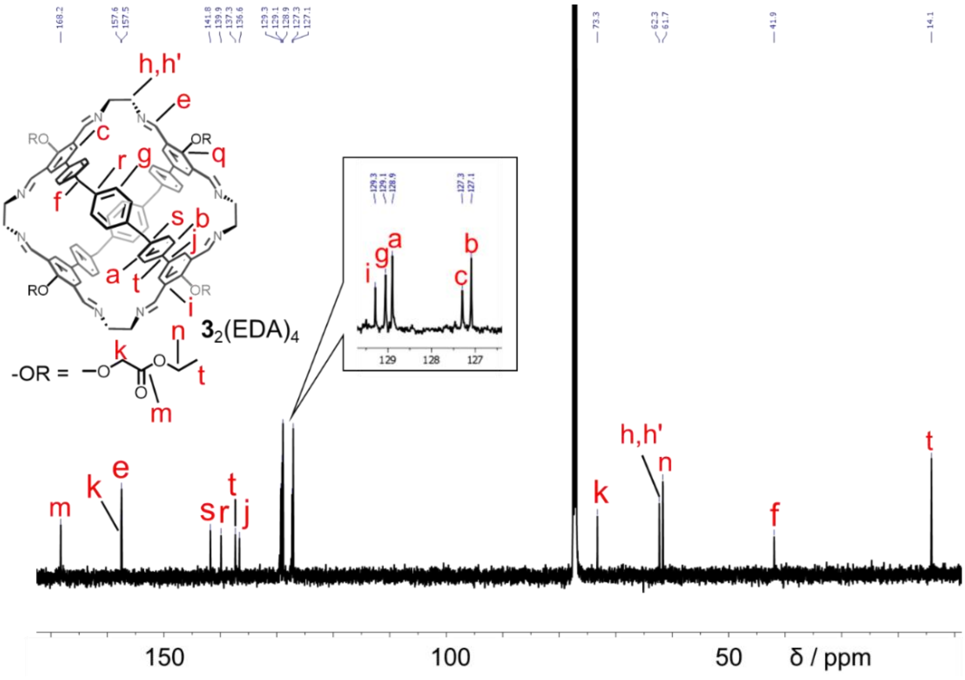

Supplementary Figure 54. NMR characterization. ^{13}C NMR spectrum (125 MHz, $CDCl_3$, 298 K) of $\mathbf{3}_2$(EDA)$_4$.

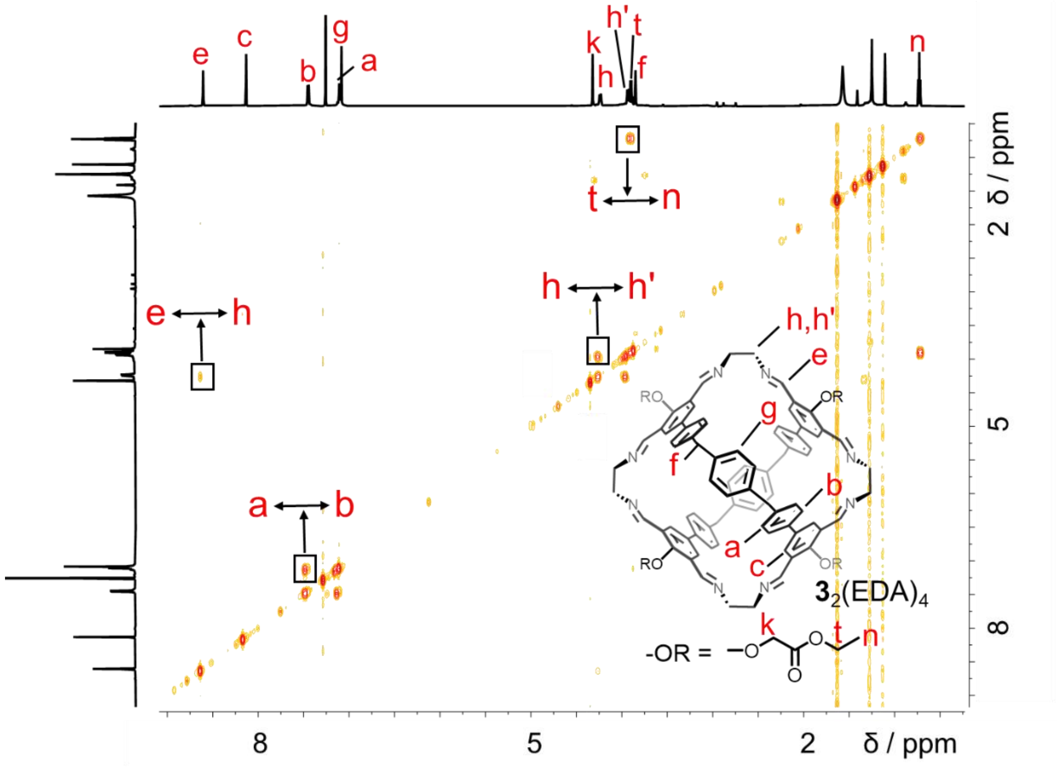

Supplementary Figure 55. NMR characterization. $^1H-^1H$ COSY spectrum (500 MHz, $CDCl_3$, 298 K) of $\mathbf{3}_2$(EDA)$_4$. Key correlation peaks are labeled in the spectrum.

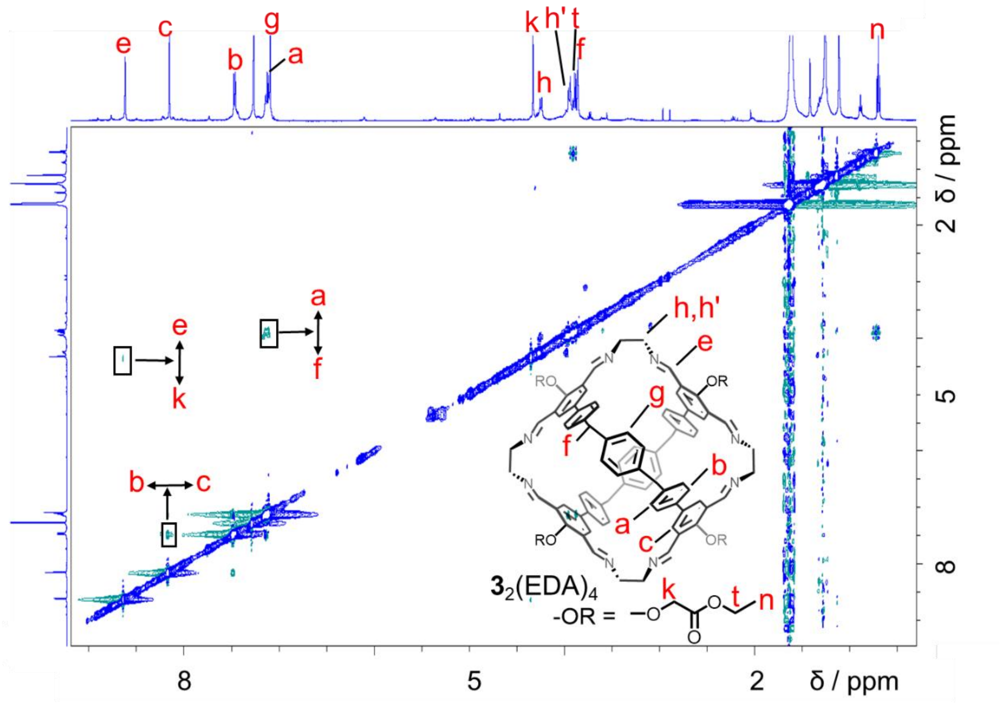

Supplementary Figure 56. NMR characterization. $^1H-^1H$ NOESY spectrum (500 MHz, $CDCl_3$, 298 K) of **3**$_2$(EDA)$_4$. Key correlation peaks are labeled in the spectrum.

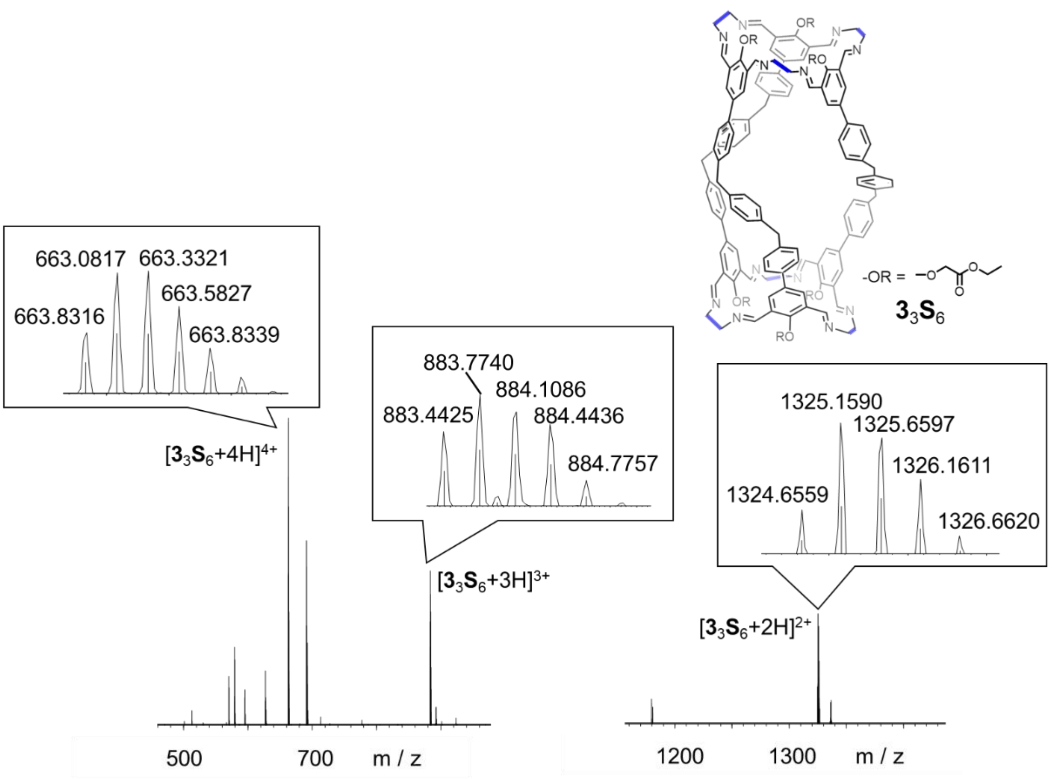

Supplementary Figure 57. High-resolution LCMS-IT-TOF of 3_3S_6. The signals labeled in the spectrum correspond to molecular cations that contain four, three and two positive charges, respectively. *m/z* [$\mathbf{3_3S_6}$ + 4H]$^{4+}$ calculated for $C_{168}H_{178}N_{12}O_{18}^{4+}$: 663.0849; found: 663.0817. [$\mathbf{3_3S_6}$ + 3H]$^{3+}$ calculated for $C_{168}H_{177}N_{12}O_{18}^{3+}$: 883.7774; found: 883.7740. [$\mathbf{3_3S_6}$ + 2H]$^{2+}$ calculated for $C_{168}H_{176}N_{12}O_{18}^{2+}$: 1325.1624; found: 1325.1590.

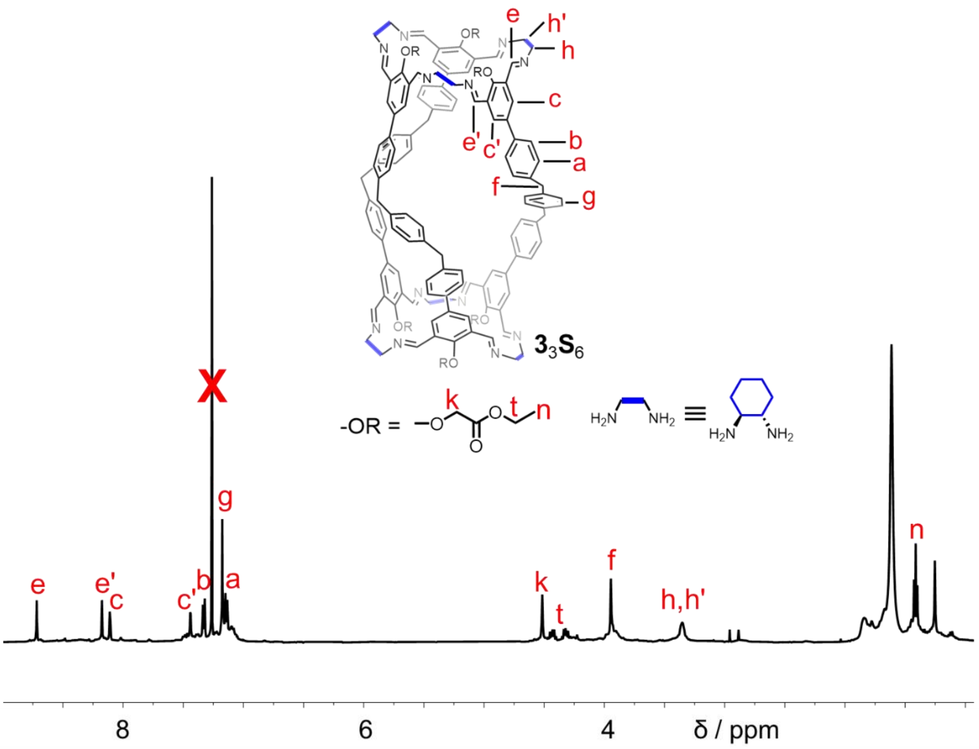

Supplementary Figure 58. NMR characterization. Partial ^{1}H NMR spectrum of $\mathbf{3_3S_6}$ (500 MHz, $CDCl_3$, 298 K).

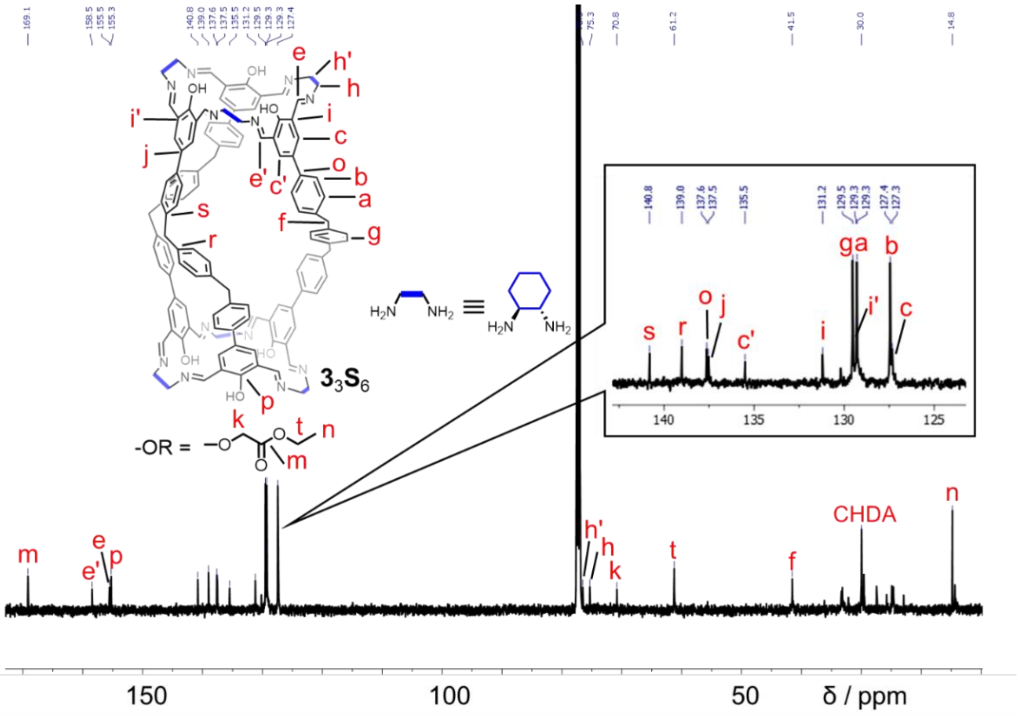

Supplementary Figure 59. NMR characterization. ^{13}C NMR spectrum (125 MHz, $CDCl_3$, 298 K) of $\mathbf{3_3S_6}$.

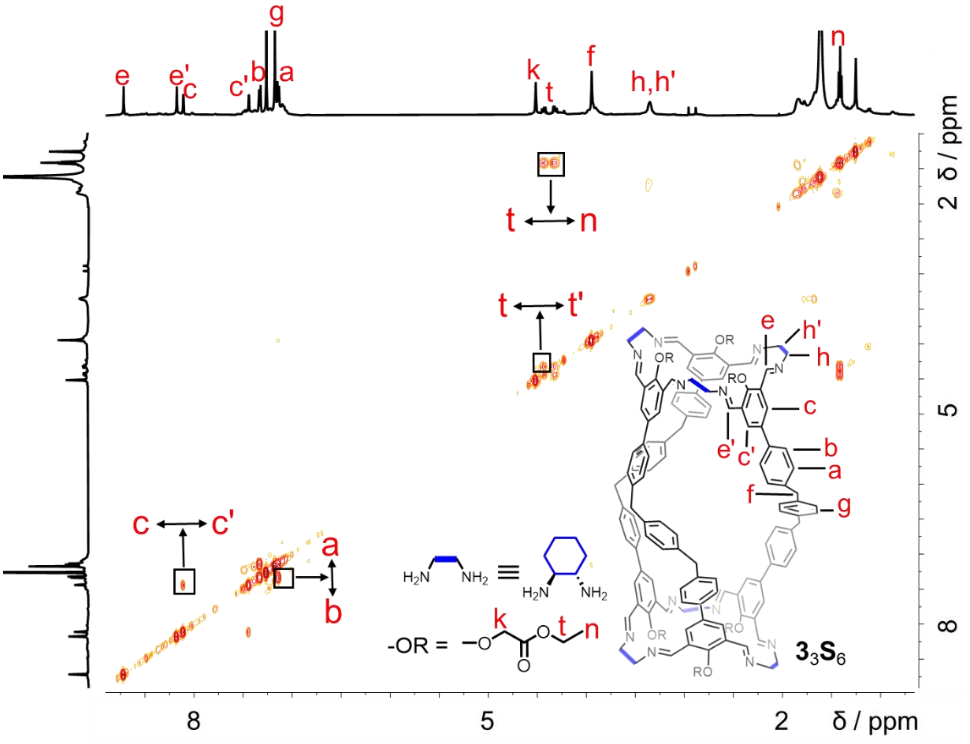

Supplementary Figure 60. NMR characterization. $^1H-^1H$ COSY spectrum (500 MHz, $CDCl_3$, 298 K) of $\mathbf{3_3S_6}$. Key correlation peaks are labeled in the spectrum.

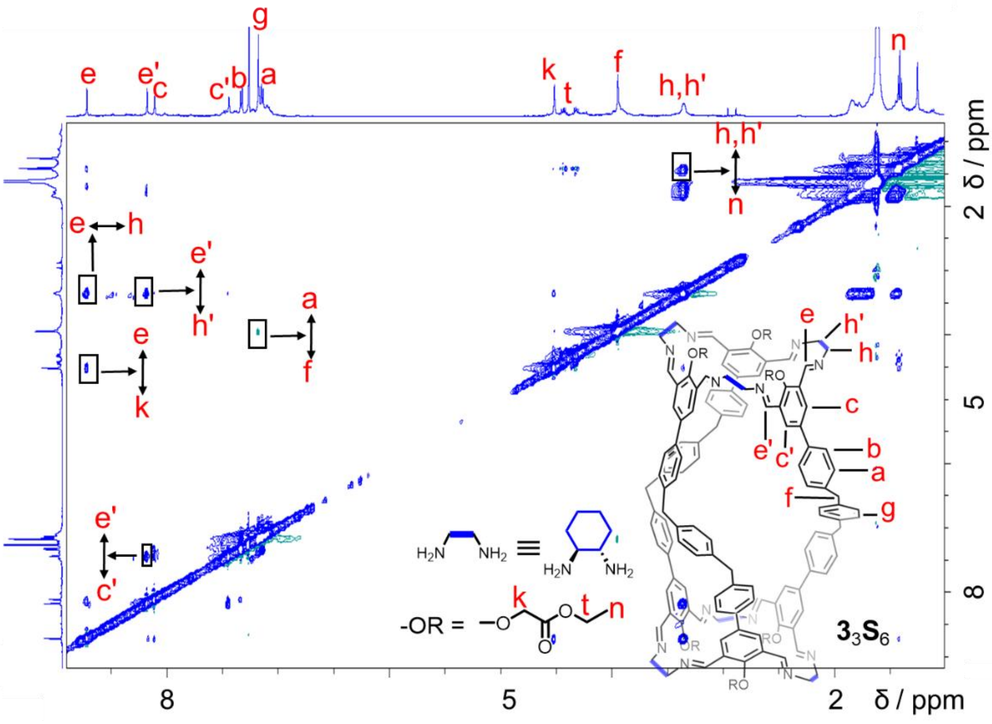

Supplementary Figure 61. NMR characterization. $^1H-^1H$ NOESY spectrum (500 MHz, $CDCl_3$, 298 K) of $\mathbf{3_3S_6}$. Key correlation peaks are labeled in the spectrum.

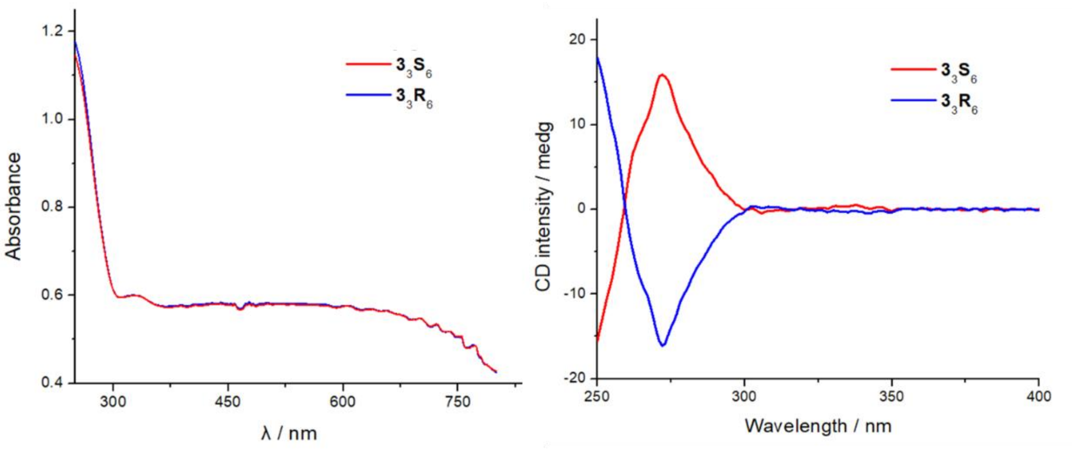

Supplementary Figure 62. UV-Vis absorption and CD spectra. A) UV-Vis absorption spectra and B) CD spectra of the reaction mixture by condensing **3** and the chiral bisamine namely either (*S*,*S*)-CHDA or (*R*,*R*)-CHDA, respectively, which forms either $\mathbf{3_3S_6}$ or $\mathbf{3_3R_6}$ (0.03 mM in $CDCl_3$). The red and blue traces correspond to the cage $\mathbf{3_3S_6}$ and $\mathbf{3_3R_6}$, respectively.

7. Characterization of $4_2R_2S_2$ and $4_2(EDA)_4$

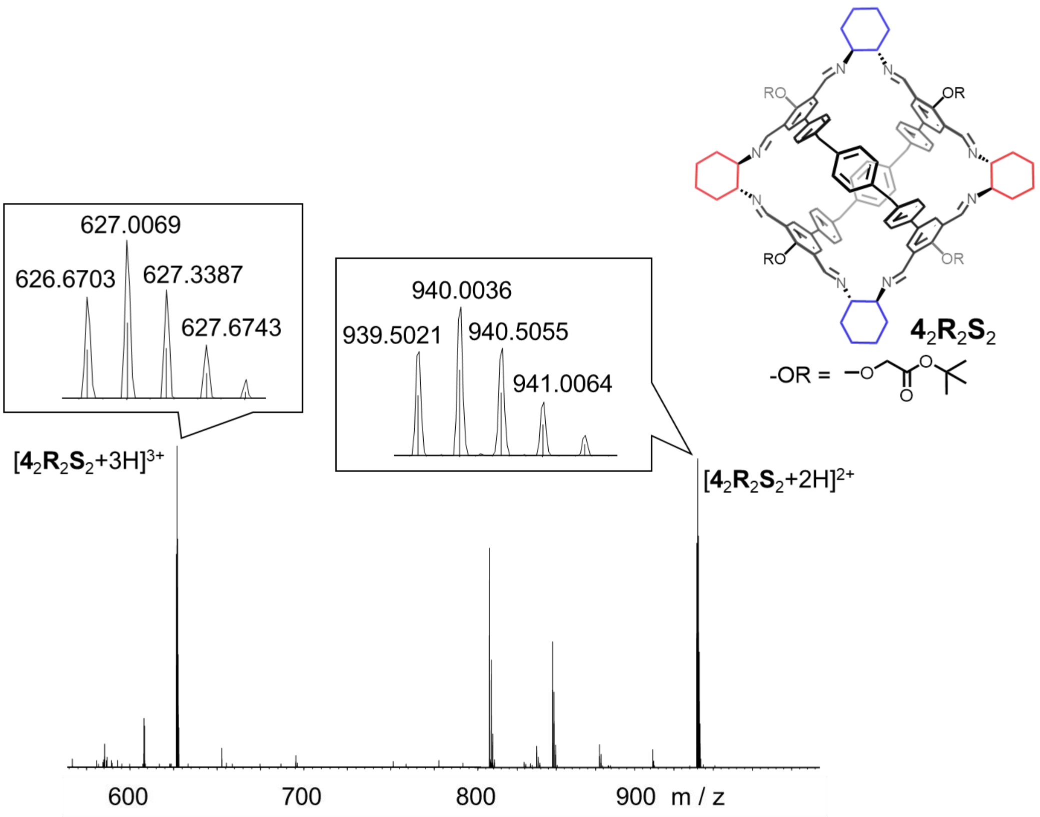

Supplementary Figure 63. High-resolution LCMS-IT-TOF of $4_2R_2S_2$. The signals labeled in the spectrum correspond to molecular cations that contain three and two positive charges, respectively. *m/z* [$\mathbf{4}_2\mathbf{R}_2\mathbf{S}_2$ + 3H]$^{3+}$ calculated for $C_{120}H_{135}N_8O_{12}^{3+}$: 627.0072; found: 627.0069. [$\mathbf{4}_2\mathbf{R}_2\mathbf{S}_2$ + 2H]$^{2+}$ calculated for $C_{120}H_{134}N_8O_{12}^{2+}$: 940.0072; found: 940.0036.

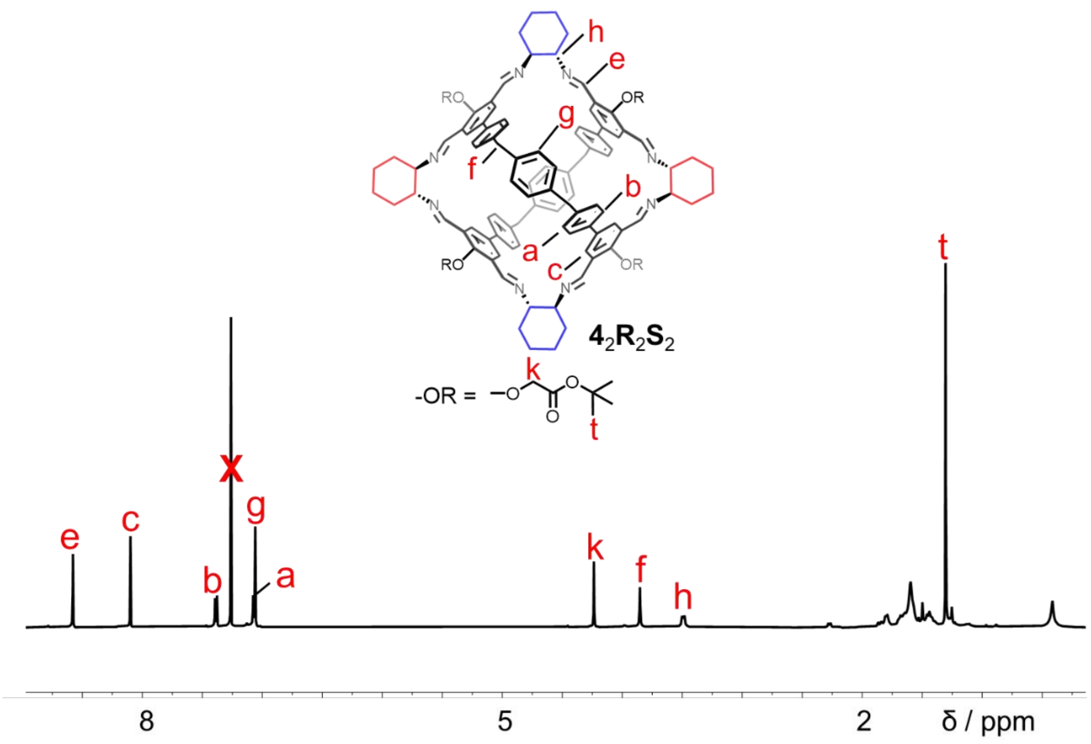

Supplementary Figure 64. NMR characterization. Partial ^{1}H NMR spectrum of $\mathbf{4_2R_2S_2}$ (500 MHz, $CDCl_3$, 298 K).

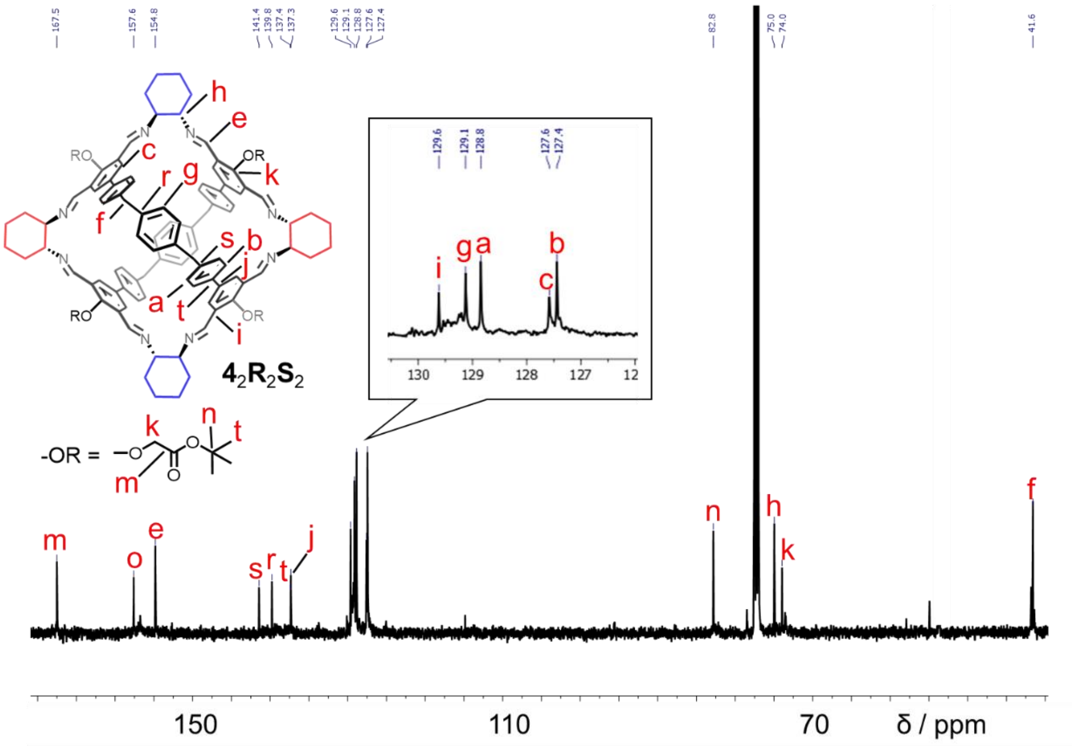

Supplementary Figure 65. NMR characterization. ^{13}C NMR spectrum (125 MHz, $CDCl_3$, 298 K) of $\mathbf{4_2R_2S_2}$.

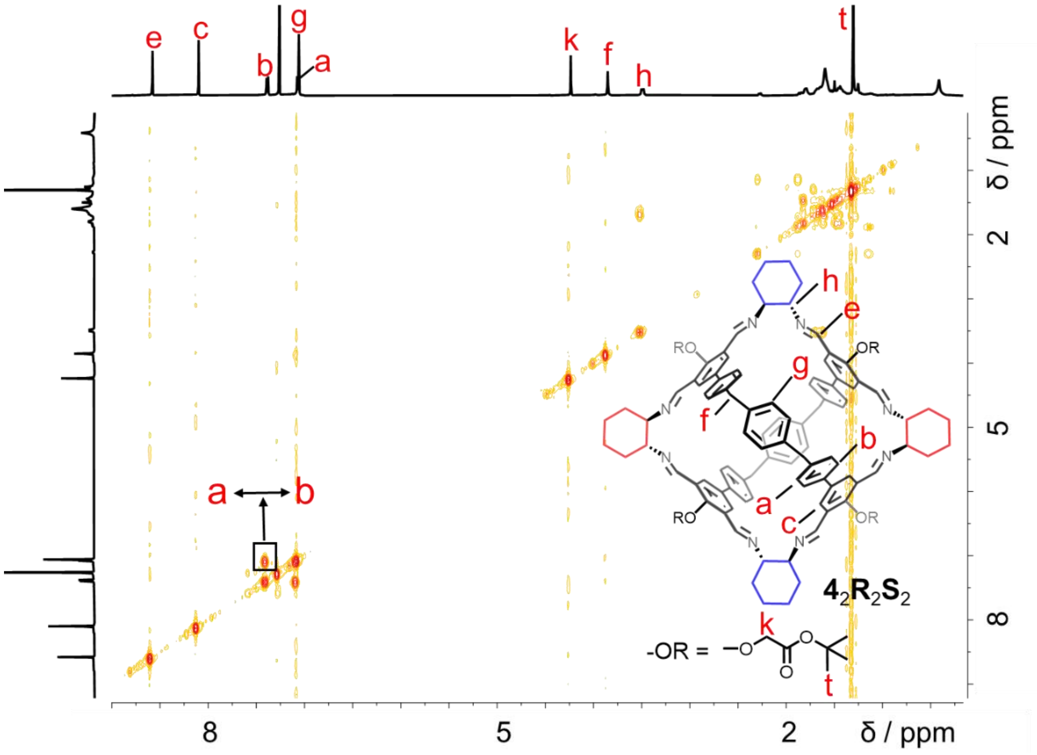

Supplementary Figure 66. NMR characterization. ^{1}H−^{1}H COSY spectrum (500 MHz, $CDCl_3$, 298 K) of **$4_2R_2S_2$**. Key correlation peaks are labeled in the spectrum.

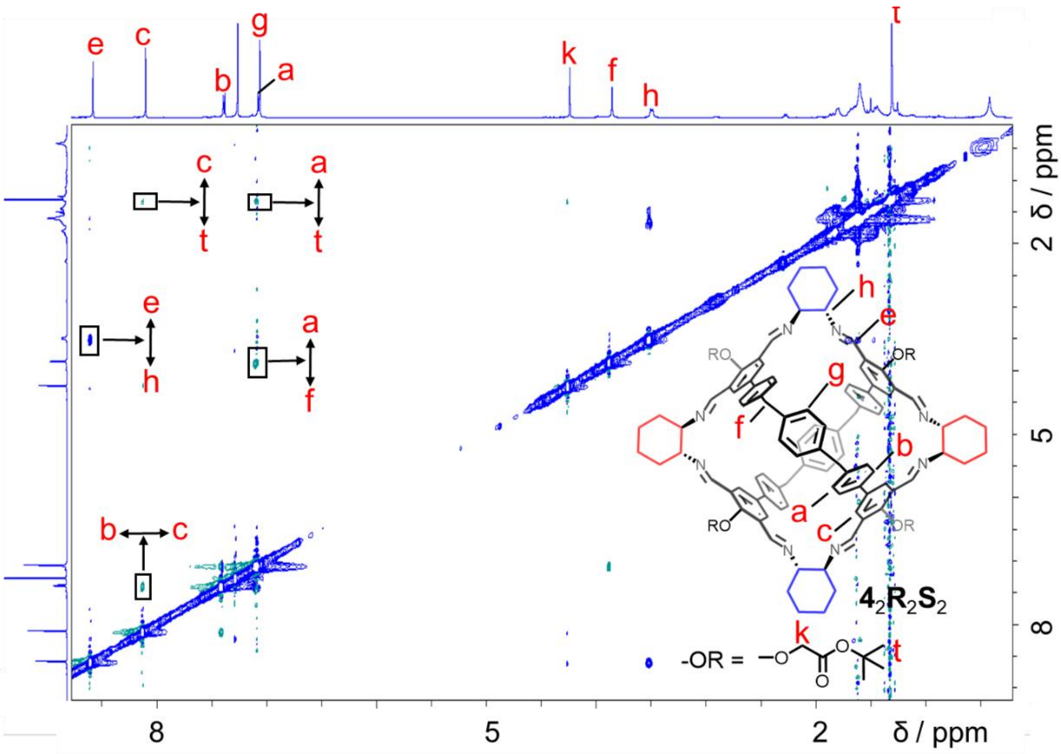

Supplementary Figure 67. NMR characterization. ^{1}H−^{1}H NOESY spectrum (500 MHz, $CDCl_3$, 298 K) of **$4_2R_2S_2$**. Key correlation peaks are labeled in the spectrum.

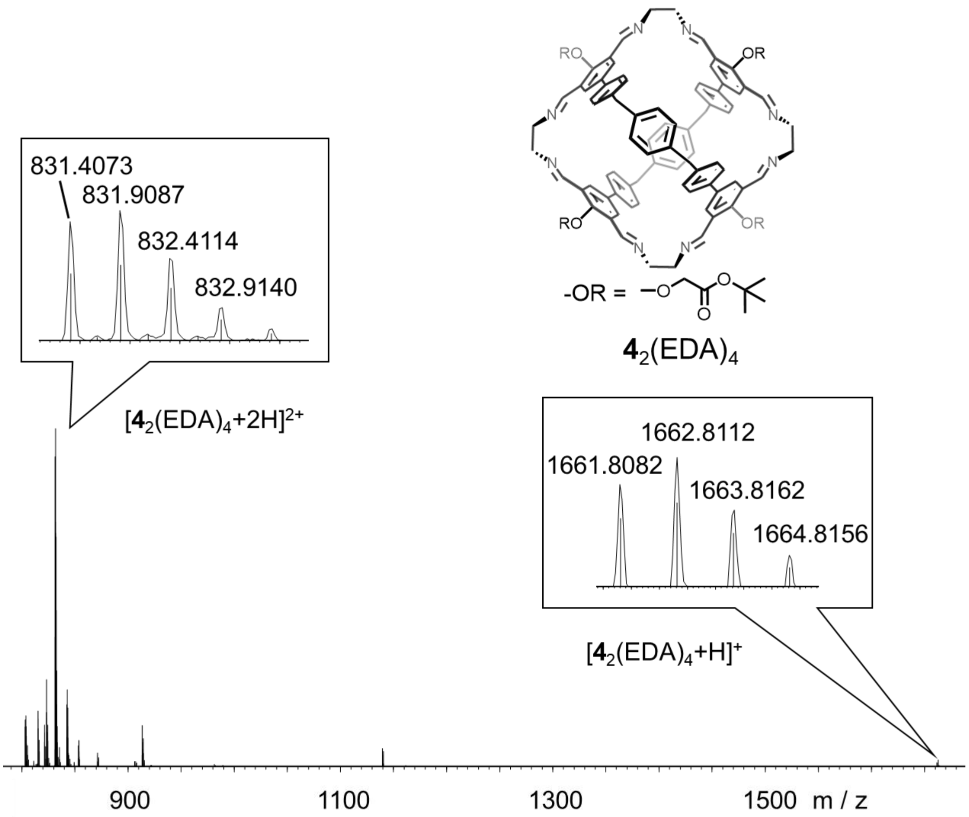

Supplementary Figure 68. High-resolution LCMS-IT-TOF of $\mathbf{4_2}$(EDA)$_4$. The signals labeled in the spectrum correspond to molecular cations that contain two and one positive charges, respectively. *m/z* [$\mathbf{4}_2$(EDA)$_4$+2H]$^{3+}$ calculated for $C_{104}H_{110}N_8O_{12}^{3+}$: 831.9133; found: 831.9140. [$\mathbf{4}_2$(EDA)$_4$+H]$^+$ calculated for $C_{104}H_{109}N_8O_{12}^{2+}$: 1662.8194; found: 1662.8112.

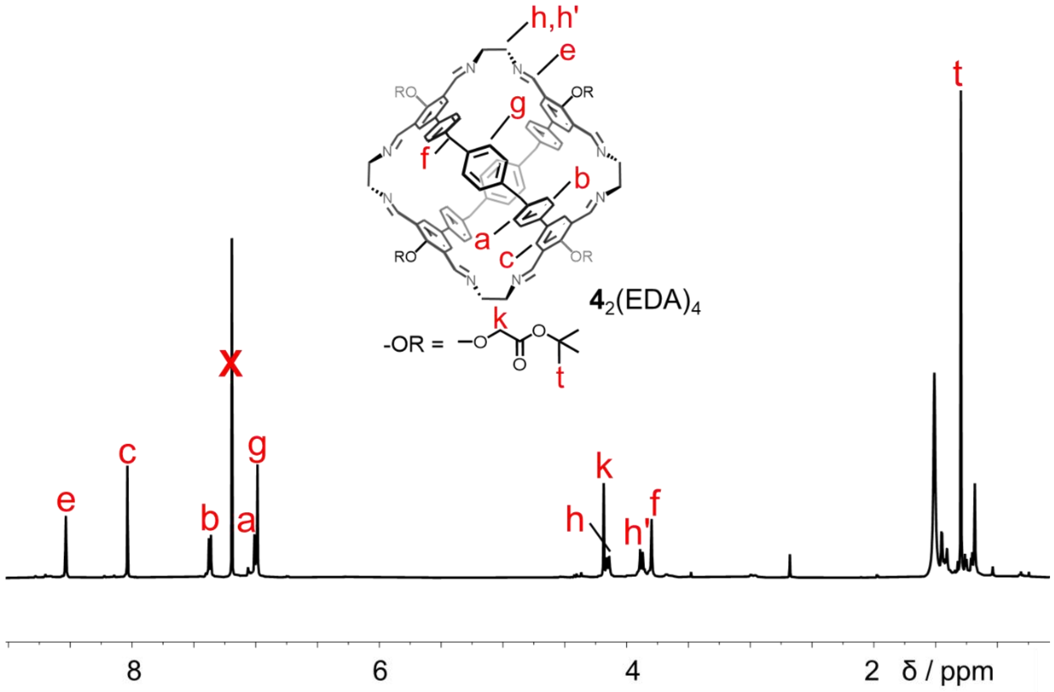

Supplementary Figure 69. NMR characterization. Partial ^{1}H NMR spectrum of $\mathbf{4}_2(EDA)_4$ (500 MHz, $CDCl_3$, 298 K).

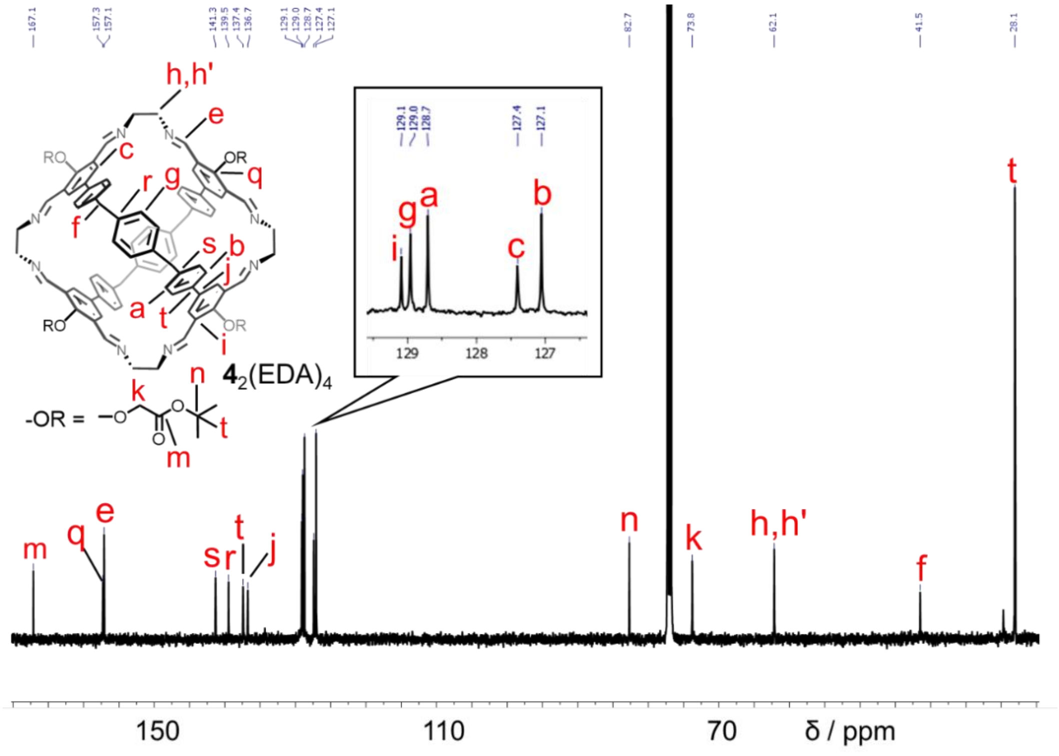

Supplementary Figure 70. NMR characterization. ^{13}C NMR spectrum (125 MHz, $CDCl_3$, 298 K) of $\mathbf{4}_2(EDA)_4$.

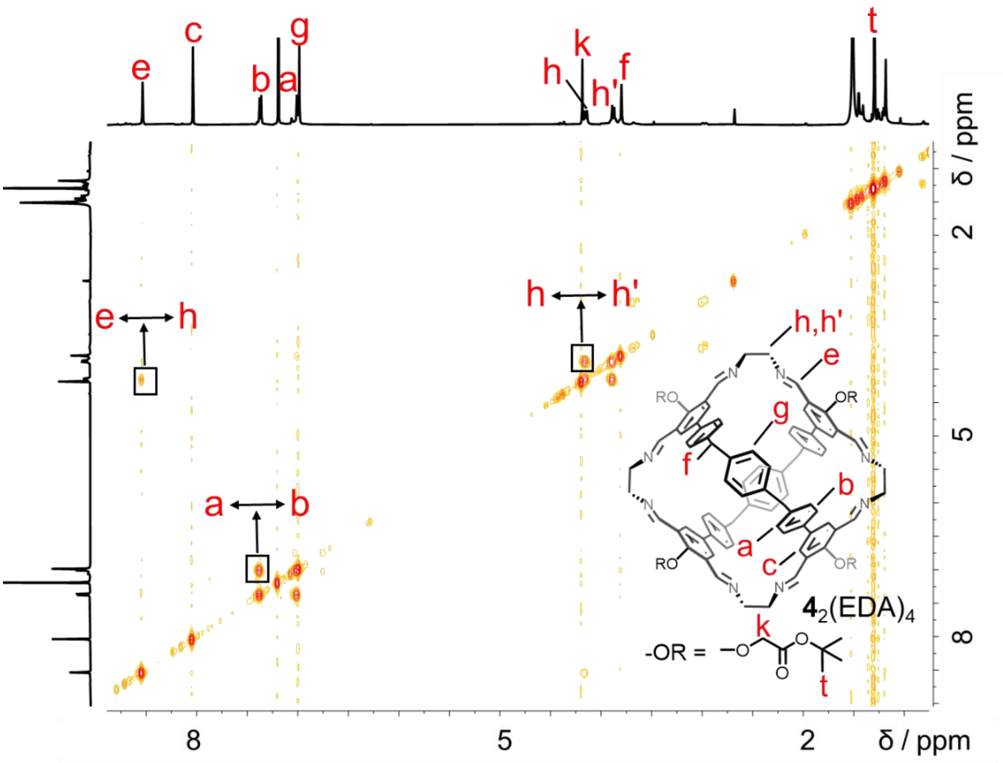

Supplementary Figure 71. NMR characterization. $^{1}H-^{1}H$ COSY spectrum (500 MHz, $CDCl_3$, 298 K) of $\mathbf{4}_2(EDA)_4$. Key correlation peaks are labeled in the spectrum.

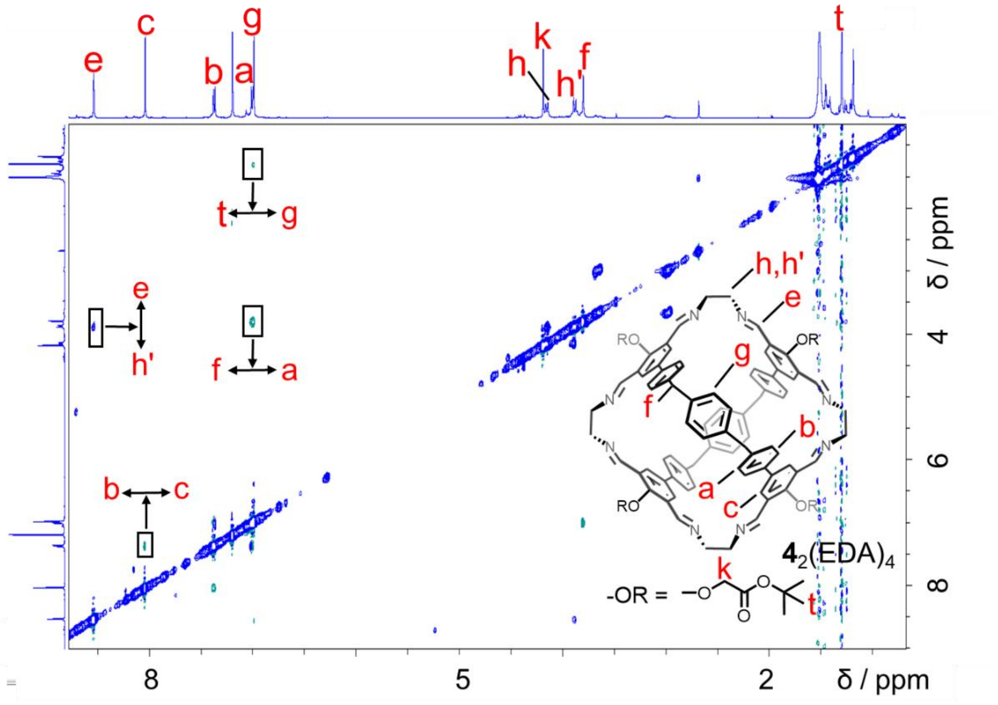

Supplementary Figure 72. NMR characterization. $^{1}H-^{1}H$ NOESY spectrum (500 MHz, $CDCl_3$, 298 K) of $\mathbf{4}_2(EDA)_4$. Key correlation peaks are labeled in the spectrum.

8. Transformation between cages

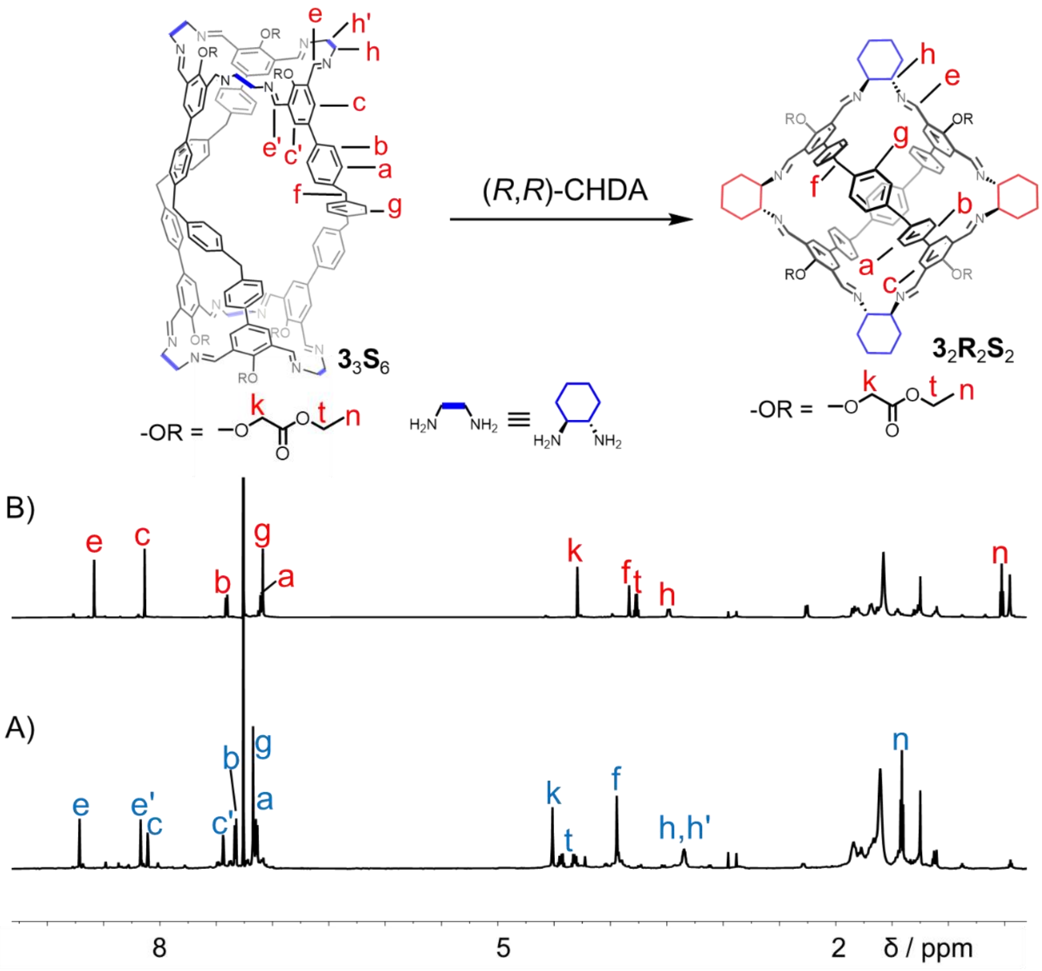

Supplementary Figure 73. The transformation from 3_3S_6 to $3_2R_2S_2$. The ^{1}H NMR spectra (600 MHz, $CDCl_3$, 298 K) of $\mathbf{3_3S_6}$ A) before and B) after adding three equivalents of (*R*,*R*)-CHDA.

We added three equivalents of (*R*,*R*)-CHDA into a $CDCl_3$ solution of $\mathbf{3_3S_6}$. After heating the mixture at 50 °C for 12 h, the resonances corresponding to $\mathbf{3_3S_6}$ almost completely disappeared, accompanied with the appearance of a new set of resonances corresponding to $\mathbf{3_2R_2S_2}$ (Supplementary Figure 73B). This observation indicates that $\mathbf{3_2R_2S_2}$ is more thermodynamically stable than $\mathbf{3_3S_6}$.

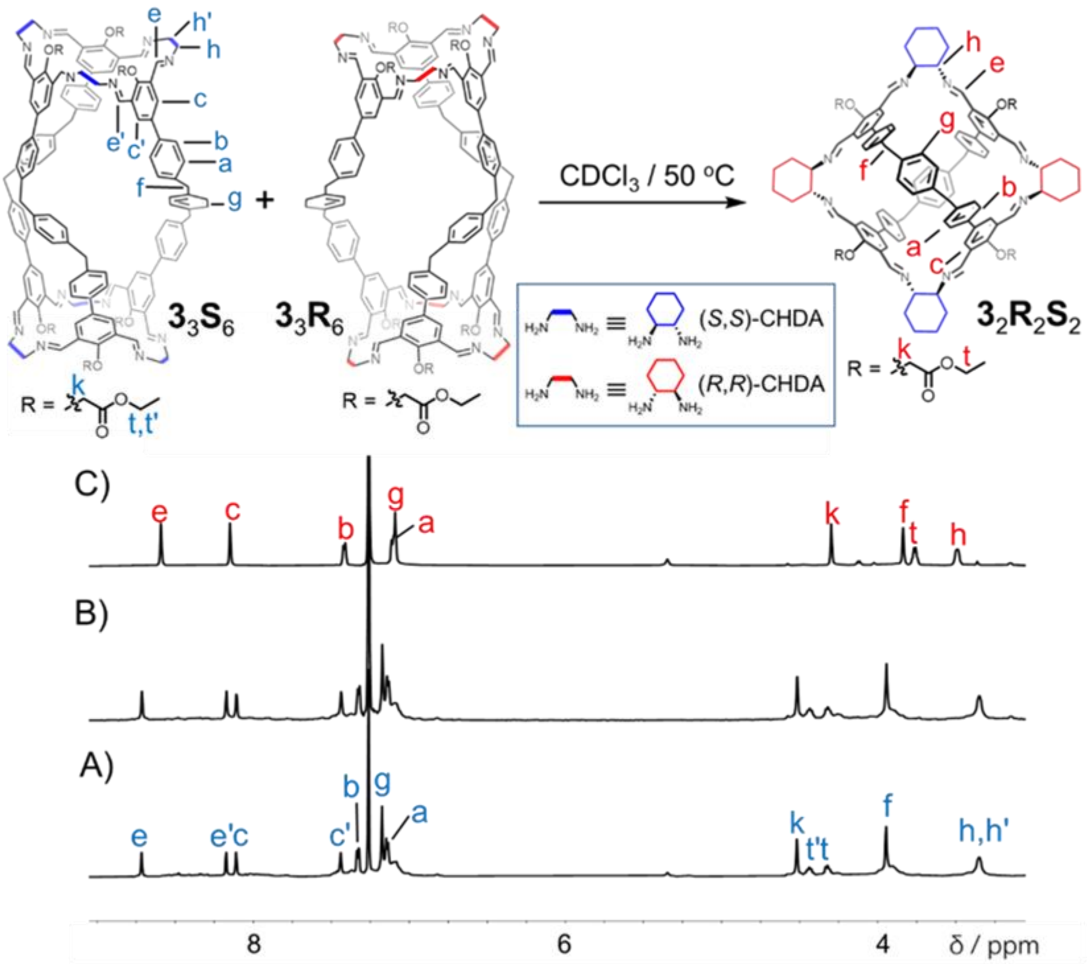

Supplementary Figure 74. The transformation from the mixture of 3_3S_6 and 3_3R_6 to $3_2R_2S_2$. Partial ^{1}H NMR spectra (400 MHz, $CDCl_3$, 298 K) of A) $\mathbf{3_3S_6}$, B) $\mathbf{3_3R_6}$ and C) the 1:1 reaction mixture of $\mathbf{3_3S_6}$ and $\mathbf{3_3R_6}$.

When two pre-synethesized [3+6] cage enantiomers, namely $\mathbf{3_3R_6}$ and $\mathbf{3_3S_6}$, were mixed, they would be completely transformed into a more stable achiral cage, namely $\mathbf{3_2R_2S_2}$. This experiment result was strongly confirmed by the corresponding ^{1}H NMR spectrum (Supplementary Figure 74). This result indicates that the achiral cage $\mathbf{3_2R_2S_2}$ is more favored in terms of entropy compared with the chiral cage $\mathbf{3_3R_6}$ or $\mathbf{3_3S_6}$, because the latter is composed of fewer building blocks compared to the former.

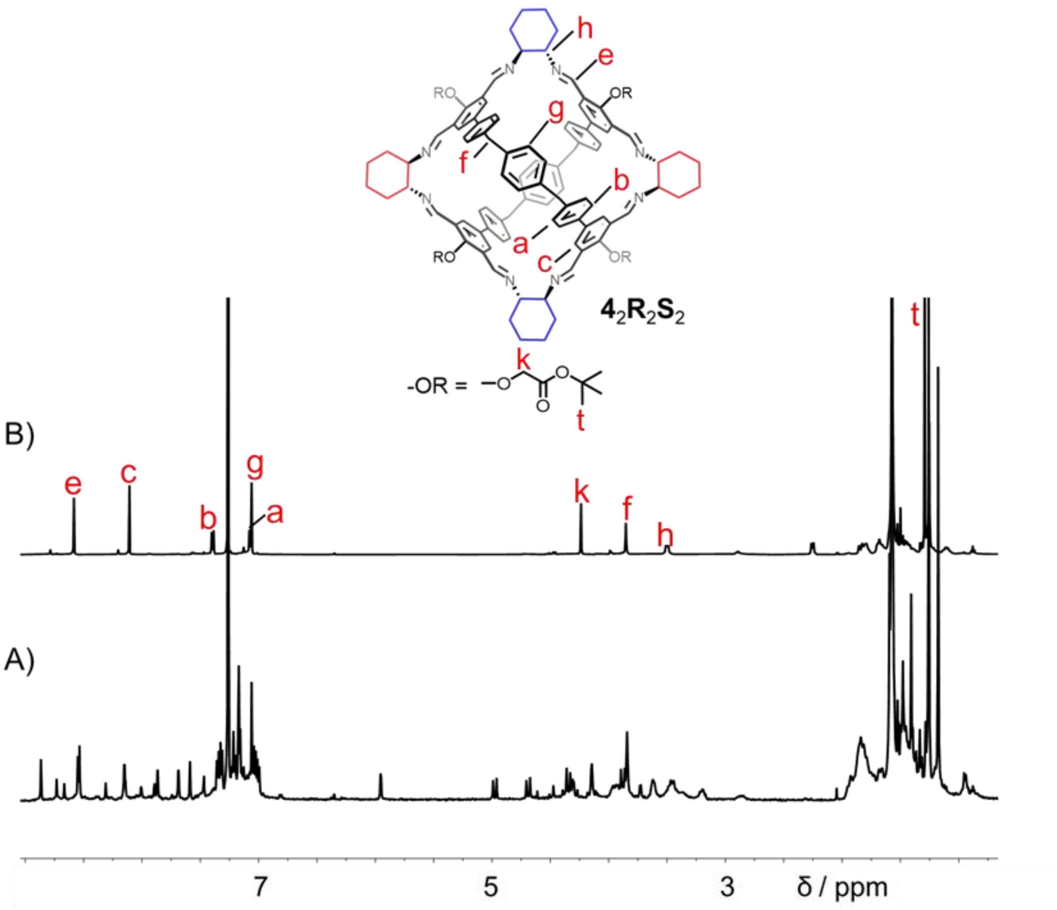

Supplementary Figure 75. The transformation from the reaction mixture to $4_2R_2S_2$. The ^1H NMR spectra (500 MHz, $CDCl_3$, 298 K) of the reaction mixture by condensing two equivalents of **2** and four equivalents of (*S,S*)-CHDA, A) before and B) after adding two equivalents of (*R,R*)-CHDA.

9. The self-assembly yields of cages

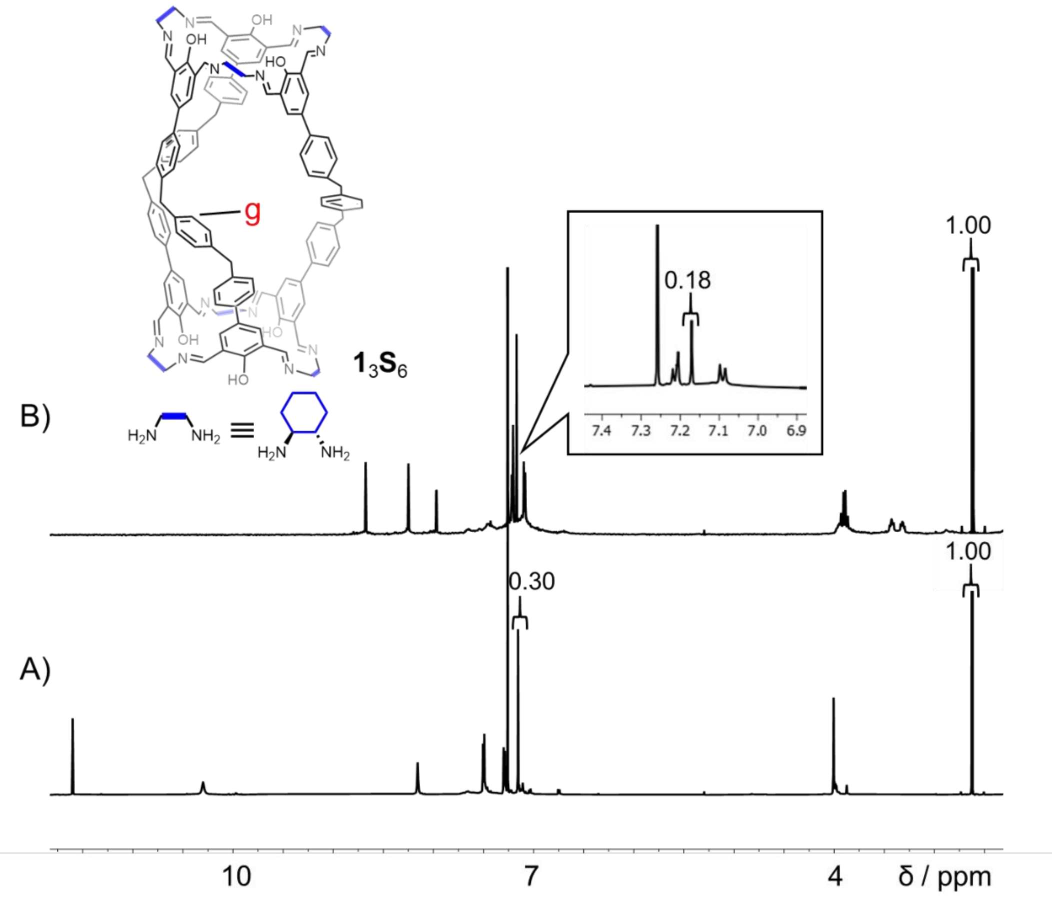

Supplementary Figure 76. The NMR yield of $\mathbf{1_3S_6}$. ^{1}H NMR spectra (600 MHz, $CDCl_3$, 298 K) of A) **1** and B) $\mathbf{1_3S_6}$.

The yields of the corresponding cages were determined by using DMSO with a fixed concentration as the internal standard in the corresponding ^{1}H NMR samples of both the precursors and the corresponding self-assembly mixture. The integration of the resonance of the internal standard namely DMSO was normalized to 1, allowing the concentration ratios of the self-assembled products relative to their corresponding aldehyde precursors to be calculated. For example, in the ^{1}H NMR spectra (Supplementary Figure 76) of **1** and $\mathbf{1_3S_6}$, whose spectra were recorded before and after adding (*S*,*S*)-CHDA, the resonances of the hydrogens in *g* position were integrated as 0.30 and 0.18, respectively, relative to the standard; the yield of $\mathbf{1_3S_6}$ was thus calculated to be 60% (0.18/0.30). The yields mentioned below were all calculated via this method.

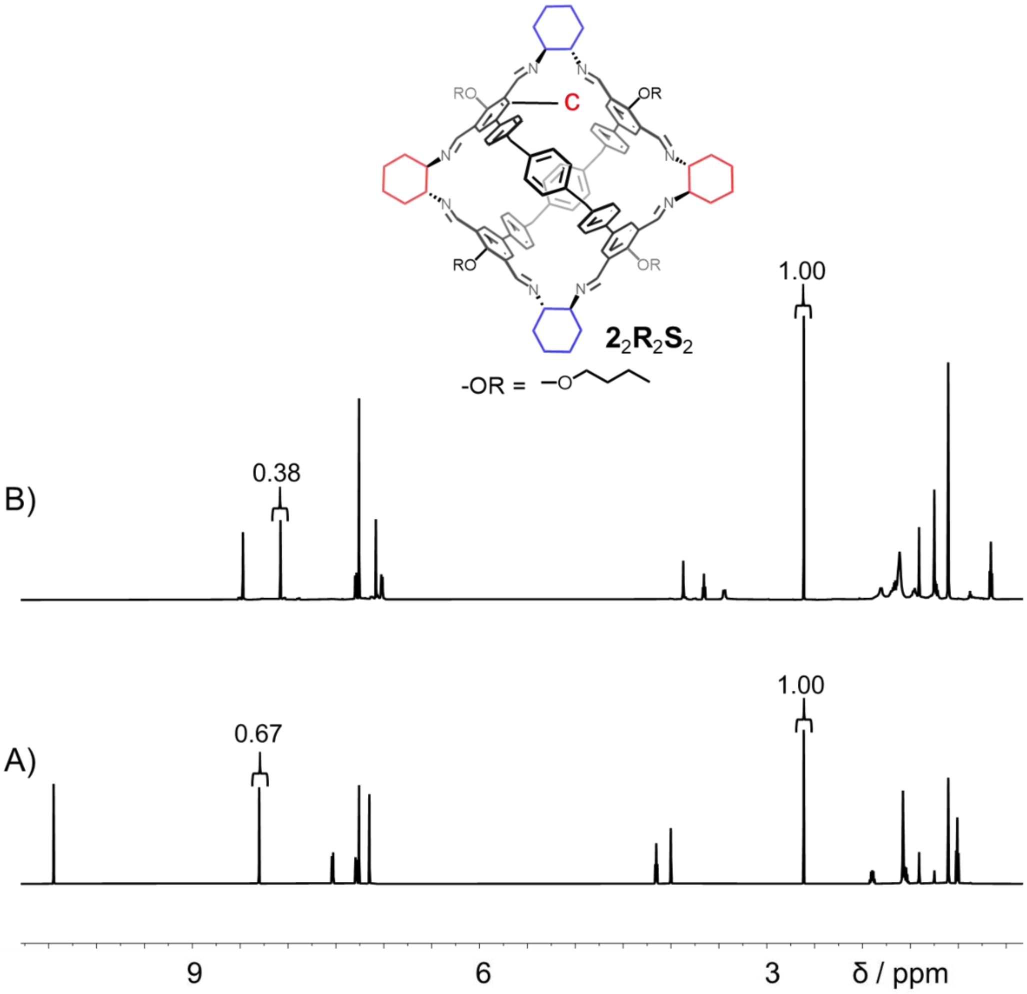

Supplementary Figure 77. The NMR yield of $\mathbf{2_2R_2S_2}$. ^{1}H NMR spectra (600 MHz, $CDCl_3$, 298 K) of A) **2** and B) $\mathbf{2_2R_2S_2}$. The NMR yield of $\mathbf{2_2R_2S_2}$ is 57%.

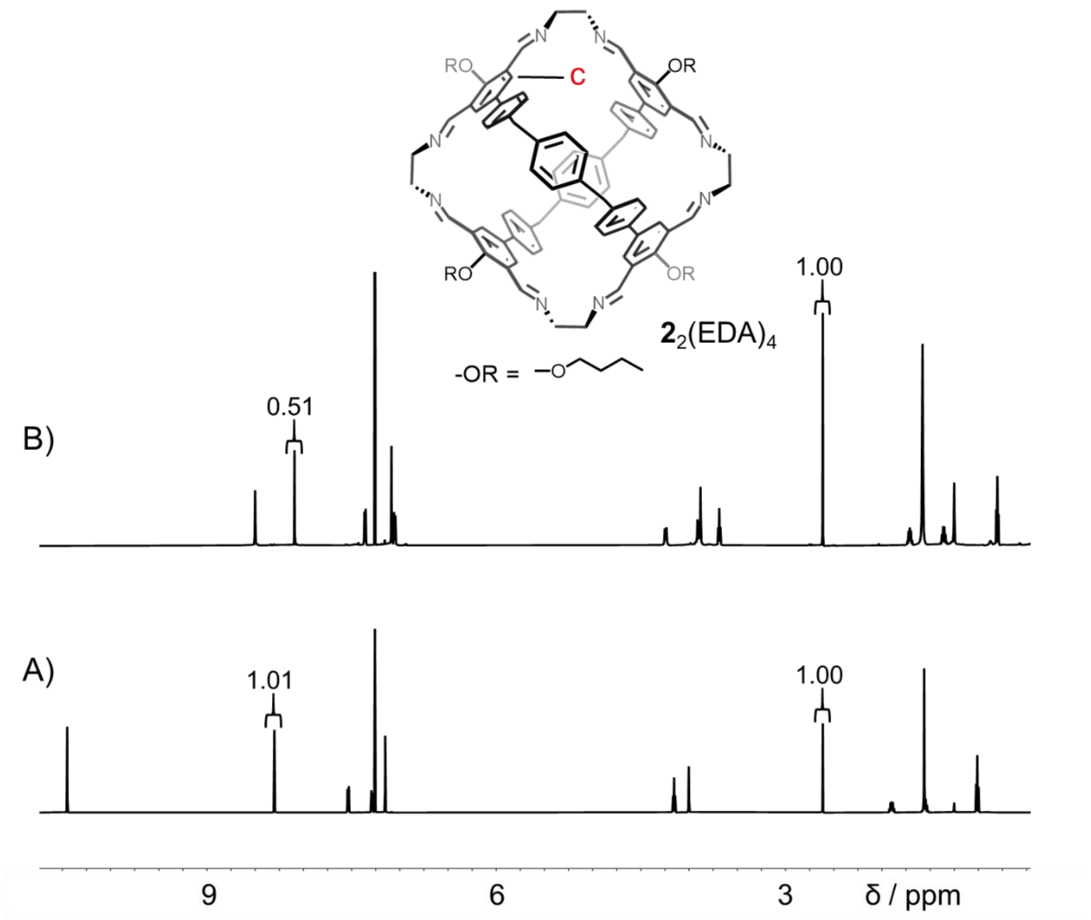

Supplementary Figure 78. The NMR yield of 2_2(EDA)$_4$. ^{1}H NMR spectra (600 MHz, $CDCl_3$, 298 K) of A) **2** and B) **2**$_2$(EDA)$_4$. The NMR yield of **2**$_2$(EDA)$_4$ is 50%.

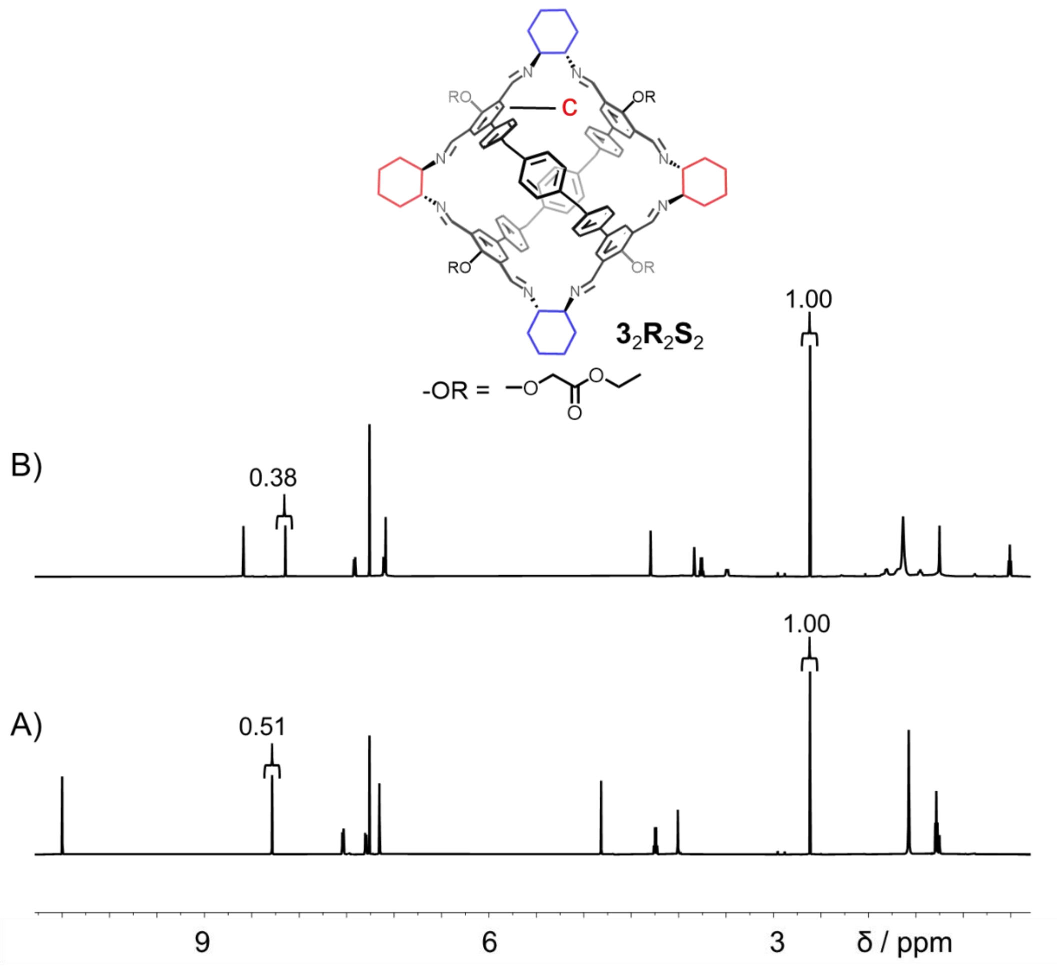

Supplementary Figure 79. The NMR yield of $\mathbf{3_2R_2S_2}$. ^{1}H NMR spectra (600 MHz, $CDCl_3$, 298 K) of A) **3** and B) $\mathbf{3_2R_2S_2}$. The NMR yield of $\mathbf{3_2R_2S_2}$ is 75%.

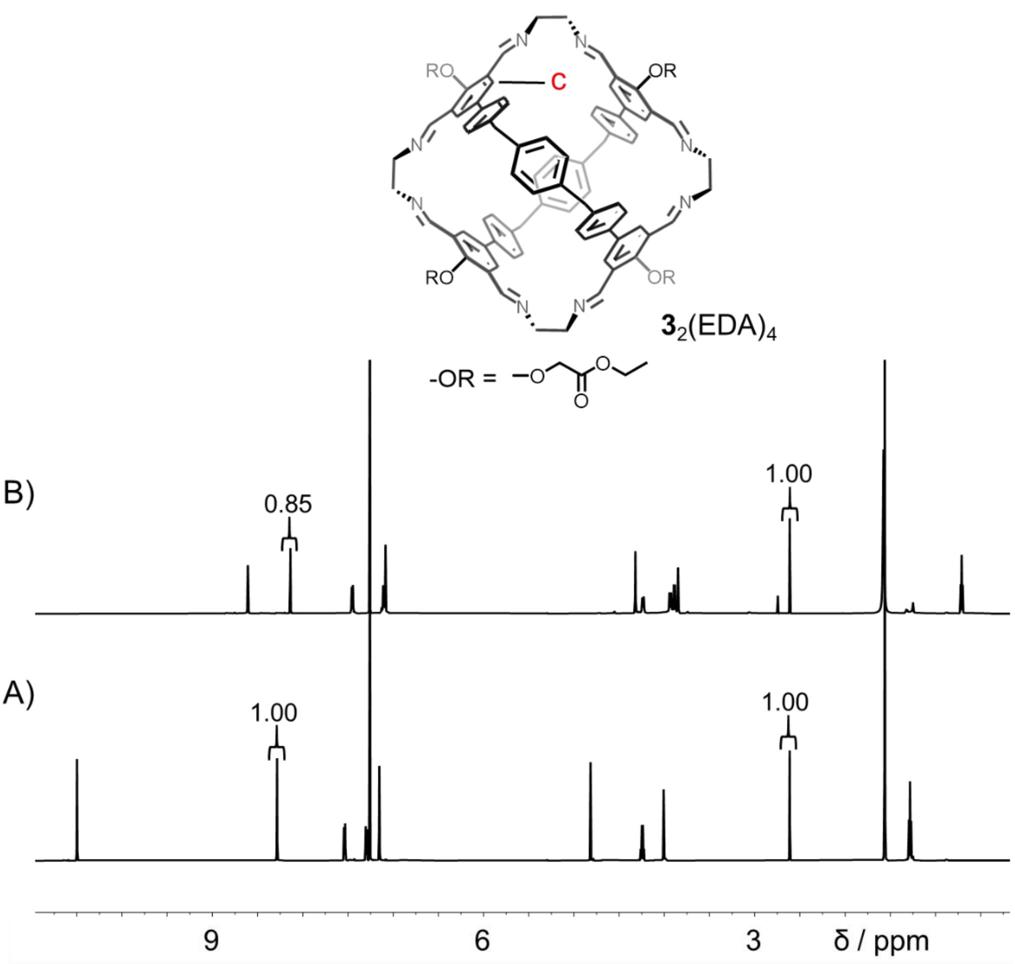

Supplementary Figure 80. The NMR yield of 3_2(EDA)$_4$. ^{1}H NMR spectra (600 MHz, $CDCl_3$, 298 K) of A) **3** and B) **3**$_2$(EDA)$_4$. The NMR yield of **3**$_2$(EDA)$_4$ is 85%.

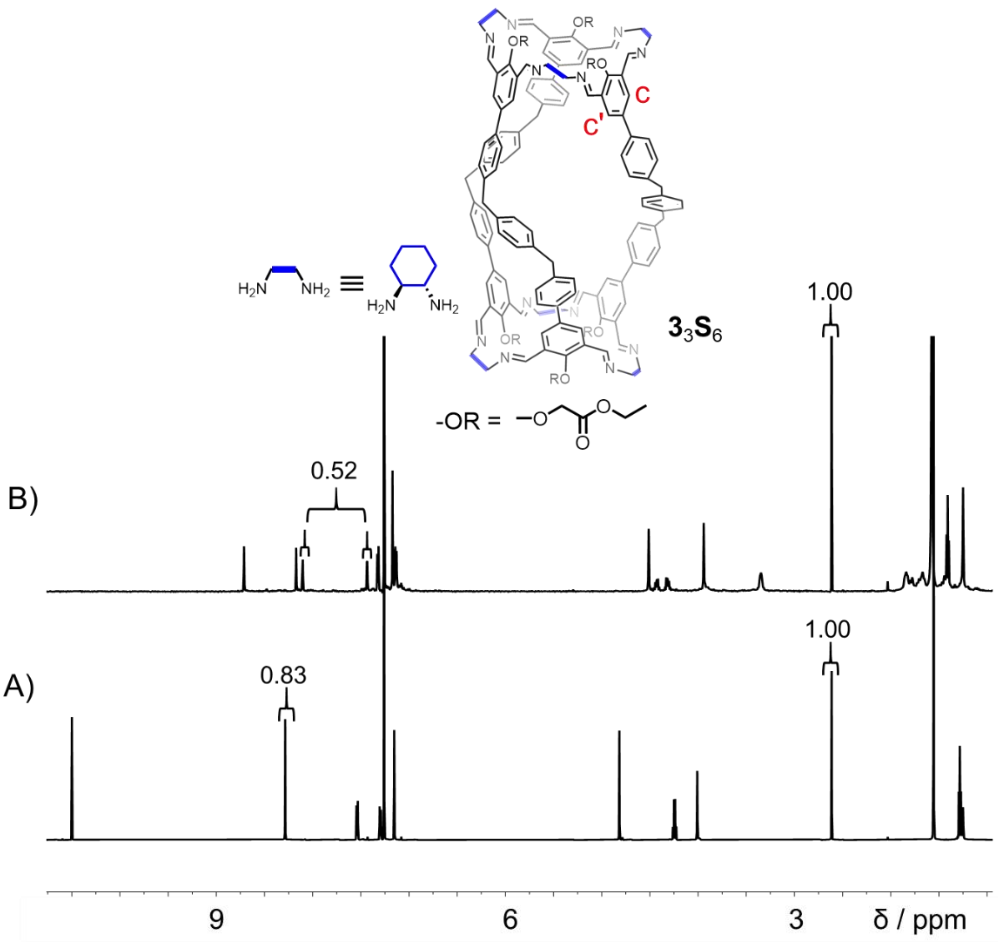

Supplementary Figure 81. The NMR yield of 3_3S_6. ^{1}H NMR spectra (600 MHz, $CDCl_3$, 298 K) of A) **3** and B) $\mathbf{3_3S_6}$. The NMR yield of $\mathbf{3_3S_6}$ is 63%.

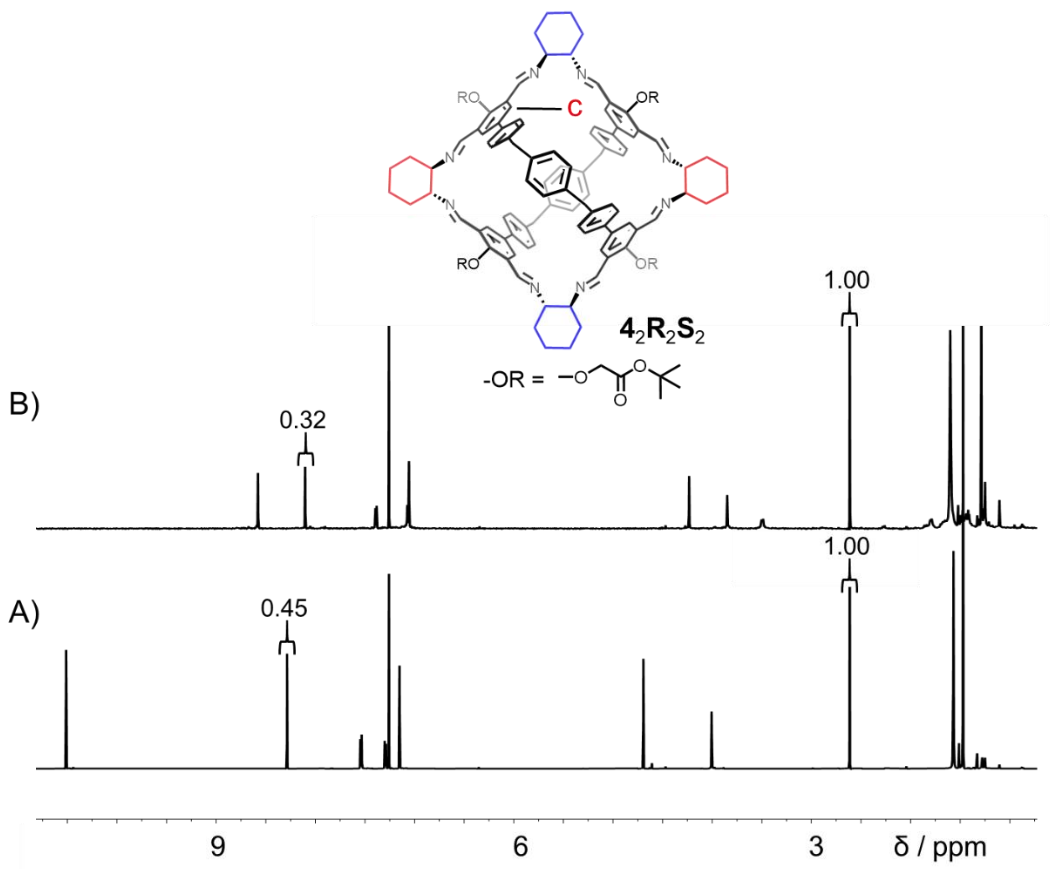

Supplementary Figure 82. The NMR yield of $\mathbf{4_2R_2S_2}$. ^{1}H NMR spectra (600 MHz, $CDCl_3$, 298 K) of A) **4** and B) $\mathbf{4_2R_2S_2}$. The NMR yield of $\mathbf{4_2R_2S_2}$ is 71%.

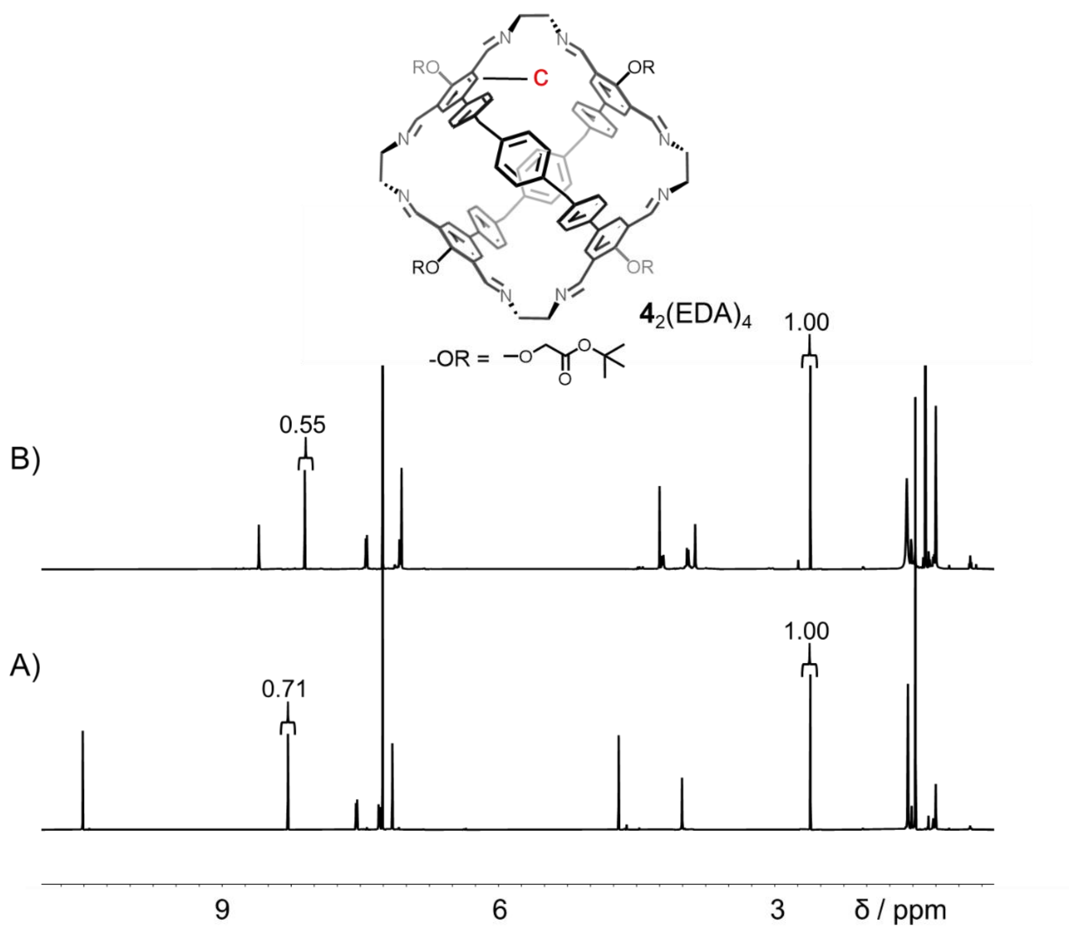

Supplementary Figure 83. The NMR yield of $\mathbf{4_2(EDA)_4}$. ^1H NMR spectra (600 MHz, $CDCl_3$, 298 K) of A) **4** and B) $\mathbf{4}_2(EDA)_4$. The NMR yield of $\mathbf{4}_2(EDA)_4$ is 77%.

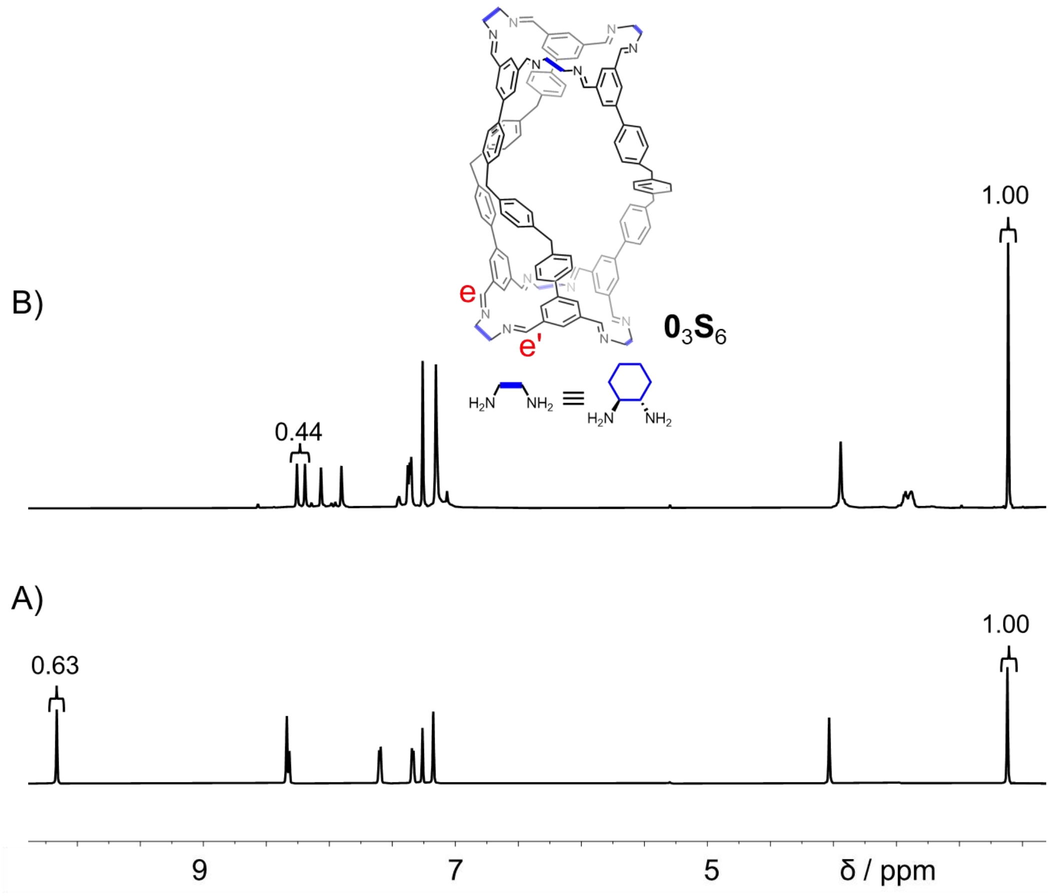

Supplementary Figure 84. The NMR yield of $\mathbf{0_3S_6}$. ^{1}H NMR spectra (600 MHz, $CDCl_3$, 298 K) of A) **0** and B) $\mathbf{0_3S_6}$. The NMR yield of $\mathbf{0_3S_6}$ is 70%.

10. X-ray Crystallography

1) $\mathbf{2_2R_2S_2}$

1.1) Methods

Single crystal of the cage $\mathbf{2_2R_2S_2}$ was obtained by slow vapor diffusion of methanol into the $CHCl_3$ solution under room temperature. A suitable crystal was selected on a Bruker D8 Venture diffractometer. The crystal was kept at 213.0 K during data collection. Using Olex2 [1], the structure was solved with the ShelXT [2] structure solution program using Intrinsic Phasing and refined with the ShelXL [3] refinement package using Least Squares minimisation.

1.2) Crystal data

[$C_{112}H_{124}N_8O_4$] (M =1646.18 g/mol): monoclinic, space group P 1 21/c 1, a = 15.5144(3) Å, b = 27.2953(6) Å, c = 27.2049(6) Å, α = 90°, β = 105.1580(10)°, γ = 90°, V = 11119.6(4) Å^{3,} Z = 4, T = 213.0 K, μ(GaKα) = 0.295 mm^{-1}, $Dcalc$ = 0.983 g/cm^3, 101559 reflections measured (2.817° ≤ 2Θ ≤ 55.007°), 21133 unique (R_{int} = 0.0822) which were used in all calculations. The final R_1 was 0.1029 (I > 2σ(I)) and wR_2 was 0.2952 (all data). CCDC number: 2201823.

1.3) Solid-state structure

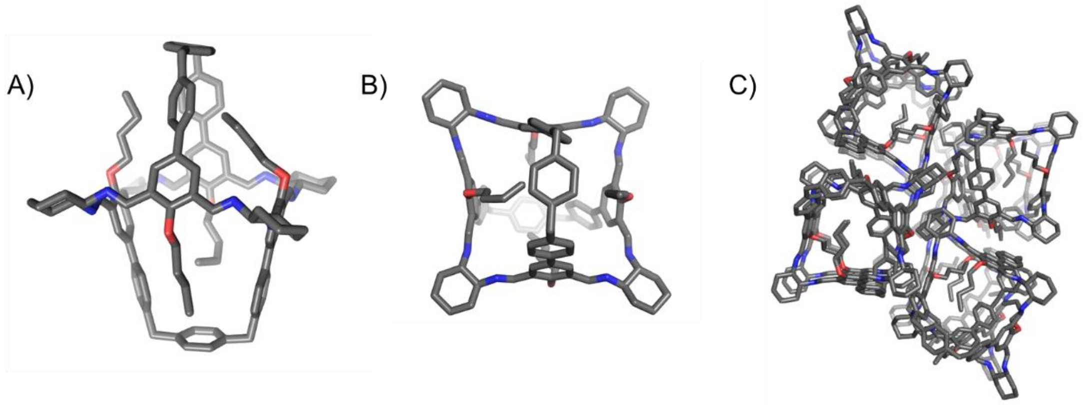

Supplementary Figure 85. Single-crystal structure and packing mode of $\mathbf{2_2R_2S_2}$. A) Side view and B) top view of the single-crystal structure of $\mathbf{2_2R_2S_2}$; C) packing in the solid state, view from a-axis. Solvent molecules are omitted for the sake of clarity.

The molecules, namely $\mathbf{2_2R_2S_2}$, pack through π–π interactions, CH-π interactions and

hydrogen bonds. The cavities of molecular cages are occupied by alkyloxy substituents, resulting in narrow channels in the packing mode of the single-crystal structure.

2) $\mathbf{3_2R_2S_2}$

2.1) Methods

Single crystal of the cage $\mathbf{3_2R_2S_2}$ was obtained by slow evaporation of diethyl ether into the $CHCl_3$ solution under room temperature. A suitable crystal was selected on a Bruker D8 Venture diffractometer. The crystal was kept at 213.0 K during data collection. Using Olex2 [1], the structure was solved with the ShelXT [2] structure solution program using Intrinsic Phasing and refined with the ShelXL [3] refinement package using Least Squares minimisation.

2.2) Crystal data

[$C_{112}H_{116}N_8O_{12}$] (M =1766.12 g/mol): Monoclinic, space group C 1 2/c 1, a = 42.8966(11) Å, b = 18.1515(4) Å, c = 32.1229(7) Å, α = 90°, β = 106.437(2)°, γ = 90°, V = 23989.9(10) Å^3, Z = 8, T = 213.0 K, μ(GaKα) = 0.325 mm^{-1}, $Dcalc$ =0.978 Mg/m^3, 133902 reflections measured (3.205° ≤ 2Θ ≤ 54.887°), 22712 unique (R_{int} = 0.0651) which were used in all calculations. The final R_1 was 0.1167 (I > 2σ(I)) and wR_2 was 0.3279 (all data). CCDC number: 2201825.

2.3) Solid-state structure

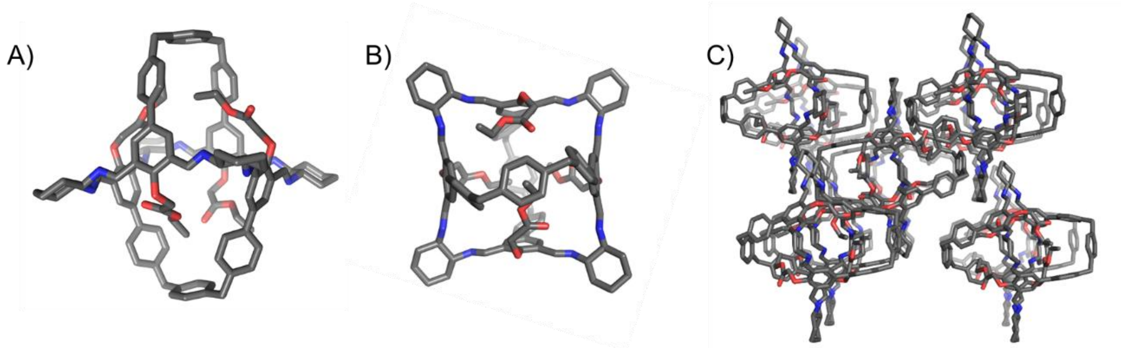

Supplementary Figure 86. Single-crystal structure and packing mode of $\mathbf{3_2R_2S_2}$. A) Side view and B) top view of the single-crystal structure of $\mathbf{3_2R_2S_2}$; C) packing in the solid state, view from b-axis. Solvent molecules are omitted for the sake of clarity.

The molecules, namely $\mathbf{3_2R_2S_2}$, pack through π–π interactions, CH-π interactions and hydrogen bonds. The cavities of molecular cages are occupied by ester substituents,

resulting in narrow channels in the stacking mode of the single-crystal structure.

3) $\mathbf{1_3S_6}$

3.1) Methods

Single crystal of the cage $\mathbf{1_3S_6}$ was obtained by slow evaporation of CH_3CN into the DMF solution under room temperature. A suitable crystal was selected on a Bruker D8 Venture diffractometer. The crystal was kept at 213.0 K during data collection. Using Olex2 [1], the structure was solved with the ShelXT [2] structure solution program using Intrinsic Phasing and refined with the ShelXL [3] refinement package using Least Squares minimisation.

3.2) Crystal data

[$C_{114}H_{138}N_{12}O_6$] (M =2132.66 g/mol): Tetragonal, space group $P4_12_12$, a = 22.8393(16) Å, b = 22.8393(16) Å, c = 24.658(3) Å, α = 90°, β = 90°, γ = 90°, V =12863(2) Å^3, Z = 4, T = 213.0 K, μ(GaKα) = 0.335 mm^{-1}, $Dcalc$ =1.101 Mg/m^3, 98171 reflections measured (2.380° ≤ 2Θ ≤ 55.046°), 12260 unique (R_{int} = 0.1215) which were used in all calculations. The final R_1 was 0.0807 (I > 2σ(I)) and wR_2 was 0.3029 (all data). CCDC number: 2203208.

3.3) Solid-state structure

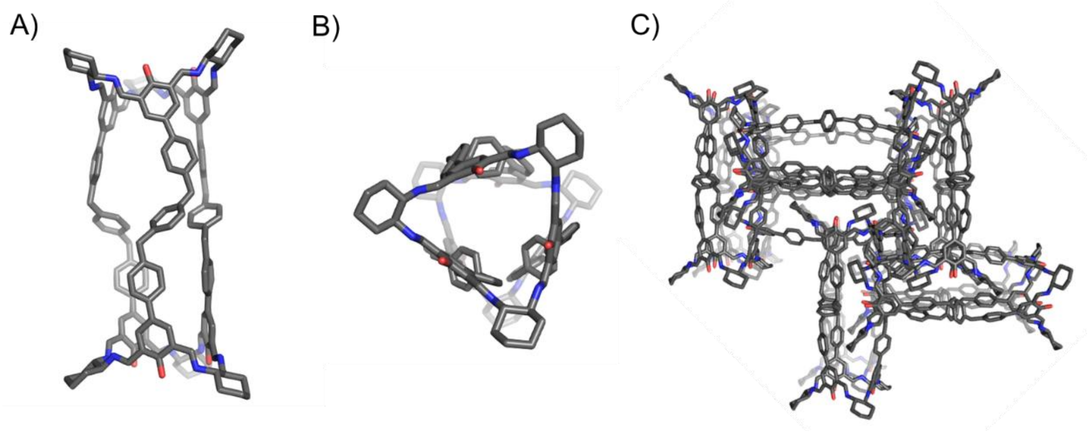

Supplementary Figure 87. Single-crystal structure and packing mode of $\mathbf{1_3S_6}$. A) Side view and B) top view of the single-crystal structure of $\mathbf{1_3S_6}$; C) packing in the solid state, view from c-axis. Solvent molecules are omitted for the sake of clarity.

The molecules, namely $\mathbf{1_3S_6}$, pack through π–π interactions, CH-π interactions and

hydrogen bonds. The molecular cages are divided into two arrangement forms in a mutually perpendicular manner. Cyclohexane diamine residues at ports of horizontally arranged cages inserted into the cavities of vertically arranged cages, resulting in no obvious channels in the stacking mode of the single-crystal structure.

4) Mixture of $\mathbf{1}_3\mathbf{S}_6$ and $\mathbf{1}_3\mathbf{R}_6$

4.1) Methods

Single crystals of the mixture of $\mathbf{1}_3\mathbf{S}_6$ and $\mathbf{1}_3\mathbf{R}_6$ was obtained by slow evaporation of methanol into the $CHCl_3$ solution under room temperature. A suitable crystal was selected on a Bruker D8 Venture diffractometer. The crystal was kept at 213.0 K during data collection. Using Olex2 [1], the structure was solved with the ShelXT [2] structure solution program using Intrinsic Phasing and refined with the ShelXL [3] refinement package using Least Squares minimisation.

4.2) Crystal data

[$C_{114}H_{138}N_{12}O_6$] (*M* =2132.66 g/mol): Tetragonal, Triclinic, P-1, a = 17.0137(11) Å, b = 18.2192(12) Å, c = 25.675(2) Å, α = 69.245(4)°, β = 85.794(6)°, γ = 83.136(4)°, V =7384.6(9) Å^3, Z = 2, T = 213.0 K, μ(GaKα) = 0.292 mm^{-1}, *Dcalc* = 0.959 Mg/m^3, 67246 reflections measured (3.013° ≤ 2Θ ≤ 49.654°), 22597 unique (R_{int} = 0.0961) which were used in all calculations. The final R_1 was 0.1471 ($I > 2\sigma(I)$) and wR_2 was 0.3597 (all data). CCDC number: 2203209.

4.3) Solid-state structure

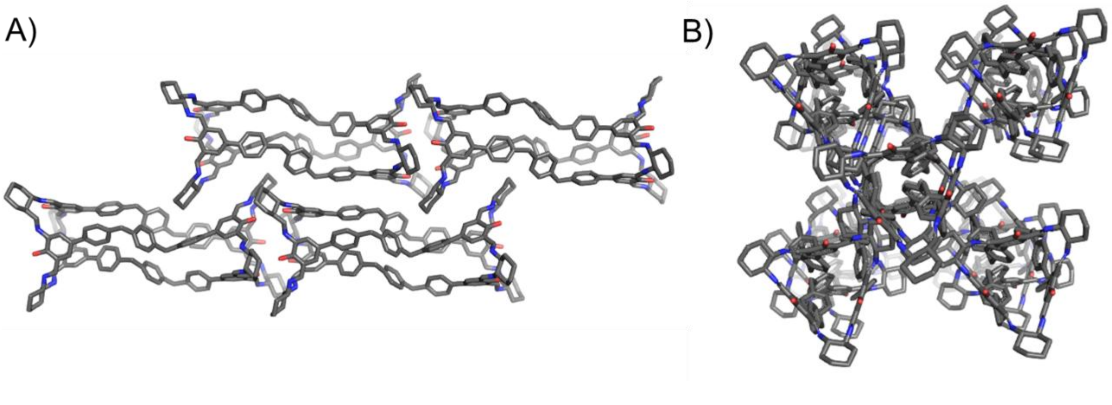

Supplementary Figure 88. Single-crystal structure and packing mode of the mixture of $\mathbf{1}_3\mathbf{S}_6$ and $\mathbf{1}_3\mathbf{R}_6$. Different views of the packing mode of solid-state structure of

the mixture of **1**$_3$**S**$_6$ and **1**$_3$**R**$_6$, from A) a-axis, B) c-axis. Solvent molecules are omitted for the sake of clarity.

Two kinds of enantiomers, namely **1**$_3$**S**$_6$ and **1**$_3$**R**$_6$, pack through π–π interactions, CH-π interactions and hydrogen bonds. Molecular cages with the same configuration arrange in a head to tail manner along the crystallographic c-axis. Due to the collapse of the cage cavities, there are just narrow channels in the stacking mode of the single-crystal structure.

11. Supplementary theoretical calculations

All investigated cage structures were optimized by using the density functional theory (DFT) at the BP86-D3/6-311G(d) level with the Gaussian 16 package[1]. The solvent effect of chloroform was included with the polarizable continuum model (PCM)[2]. All the optimized structures were verified by the phonon frequencies calculated at the same level (namely no imaginary frequency should exist).

Based on the structure of $\mathbf{2}_2\mathbf{R}_2\mathbf{S}_2$, we replaced two (*R,R*)-CHDA residues with two (*S,S*)-CHDA residues and got a putative cage $\mathbf{2}_2\mathbf{S}_4$, in which four of the eight imine protons adopt the *anti* conformation with respect to the adjacent methine protons. In a similar way, we replaced all CHDA residues of $\mathbf{2}_2\mathbf{R}_2\mathbf{S}_2$ with different configurational CHDA residues and got a putative cage $\mathbf{2}_2\mathbf{S}_2\mathbf{R}_2$, in which all of the eight imine protons adopt the *anti* conformation (Figure 6). The DFT calculations revealed that the relative free energies of $\mathbf{2}_2\mathbf{R}_2\mathbf{S}_2$, $\mathbf{2}_2\mathbf{S}_4$ and $\mathbf{2}_2\mathbf{S}_2\mathbf{R}_2$ are 0 kcal/mol, 5.4 kcal/mol and 9.1 kcal/mol, respectively. These results confirm that the *syn* conformer is thermodynamically more favored than the *anti* counterpart. As a consequent, $\mathbf{2}_2\mathbf{R}_2\mathbf{S}_2$, with all the imine protons in the *syn* conformation, is the most stable and favored product, which is fully consistent with the experimental results.

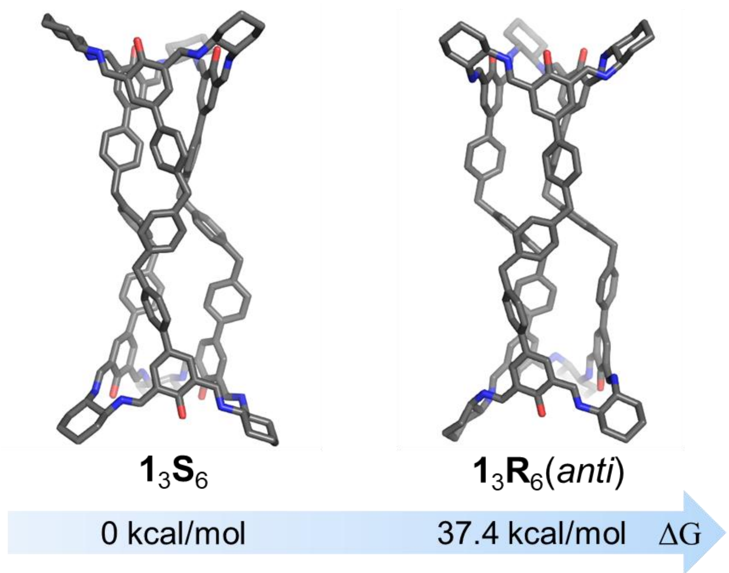

Supplementary Figure 89. Theoretical calculation. Optimized structures of different [3+6] cages, namely $\mathbf{1}_3\mathbf{S}_6$ and $\mathbf{1}_3\mathbf{R}_6$(*anti*), with the relative free energies.

Based on the structure of $\mathbf{1}_3\mathbf{S}_6$, we replaced all (*S,S*)-CHDA residues with (*R,R*)-CHDA residues and got a putative cage $\mathbf{1}_3\mathbf{R}_6$(*anti*), in which all imine protons adopt the *anti* conformation with respect to the adjacent methine protons (Supplementary Figure 89). The DFT calculations revealed that the relative free energies of $\mathbf{1}_3\mathbf{S}_6$ and $\mathbf{1}_3\mathbf{R}_6$(*anti*) are 0 kcal/mol and 37.4 kcal/mol, respectively. This results also confirm that the *syn* conformer is thermodynamically more favored than the *anti* counterpart, which is fully consistent with our experimental results.

12. Supplementary References

1. Frisch, M. J. et al. Gaussian 16 Revision B. 01. 2016. (Gaussian Inc., Wallingford CT).
2. Scalmani, G. & Frisch, M. J. Continuous surface charge polarizable continuum models of solvation. I. General formalism. *J. Chem. Phys.* **132**, 114110 (2010).
