# Supplementary material for: The sharp structural switch of covalent cages mediated by subtle variation of directing groups
Source: Nat Commun. 2023 Aug 2;14:4627. doi: 10.1038/s41467-023-40255-4 (PMC10397198; doi:10.1038/s41467-023-40255-4)
Supplement: Supplementary file 3 — Description of Additional Supplementary Files [file 41467_2023_40255_MOESM3_ESM.pdf]

Description of Additional Supplementary Files

File Name: Supplementary Data 1

Description: Cartesian Coordinates and Energies of All Investigated Cage Structures
