# Supplementary material for: The sharp structural switch of covalent cages mediated by subtle variation of directing groups
Source: Nat Commun. 2023 Aug 2;14:4627. doi: 10.1038/s41467-023-40255-4 (PMC10397198; doi:10.1038/s41467-023-40255-4)
Supplement: Supplementary file 4 — Supplementary Data 1 [file 41467_2023_40255_MOESM4_ESM.docx]

**Supplementary Data 1**

**Cartesian Coordinates and Energies of All Investigated Cage Structures**

**2**_2_**R**_2_**S**_2_ (ΔG = 0 kcal/mol)

Total SCF energy (BP86-D3/6-311G(d)/PCM(chloroform)): -5082.883781 a.u.

Thermal correction to the Gibbs free energy at 298.15 K: 1.905980 a.u.

Gibbs free energy at 298.15 K (BP86-D3/6-311G(d)/PCM(chloroform)): -5080.977801 a.u.

O -1.94372300 -3.20243600 3.43678400

O 2.38186400 -3.94361100 -2.37834500

O -0.79496900 2.45983800 -3.89140200

O 0.34038200 4.04731000 3.30953000

N 0.22278700 -6.32534600 1.90627600

N -0.52456700 -6.46498700 -0.85764600

N 1.40265500 -2.01885100 -5.82522900

N 2.31736500 0.71658300 -5.99244700

N 0.13723500 5.97339300 -1.85927200

N -1.67539700 6.21760400 0.40432700

N -1.72285700 1.69404400 5.98870100

N -0.18278300 -0.76709800 6.28656900

C -0.56302400 -3.16739800 3.53839200

C 0.21187600 -4.10188000 2.81485700

C -0.44527800 -5.27043500 2.20334200

H -1.54431500 -5.21615200 2.09125900

C -0.50704700 -7.46178300 1.36412000

H -1.60721200 -7.29237300 1.37721600

C -0.08704100 -7.64112000 -0.12202300

H 1.02074300 -7.74998300 -0.13618300

C 0.37090400 -5.62224200 -1.22648100

H 1.44639800 -5.79091900 -1.03144100

C 0.02634300 -4.42787700 -2.01873700

C 1.05355300 -3.68457600 -2.65213900

C 2.87726800 -3.37148400 -1.12245500

H 3.70946400 -4.02850900 -0.82479900

H 2.09071800 -3.44262900 -0.34798200

C 3.34933400 -1.93074700 -1.30175600

H 4.10597800 -1.90795600 -2.10714500

H 2.50296700 -1.30880400 -1.64796900

C 0.71373000 -2.77993400 -3.68147200

C 1.74453000 -2.29218300 -4.61949100

H 2.79107600 -2.25883800 -4.26587900

C 2.40799500 -1.58501400 -6.77793900

H 3.43520800 -1.59707200 -6.34963900

C 2.07449300 -0.11322300 -7.16032200

H 1.00686000 -0.09711300 -7.47244100

C 1.32603600 1.09156200 -5.26731100

H 0.27490300 0.84519500 -5.50580300

C 1.56530600 1.98133900 -4.11469300

C 0.51716600 2.74655800 -3.55491900

C -1.47333100 1.71176900 -2.82708100

H -1.56621100 2.36497100 -1.93991900

H -0.84333300 0.84532600 -2.54991800

C -2.83022100 1.24805200 -3.33470000

H -2.67096900 0.56269800 -4.18545200

H -3.38494500 2.12169300 -3.72368700

C -3.66489200 0.53766100 -2.24856500

H -4.51384300 0.03629900 -2.74239100

H -3.07082300 -0.28238300 -1.80526600

C -4.19607100 1.46517300 -1.14478000

H -3.39823300 1.85906600 -0.49348900

H -4.91759000 0.94625800 -0.49481300

H -4.72509000 2.33297300 -1.57453300

C 0.82685000 3.83610500 -2.70397500

C -0.18957900 4.86873100 -2.42402400

H -1.21068300 4.67661600 -2.80628600

C -0.85479600 7.02877100 -1.73948500

H -1.84748600 6.72227100 -2.13931200

C -1.03702200 7.35686000 -0.23370500

H -0.02480000 7.55860000 0.18285000

C -1.00492800 5.51406000 1.24222300

H 0.04567700 5.73944600 1.50562400

C -1.65034700 4.40933800 1.97664300

C -0.99192300 3.77268300 3.05101800

C 1.23575500 3.09923400 2.65203000

H 1.18739800 3.26346300 1.55796600

H 0.88313900 2.07042700 2.85650000

C 2.64301400 3.30804100 3.18603400

H 2.93526000 4.36183200 3.02720400

H 2.64003400 3.14020900 4.27822400

C 3.65635900 2.37450500 2.50528200

H 3.65721500 2.55931000 1.41836900

H 3.33513800 1.32377200 2.62101200

C 5.07853200 2.54163800 3.05271200

H 5.14357900 2.21880300 4.10574500

H 5.39793700 3.59717500 3.01481400

H 5.81069000 1.95775500 2.47237500

C -1.70381900 2.88704100 3.89492400

C -1.07167500 2.39116900 5.13074400

H -0.02252900 2.70043300 5.29935200

C -1.04969000 1.32542700 7.22541400

H 0.00711000 1.67536400 7.24485800

C -1.03396500 -0.22025100 7.33539600

H -2.08717800 -0.56646500 7.23537300

C -0.73146700 -1.49962200 5.38658400

H -1.82104200 -1.69282600 5.36645600

C 0.06839300 -2.22757200 4.38416600

C 1.46897600 -2.11629400 4.34229200

H 1.93322000 -1.39542900 5.02066500

C 2.25451600 -2.91759200 3.49106700

C 1.60752500 -3.94532000 2.77878700

H 2.17487000 -4.64856900 2.16354500

C 3.71411200 -2.68683900 3.36177000

C 4.24596800 -1.39052900 3.53177400

H 3.57724200 -0.55571500 3.75840500

C 5.60817500 -1.14110000 3.37556100

H 5.98359300 -0.12123200 3.49240600

C 6.50174500 -2.17474900 3.04190900

C 5.97891000 -3.46694200 2.86878300

H 6.65181400 -4.29231300 2.61177500

C 4.61164100 -3.72173800 3.02947900

H 4.24655800 -4.74626600 2.91175400

C 7.98850500 -1.88493100 2.88268500

H 8.44775200 -1.77285000 3.88160400

H 8.48226300 -2.75168200 2.41104400

C 8.23301800 -0.63222000 2.06390900

C 8.56505800 0.58937600 2.66900300

H 8.71879000 0.63084500 3.75285000

C 8.67712600 1.76351400 1.90788000

H 8.91670800 2.70950000 2.40537300

C 8.46930200 1.74292400 0.52115000

C 8.50839400 3.01859200 -0.31239900

H 9.30886100 2.95236200 -1.06992200

H 8.75252100 3.87701600 0.33562100

C 7.16804800 3.22651300 -0.99192400

C 6.05775300 3.66681700 -0.24990300

H 6.19175400 3.95515400 0.79685900

C 4.78114900 3.68953600 -0.81305700

H 3.93161400 4.00291900 -0.19961600

C 4.56199200 3.26028900 -2.13908900

C 3.18707200 3.15168700 -2.68328200

C 2.87499100 2.17570700 -3.64467500

H 3.65042300 1.52649400 -4.05764600

C 2.15355600 4.01211700 -2.27185700

H 2.36109700 4.87439700 -1.63256200

C 5.68654900 2.87851300 -2.89904200

H 5.56549600 2.59405300 -3.94825700

C 6.96529900 2.86309700 -2.33373400

H 7.81931400 2.54692500 -2.94242100

C 8.15301000 0.51219200 -0.08696800

H 7.96746200 0.47708600 -1.16534100

C 8.03227200 -0.65272800 0.67061300

H 7.75330700 -1.59265100 0.18183200

C -0.47924800 -0.66883500 8.70350800

H -0.51236100 -1.77079600 8.75290800

H 0.58704600 -0.38189000 8.75755700

C -1.25360200 -0.03897100 9.87090400

H -0.81603100 -0.36495500 10.83025800

H -2.29892100 -0.40236400 9.86197600

C -1.24710100 1.49426000 9.76499800

H -1.83118700 1.94422700 10.58628000

H -0.21015000 1.86460500 9.87627500

C -1.81406500 1.95023000 8.41220700

H -1.77617600 3.04888500 8.31486800

H -2.87828200 1.66181300 8.33067900

C -3.04028100 2.57456800 3.58889600

H -3.57073100 1.91534300 4.28143900

C -3.68555000 3.11219800 2.45947400

C -2.97481800 4.04426800 1.68307600

H -3.43665500 4.52117300 0.81550200

C -5.06875600 2.72726300 2.08462900

C -5.58474700 1.45145900 2.39710000

H -4.97899100 0.74340400 2.97006900

C -6.84210000 1.05245400 1.93951000

H -7.20237500 0.04466200 2.16345200

C -7.63424200 1.90683100 1.15310300

C -7.15324700 3.20175100 0.89809000

H -7.76594900 3.90118400 0.31928900

C -5.89661500 3.60630100 1.35585700

H -5.56015800 4.62524900 1.14535000

C -8.90157400 1.39490700 0.49634500

H -9.47941300 0.77442300 1.20162100

H -9.54925800 2.24504700 0.21760900

C -8.56281500 0.57411600 -0.74443500

C -9.01407200 -0.74468000 -0.89926900

H -9.64656300 -1.19471900 -0.12638700

C -8.64943600 -1.50340800 -2.02273500

H -9.00061600 -2.53708700 -2.11370700

C -7.82155200 -0.96407200 -3.01875400

C -7.31443900 -1.80429100 -4.17812300

H -7.42761100 -1.25470300 -5.12797500

H -7.92357500 -2.72092800 -4.26949500

C -5.85283300 -2.17420600 -3.97322800

C -4.85927300 -1.84758100 -4.90875900

H -5.13681000 -1.34376300 -5.84087600

C -3.51534500 -2.16078600 -4.67267900

H -2.78112800 -1.92038900 -5.44615800

C -3.10738700 -2.79248500 -3.47885200

C -1.68131100 -3.11545400 -3.21352100

C -0.63937900 -2.48126900 -3.91601900

H -0.85092500 -1.74153500 -4.69137700

C -1.31642700 -4.12496700 -2.30206200

H -2.07127400 -4.75365300 -1.82313400

C -4.11507100 -3.11721400 -2.54276200

H -3.84960300 -3.58895200 -1.59280000

C -5.45446400 -2.82391600 -2.79169500

H -6.20889600 -3.07366600 -2.04154800

C -7.37988200 0.36454700 -2.86878700

H -6.72415800 0.80249500 -3.62914300

C -7.74381600 1.11898400 -1.75309600

H -7.36435600 2.13974500 -1.64718200

C -1.93064400 8.59906200 -0.04139100

H -2.95116700 8.34715600 -0.38411100

H -2.00506200 8.81626500 1.03808600

C -1.40213100 9.81487100 -0.81647700

H -2.07701900 10.67570500 -0.66962200

H -0.41665100 10.11473400 -0.41174700

C -1.25946500 9.48572500 -2.31088400

H -0.85363800 10.35040300 -2.86384600

H -2.25963400 9.28008300 -2.73778800

C -0.35013900 8.26499700 -2.51485100

H 0.67462500 8.49496400 -2.16970600

H -0.27148600 8.00067900 -3.58342800

C 2.96259900 0.37416600 -8.32062200

H 2.66732200 1.40478300 -8.58281200

H 4.00517800 0.42785700 -7.95681800

C 2.88021100 -0.55384900 -9.54126000

H 1.85259200 -0.53887900 -9.95224100

H 3.54332200 -0.18234300 -10.34146200

C 3.25160700 -1.99421700 -9.15382900

H 3.16829500 -2.66658100 -10.02508900

H 4.31025600 -2.02537400 -8.83238100

C 2.35175000 -2.50142000 -8.01747000

H 2.63349700 -3.52391900 -7.71215600

H 1.30183600 -2.55190500 -8.36001700

C -0.73315900 -8.90425100 -0.72141000

H -1.82782700 -8.75306100 -0.75586900

H -0.39622800 -9.01326300 -1.76662100

C -0.39845600 -10.15848500 0.10071900

H -0.89286400 -11.04059200 -0.34139600

H 0.69024100 -10.35219600 0.05333100

C -0.81949700 -9.98153300 1.56890500

H -0.54440600 -10.87066200 2.16205400

H -1.92143300 -9.89502100 1.62517200

C -0.17468200 -8.72711900 2.17860800

H -0.50782600 -8.57613900 3.21986500

H 0.92447700 -8.84139200 2.21083000

C -2.40193400 -2.30993800 2.37095000

H -1.77831200 -2.48334900 1.47349500

H -2.24841800 -1.26200900 2.69357100

C -3.86237900 -2.59536200 2.07119000

H -4.46255600 -2.43542200 2.98512000

H -3.96843100 -3.66235700 1.80325300

C -4.39016100 -1.70935300 0.93044800

H -4.29474800 -0.64589400 1.20976400

H -3.76029500 -1.83878000 0.03159300

C -5.84745100 -2.01855600 0.57742900

H -5.95426500 -3.05373600 0.21203600

H -6.23526400 -1.34254100 -0.20027000

H -6.50128200 -1.92036100 1.45935500

C 3.94366000 -1.33873400 -0.01127800

H 4.75316100 -1.99212500 0.36499100

H 3.17773700 -1.33974600 0.78522900

C 4.48463900 0.08313600 -0.20968900

H 4.95332600 0.46341400 0.71061900

H 3.68771900 0.78757200 -0.50103500

H 5.25353300 0.12050700 -0.99899700

**2**_2_**S**_4_ (ΔG = 5.4 kcal/mol)

Total SCF energy (BP86-D3/6-311G(d)/PCM(chloroform)): -5082.879144 a.u.

Thermal correction to the Gibbs free energy at 298.15 K: 1.910001 a.u.

Gibbs free energy at 298.15 K (BP86-D3/6-311G(d)/PCM(chloroform)): -5080.969143 a.u.

O -1.12139900 3.09468500 -2.92935400

O 0.50972700 4.06805300 2.70130500

O -0.54251500 -3.24505500 3.11866100

O 0.93192400 -3.86858000 -2.96174900

N 0.54173500 6.29630100 -0.99795700

N -2.29994100 5.84592600 0.28649300

N -0.45532400 1.53257500 5.79901000

N 1.46583200 -0.70874200 5.67937100

N 1.47700900 -6.01631700 0.84198800

N -1.42046200 -6.11715000 -0.45680600

N -0.56053200 -1.44560500 -5.93131200

N 1.08760500 0.99844600 -5.74914300

C 0.25993900 3.20314700 -2.94084100

C 0.88561500 4.20633700 -2.16270100

C 0.04653000 5.26331300 -1.58058700

C -0.14354700 8.60026200 -1.43552000

C -0.36818200 7.35624200 -0.54267600

C -1.46599000 5.33150800 1.11786100

C -1.80313200 4.19513900 1.98562700

C -0.79422600 3.63245500 2.80711100

C 1.24711100 3.60129700 1.52340300

C 2.02123700 2.32360700 1.82747200

C -1.14066900 2.69444100 3.80644300

C -0.13776800 2.27668400 4.80308100

C 0.55293900 1.26810200 6.81494700

C 0.78878200 -0.26152400 6.89000700

C 0.82780800 -1.47017600 4.86648100

C 1.51309800 -2.13823600 3.74223300

C 0.82472200 -3.09858600 2.96617400

C -1.31589400 -2.43722500 2.17771300

C -2.77622500 -2.50291600 2.59941100

C -3.71252100 -1.67885200 1.69507500

C -3.85938800 -2.22202900 0.27039900

C 1.54276800 -3.96245900 2.10520100

C 0.84511900 -5.10964800 1.49716900

C -0.78781300 -7.17515100 0.33880500

C -1.33077600 -8.53292000 -0.17139700

C -0.70684900 -5.44024800 -1.28325200

C -1.28987800 -4.42636600 -2.17529100

C -0.43907000 -3.69313500 -3.03216200

C 1.56507200 -2.89403600 -2.07730900

C 3.05584400 -2.87434400 -2.36346900

C 3.78024500 -1.81421900 -1.52144400

C 5.27470000 -1.72391600 -1.85180100

C -0.97699600 -2.78938200 -3.97614500

C -0.09922900 -2.19986100 -5.00192400

C 0.35316900 -1.00557400 -6.97555600

C 0.41076000 0.54239800 -6.95840200

C 0.39664500 1.62803900 -4.86905000

C 1.03823200 2.34424400 -3.74970000

C 2.43592400 2.35797400 -3.58927400

C 3.07208100 3.23389700 -2.68945400

C 2.28387600 4.19681700 -2.03423600

C 4.53453900 3.15119200 -2.46205200

C 5.17259400 1.89401200 -2.41494100

C 6.54479900 1.79122100 -2.18482400

C 7.33676100 2.93883500 -1.99370300

C 6.70318400 4.19260800 -2.03500500

C 5.32638600 4.30021700 -2.26568200

C 8.83690500 2.81432800 -1.74361900

C 9.14656400 1.67145400 -0.79699800

C 9.60105700 0.43046000 -1.26893800

C 9.71557500 -0.66969200 -0.40538100

C 9.38346600 -0.55305300 0.95248000

C 9.33793300 -1.76814000 1.86701800

C 7.88629900 -2.13765300 2.12649600

C 7.11289400 -2.73339800 1.11441700

C 5.74660800 -2.95724200 1.28938300

C 5.09008800 -2.58385400 2.48179300

C 3.62566200 -2.77139500 2.64685600

C 2.89355300 -1.96930700 3.54212900

C 2.93016900 -3.78497300 1.95965100

C 5.87753200 -2.01877000 3.50738900

C 7.24761900 -1.80022200 3.33101400

C 8.97080500 0.70427200 1.43440500

C 8.85287500 1.79704300 0.57446300

C 1.19532400 1.08325500 -8.17277500

C 0.63926800 0.55874800 -9.50430000

C 0.62969700 -0.97762200 -9.51804400

C -0.17603400 -1.52397800 -8.33076900

C -2.36151300 -2.54600200 -3.96805900

C -3.21962500 -3.19946800 -3.06572100

C -2.67055000 -4.17131800 -2.21027000

C -4.65964100 -2.85517900 -2.99506600

C -5.07991200 -1.51263400 -3.11361500

C -6.42632600 -1.17009700 -2.97814400

C -7.40148300 -2.15151600 -2.72163600

C -6.98698000 -3.48952100 -2.61895600

C -5.63850300 -3.83778000 -2.75259300

C -8.84513900 -1.75026800 -2.46848200

C -8.97696200 -1.12447400 -1.08844400

C -9.09665900 0.26616900 -0.92635500

C -9.11859100 0.83956800 0.35000800

C -9.02453300 0.04015700 1.50201200

C -8.93351400 0.68308300 2.87888200

C -7.54152300 1.25949100 3.07194400

C -6.46710400 0.43071500 3.43984800

C -5.15958300 0.92037400 3.47074500

C -4.87593300 2.25549800 3.11886200

C -3.47833900 2.74313200 3.03743100

C -2.47781700 2.26547600 3.90255400

C -3.11880100 3.72108400 2.09210200

C -5.96130900 3.09704700 2.79985500

C -7.26909800 2.60646100 2.77834300

C -8.91982600 -1.35212000 1.34016200

C -8.89420000 -1.92526800 0.06382100

C -0.85939300 -9.70471500 0.70228800

C 0.67401300 -9.73989300 0.79584300

C 1.22637200 -8.39356500 1.28636300

C 0.76414200 -7.21732300 0.38995800

C 1.67998700 -0.63010000 8.09551000

C 1.12616600 -0.08220400 9.41846300

C 0.94496400 1.44130500 9.33756300

C 0.03076800 1.81472200 8.16203700

C -1.88782700 7.03144600 -0.47702300

C -2.65889000 8.25663200 0.06956900

C -2.42306200 9.50619500 -0.79279700

C -0.92311800 9.81886700 -0.91784200

C -1.55837600 2.18264100 -1.86878100

C -3.05167200 2.33563600 -1.63039100

C -3.54100400 1.34333900 -0.55854300

C -5.05001800 1.42651000 -0.29948100

C 2.92094500 1.88662400 0.65806800

C 3.79523000 0.67720000 1.01321600

H -1.03383300 5.11058400 -1.72769900

H 0.93789400 8.80939300 -1.48387800

H -0.46586500 8.35075900 -2.46436000

H -0.02348700 7.63433900 0.47349800

H -0.44691600 5.72335600 1.26325400

H 1.92222000 4.43021900 1.25894900

H 0.54824400 3.46268800 0.67916100

H 2.64103400 2.49930400 2.72525100

H 1.31363700 1.51547500 2.09039400

H 0.87898400 2.69080100 4.67389800

H 1.52772900 1.74863400 6.57424700

H -0.20923200 -0.73945200 7.00839200

H -0.23090700 -1.75042200 5.02355600

H -1.15725200 -2.83496500 1.15759600

H -0.94121000 -1.39612700 2.20556000

H -2.84417300 -2.14157000 3.64118200

H -3.09569600 -3.56076100 2.60658700

H -3.36924200 -0.62839100 1.66131700

H -4.70875500 -1.64708000 2.16734800

H -2.91991500 -2.16844900 -0.30194000

H -4.61833200 -1.66173100 -0.29665300

H -4.17355100 -3.27914400 0.27818200

H -0.23904200 -5.14761500 1.69406900

H -1.17155500 -7.05065600 1.37122400

H -0.98424400 -8.67274200 -1.21311200

H -2.43143000 -8.47917200 -0.20842300

H 0.38125700 -5.57262200 -1.39701900

H 1.35324500 -3.17379800 -1.02644100

H 1.11736500 -1.89963700 -2.26437200

H 3.47962900 -3.87472900 -2.16303100

H 3.20601100 -2.67195700 -3.43939600

H 3.64292300 -2.03690000 -0.44835600

H 3.30449600 -0.82818700 -1.67900700

H 5.80243400 -1.04807800 -1.16127900

H 5.43485200 -1.35908200 -2.88052500

H 5.76174600 -2.71047000 -1.77602000

H 0.96607900 -2.49641300 -4.95824400

H 1.38750500 -1.38428900 -6.81406400

H -0.63958100 0.90671900 -7.00624100

H -0.69638600 1.77193800 -4.96587400

H 3.02536200 1.69316400 -4.22689800

H 2.74013000 4.95060700 -1.38659000

H 4.57712000 0.98382500 -2.52867300

H 7.00646500 0.80140500 -2.12570600

H 7.29583600 5.10227200 -1.88885400

H 4.86638500 5.29210600 -2.31406800

H 9.21858200 3.76668300 -1.33721800

H 9.35749200 2.65705400 -2.70575900

H 9.84046700 0.31211800 -2.33144600

H 10.04634000 -1.63634500 -0.80044400

H 9.84717400 -1.55192700 2.82142500

H 9.87220400 -2.61216600 1.39920500

H 7.58353000 -2.99455600 0.16077700

H 5.17700900 -3.40482600 0.47167800

H 3.38637800 -1.19651800 4.13686900

H 3.45889400 -4.50178500 1.32547700

H 5.42286500 -1.76070600 4.46781100

H 7.82946700 -1.35399400 4.14461400

H 8.70443900 0.81327700 2.49127000

H 8.49403700 2.75741900 0.96042500

H 2.25334100 0.78254300 -8.06161900

H 1.17665400 2.18617700 -8.13986000

H 1.23967400 0.95320000 -10.34219500

H -0.39174100 0.93315300 -9.65162000

H 0.20591400 -1.35766100 -10.46364500

H 1.67011100 -1.35134800 -9.46607900

H -0.16143600 -2.62735900 -8.31268600

H -1.23590500 -1.22369100 -8.42445100

H -2.75959100 -1.84811200 -4.70998700

H -3.30308500 -4.72391500 -1.51108900

H -4.33640600 -0.72413000 -3.26776400

H -6.72677100 -0.11916300 -3.04457800

H -7.73010100 -4.27162100 -2.42991000

H -5.34429700 -4.88948000 -2.68194200

H -9.49822000 -2.63570900 -2.54887000

H -9.18079300 -1.03382400 -3.23727400

H -9.16540100 0.90922100 -1.81051100

H -9.19357100 1.92733600 0.45322900

H -9.15119500 -0.06529600 3.65896500

H -9.68854300 1.48138500 2.97655700

H -6.65451500 -0.62268900 3.67412900

H -4.34098400 0.24301000 3.72713300

H -2.72190000 1.56685000 4.70754600

H -3.86226300 4.14991300 1.41490600

H -5.78325500 4.15202300 2.57153100

H -8.09251100 3.27969300 2.51743900

H -8.84691100 -1.99477100 2.22429700

H -8.79124300 -3.01077000 -0.04171700

H -1.24653700 -10.65540100 0.29666900

H -1.28688400 -9.59789300 1.71743800

H 1.09796600 -9.96720800 -0.20074400

H 1.00447400 -10.54901300 1.46988800

H 2.32851100 -8.40088600 1.31668600

H 0.88155000 -8.19513300 2.31901500

H 1.14102500 -7.43120300 -0.63003900

H 2.69138200 -0.22230300 7.91367800

H 1.78504900 -1.72812400 8.13146900

H 1.80265900 -0.35186600 10.24782500

H 0.15107800 -0.55643900 9.64026800

H 1.93361000 1.92179100 9.20881400

H 0.52368300 1.83568200 10.27841300

H -0.08062600 2.90927500 8.07506100

H -0.98303300 1.40665400 8.32931500

H -2.25227300 6.86461200 -1.51042700

H -2.32395600 8.44142800 1.10800700

H -3.73123700 8.00492400 0.12206600

H -2.96452200 10.36699600 -0.36385600

H -2.84783700 9.33761500 -1.80063500

H -0.76122700 10.68100000 -1.58765400

H -0.52523400 10.11084700 0.07267500

H -1.29727300 1.14960300 -2.16895800

H -0.99590100 2.42020200 -0.94511400

H -3.59266400 2.16957200 -2.57960100

H -3.25865300 3.37300300 -1.31011000

H -3.27824500 0.31434700 -0.86448500

H -2.99822000 1.53444600 0.38548300

H -5.62703600 1.15892100 -1.19999900

H -5.36099700 0.74568100 0.50875100

H -5.34790600 2.44266500 0.00659100

H 2.30206400 1.66103300 -0.23040300

H 3.56775400 2.73028900 0.35456300

H 4.47264700 0.41755300 0.18568300

H 4.42213200 0.88134400 1.89751600

H 3.18773500 -0.21293700 1.24536500

**2**_2_**S**_2_**R**_2_ (ΔG = 9.1 kcal/mol)

Total SCF energy (BP86-D3/6-311G(d)/PCM(chloroform)): -5082.879611 a.u.

Thermal correction to the Gibbs free energy at 298.15 K: 1.916313 a.u.

Gibbs free energy at 298.15 K (BP86-D3/6-311G(d)/PCM(chloroform)): -5080.963298 a.u.

H 4.26148500 -0.18105700 1.65469400

H 5.02542300 1.02655000 2.71102400

H 5.66243100 0.73209500 1.07308200

H 4.35937700 2.84565900 1.02422200

H 3.49063400 1.63371700 0.08799600

H -4.75401900 0.56962300 -0.26684300

H -4.17847300 -0.33095600 -1.68262900

H -4.63869900 1.37956700 -1.83760200

H -2.68995900 1.91714200 -0.18620800

H -2.18868600 0.23211500 -0.33823100

H -2.46647600 2.20379500 -2.70011500

H -1.86898900 0.53823600 -2.82703400

H -0.55792200 2.76483000 -1.14214000

H 0.09569700 1.11723700 -1.39357200

H 1.41499100 7.10193000 -0.11595900

H 1.63007800 7.61293400 -3.12024900

H 2.89594900 7.92397500 -1.91938800

H 1.60714100 10.03042100 -2.45887900

H 1.43271700 9.58669400 -0.75294400

H -0.61319800 9.03280100 -2.99117800

H -0.80252300 10.19680100 -1.66945000

H -0.43183500 0.41520500 6.95529000

H -0.04663600 3.40819900 7.45583700

H -1.46250900 2.45149900 7.92454500

H 0.13368700 2.74097800 9.86359900

H -0.04720500 1.03300600 9.42905000

H 2.27997600 2.87713300 8.60048100

H 2.37177500 1.54236400 9.76186600

H -0.71946700 -7.02437400 0.49292600

H -0.76199900 -7.26885000 3.54480800

H 0.43494600 -8.00387000 2.46471000

H -1.35732200 -9.66277500 3.11649700

H -1.35070300 -9.37780100 1.36780200

H -3.26966300 -8.08525500 3.40530800

H -3.70212100 -9.31282600 2.20314800

H -9.35029300 -1.83949500 -0.06450100

H -9.01409700 -0.25256900 1.81299900

H -7.27247500 2.82903900 -0.25800400

H -4.84709200 3.19026100 -0.06927500

H -3.18987000 4.61921600 0.48360600

H -3.06198600 2.09353100 4.00848000

H -5.13848000 2.88231400 4.23662900

H -7.56832800 2.51274700 4.03745100

H -9.59191900 3.45722900 1.56587700

H -9.52043200 2.12061900 2.71895200

H -10.03318800 3.03879700 -0.78719800

H -10.35950800 1.45028000 -2.67127900

H -10.27649400 -0.77257600 -3.58206300

H -10.55178500 -2.13324000 -2.47535300

H -6.38770700 -4.34938400 -2.06516600

H -8.78056500 -3.73663500 -2.07349700

H -7.79864900 0.10585700 -3.77642800

H -5.40553700 -0.48983900 -3.75985000

H -4.53526300 -3.92613200 -0.88170900

H -3.56529100 -1.69079900 -4.47464300

H -0.21222600 -0.09914000 -6.73589700

H -0.64305200 -3.08139500 -7.27381700

H -1.69649900 -1.76267300 -7.81045500

H -0.14477900 -2.50821000 -9.65684300

H 0.15708300 -0.81871200 -9.21531800

H 1.78582200 -3.25499900 -8.25668300

H 2.33815700 -2.02444500 -9.40622400

H 10.26971900 0.82130700 0.84755100

H 9.81552700 -1.19425800 2.22436500

H 7.83563400 -2.48251500 3.63899300

H 5.46413000 -2.08870500 4.18346800

H 2.52913300 -4.57253400 1.73168600

H 3.60584700 -0.94473000 3.86278600

H 4.30518100 -3.98945300 0.47254100

H 6.68055500 -4.40310600 -0.05064800

H 9.04477100 -4.58685800 0.82962300

H 9.51532600 -3.71019900 2.29829500

H 8.98448000 -3.45545600 -1.35881400

H 9.42162900 -1.43638800 -2.73358400

H 10.49933000 0.93020700 -2.80985500

H 10.47245500 1.90081600 -1.32310000

H 5.76422800 2.84368400 -0.63190500

H 8.16448600 2.40821000 -0.24048000

H 8.52171600 0.77588400 -4.22120900

H 6.12169000 1.21433200 -4.62072200

H 4.10771800 4.09760700 -1.79161800

H 4.07340400 0.38312600 -4.02905400

H 0.36930300 0.58486500 -4.69552200

H 3.23791000 -1.56968100 -7.09271900

H 2.18449900 -0.25631500 -7.64305800

H 1.40053400 -2.54805900 -5.77672600

H 0.12620400 -2.40291700 -4.10295000

H 4.73352800 -1.83747800 -0.64773300

H 4.36917600 -1.22159000 -2.27847100

H 4.70458500 -0.09262500 -0.95826000

H 2.27011600 -0.08592700 -1.27561500

H 2.56768000 -0.97844000 0.22635700

H 2.16060100 -2.26955000 -2.54611800

H 2.35146700 -3.16199300 -1.01819500

H 0.05634000 -1.21841100 -1.68375400

H 0.19448300 -2.17689900 -0.17370200

H -0.93853500 -4.88367500 -0.30459800

H -4.34570700 -6.99953500 1.41829000

H -3.14609400 -7.73358500 0.34071800

H -2.58238800 -5.72993000 2.57904800

H -1.12029600 -4.24465900 2.66891800

H -4.63023000 -2.54309700 1.92399500

H -5.32549700 -1.29315400 0.86691500

H -3.65778600 -1.82522400 0.62859600

H -4.79602000 -0.12817900 2.95597800

H -3.54755200 0.36025000 1.82687100

H -3.25781100 -1.92006400 3.88818000

H -2.67561100 -0.26587400 4.15626900

H -0.98824100 -0.47702700 2.42229300

H -1.68657200 -2.01448800 1.81878800

H 0.03990600 -0.41380900 5.01768800

H 3.25141400 0.84871400 7.49634400

H 1.83417000 -0.10966400 7.95741800

H 1.83460300 2.33269500 6.11880800

H 0.68135300 2.52083700 4.37968900

H 1.97381600 1.22361600 2.11621100

H 2.89641300 2.43523300 3.01025200

H 1.09711000 2.93477500 0.57456300

H 2.33431000 4.10865300 1.07195300

H 0.44749600 5.51268000 0.79320900

H -2.05008600 8.04931100 -1.19404400

H -0.78534800 8.33919700 0.01348100

H -0.53175700 6.52851900 -2.43755600

H 0.37997000 4.58195000 -2.30966500

C 4.73852700 0.81025400 1.66784800

C 3.80511600 1.89250300 1.11537300

C -4.14662800 0.64404200 -1.17856700

C -2.70300300 1.05291900 -0.87378600

C -1.92802500 1.41043200 -2.15093400

C -0.52897400 1.91042300 -1.84504000

C 1.20702700 6.79470200 -1.16000000

C 1.80345700 7.90029600 -2.06574900

C 1.17474100 9.27311700 -1.78240700

C -0.35387600 9.22331200 -1.93245200

C -0.07819700 1.41067200 6.62016600

C -0.37109400 2.39967100 7.77595200

C 0.34966200 2.00245300 9.07224000

C 1.86378900 1.88053300 8.84215600

C -0.88583900 -6.58210600 1.49578700

C -0.62293500 -7.70076300 2.53538300

C -1.56050100 -8.90094600 2.34408300

C -3.03041000 -8.45734900 2.39074600

C -9.51958100 -0.76903700 -0.22252300

C -9.32627400 0.12371500 0.83264700

C -6.81245000 2.86046700 0.73449600

C -5.43607700 3.06570600 0.84301400

C -2.64391300 4.08245900 1.26437100

C -2.57705600 2.70572100 3.24392800

C -3.32723500 3.28047500 2.20083600

C -4.79315000 3.07238200 2.09902600

C -5.59307400 2.86413000 3.24165800

C -6.97369200 2.66332300 3.12979300

C -7.61033600 2.66364900 1.87655000

C -9.11966400 2.47584300 1.75423000

C -9.48764100 1.50998700 0.64446100

C -9.89045100 1.96534800 -0.62064800

C -10.07697100 1.06834600 -1.68417800

C -9.87464900 -0.30808400 -1.50562600

C -9.88705900 -1.27717200 -2.68196700

C -6.68446400 -3.36265900 -2.43277800

C -8.04089800 -3.02171000 -2.44957000

C -8.46333100 -1.76150600 -2.90397600

C -7.49248000 -0.88295400 -3.41850000

C -6.13839100 -1.22067000 -3.40716100

C -5.70089300 -2.45176900 -2.87030500

C -3.81876300 -3.49331600 -1.58457800

C -4.25464500 -2.73011900 -2.68393300

C -3.27807200 -2.22072000 -3.56214700

C -0.15794600 -1.15663300 -6.40847100

C -0.64638400 -2.02366300 -7.59922800

C 0.23121900 -1.85954000 -8.84678900

C 1.69617200 -2.18446300 -8.52272100

C 9.99179100 -0.11799300 0.35708400

C 9.73740800 -1.25533000 1.13348000

C 7.06229000 -2.81432800 2.93809400

C 5.71822900 -2.57830200 3.23904200

C 2.26728600 -3.65992100 2.27380700

C 2.87073300 -1.65801000 3.48226400

C 3.26341300 -2.75521400 2.68991000

C 4.69215500 -2.99427800 2.36451800

C 5.07154000 -3.66472900 1.18236600

C 6.41686700 -3.89860100 0.88477800

C 7.43628400 -3.47567400 1.75560500

C 8.90208100 -3.64425000 1.38398200

C 9.35007800 -2.46530900 0.53365900

C 9.27192000 -2.51863700 -0.86929100

C 9.52408900 -1.38259900 -1.64436600

C 9.86533000 -0.15893400 -1.04148300

C 9.93859100 1.11092800 -1.87746400

C 6.36609000 2.40490500 -1.43142900

C 7.72304500 2.16006500 -1.21144200

C 8.51790000 1.54938000 -2.19803100

C 7.91870900 1.23164100 -3.42861300

C 6.55848200 1.46930600 -3.65043000

C 5.74936700 2.03875800 -2.64628600

C 3.57769400 3.27649800 -2.28278300

C 4.28460500 2.18719900 -2.82690200

C 3.56199100 1.22279300 -3.55248900

C 2.16405200 1.27511100 -3.67071600

C 1.42316500 0.31791500 -4.51298700

C 2.19546900 -1.32455200 -7.35415000

C 1.32472900 -1.48462300 -6.07930900

C -0.88333600 -2.00969300 -4.31051700

C -1.90480800 -2.40883200 -3.32729600

C 4.21060500 -1.04364500 -1.20235600

C 2.71315700 -1.01402500 -0.86966400

C 1.96902100 -2.22345000 -1.45848300

C 0.46659800 -2.14916100 -1.24691400

C -1.49683700 -3.12558500 -2.17712900

C -2.45623400 -3.71750700 -1.32761800

C -2.00791200 -4.62230800 -0.25599800

C -3.30066600 -7.34647200 1.36596700

C -2.36980800 -6.12365900 1.56467100

C -0.11111700 -4.48389200 2.29383700

C 0.91296800 -3.47255600 2.59973800

C -4.41141000 -1.59780800 1.39885700

C -3.93673400 -0.51318400 2.38002400

C -2.87588800 -1.01913400 3.37408600

C -1.55236500 -1.38273500 2.71752000

C 0.54335700 -2.33413600 3.35027200

C 1.52829600 -1.44211500 3.83193300

C 1.11453800 -0.39660500 4.78005800

C 2.16791200 0.91122300 7.69066100

C 1.45658600 1.32331600 6.37752000

C -0.40268400 2.35616100 4.47190500

C -1.18617100 2.87956000 3.34343200

C 2.57033600 2.14599900 1.99530200

C 1.70935700 3.27267600 1.42810100

C -0.51610500 3.63683900 2.35310300

C -1.25480300 4.27958000 1.32983900

C -0.56061100 5.23055300 0.44858700

C -0.95796200 8.11560200 -1.05659000

C -0.33447200 6.73294200 -1.36676500

C 1.43701000 4.58151500 -1.99615900

C 2.17998600 3.37698800 -2.40037100

C 1.47200600 2.33315200 -3.04242900

N 2.00068800 -0.70240000 -5.03814400

N -1.16772500 -1.31749400 -5.35402600

N -2.81799900 -5.09337700 0.62272200

N 0.16596600 -5.57110900 1.66912000

N 1.94159200 0.42272600 5.32273300

N -0.94233600 1.79035600 5.49179500

N -1.08740400 5.71490100 -0.61803600

N 1.98496000 5.56828500 -1.38249600

O -0.15024400 -3.29343100 -1.91453100

O -0.77799800 -2.13368300 3.70105500

O 0.84075700 3.82109700 2.47310800

O 0.09156600 2.34512200 -3.09462300

**1**_3_**S**_6_ (ΔG = 0 kcal/mol)

Total SCF energy (BP86-D3/6-311G(d)/PCM(chloroform)): -6680.767973 a.u.

Thermal correction to the Gibbs free energy at 298.15 K: 2.251684 a.u.

Gibbs free energy at 298.15 K (BP86-D3/6-311G(d)/PCM(chloroform)): -6678.516289 a.u.

O -12.03569600 -2.25686700 2.10674800

H -12.09682000 -3.16599100 1.61182200

O -11.35369800 3.60303400 0.70918700

H -11.44458900 3.60571600 1.74298900

O -11.63296400 -0.62015400 -3.57186200

H -11.53732700 0.26789300 -4.09273400

N -11.02300600 0.94488900 4.39805000

N -10.78340800 3.46685700 3.19215400

N -10.20726100 3.79082500 -3.17638800

N -10.59905900 1.45079900 -4.68492300

N -11.15159800 -4.25413200 -1.81857800

N -11.37887100 -4.43570700 0.95045400

C -10.72633300 -2.00295900 2.25789100

C -10.32023300 -0.83069700 2.94279400

C -11.31001300 0.13670500 3.44087600

H -12.29234600 0.14985000 2.93009100

C -11.97570400 1.99139400 4.72678200

H -12.84486700 2.00043800 4.03066000

C -11.23313900 3.35504000 4.57039300

H -10.36282700 3.32671400 5.26007100

C -9.55435800 3.18929800 2.89000600

H -8.80898300 2.96454600 3.67530700

C -9.11416700 3.13585800 1.50349400

C -10.06337300 3.34070600 0.45073300

C -9.61890000 3.27904800 -0.89445400

C -10.57953300 3.45147100 -1.99351100

H -11.63999800 3.24727100 -1.74941500

C -11.20989800 3.77414100 -4.22953400

H -12.19953200 3.42602700 -3.85584900

C -10.73566500 2.75614500 -5.31455100

H -9.74801200 3.11349600 -5.67597100

C -9.43730800 1.09223300 -4.23649300

H -8.53978500 1.71183100 -4.41836700

C -9.26553500 -0.12235600 -3.45576400

C -10.39891300 -0.94203500 -3.15074000

C -10.19980100 -2.13253500 -2.40891900

C -11.32990100 -3.00278000 -2.05001200

H -12.32024500 -2.51710300 -1.94967100

C -12.25293700 -5.01263600 -1.25197100

H -13.14287200 -4.37321700 -1.05283900

C -11.75689700 -5.56786300 0.12077400

H -10.87094000 -6.20341800 -0.09206200

C -10.12869200 -4.11785600 1.07534200

H -9.33154500 -4.75659400 0.65052900

C -9.72406500 -2.90043200 1.76222700

C -8.35930000 -2.60166300 1.95206800

H -7.61156100 -3.30346600 1.56539500

C -7.94733100 -1.45238400 2.64891900

C -8.95305800 -0.58559400 3.11992200

H -8.68223100 0.34272200 3.62862500

C -6.52678000 -1.13407900 2.93609000

C -6.19524100 -0.51355400 4.16073800

H -6.97365500 -0.34155400 4.91001900

C -4.88302800 -0.13873000 4.44791500

H -4.66174800 0.34800100 5.40420700

C -3.84100200 -0.36903700 3.53229300

C -4.15950200 -1.02995000 2.33048000

H -3.37374800 -1.23281400 1.59604900

C -5.47701200 -1.40428400 2.03351100

H -5.69289900 -1.86274700 1.06404700

C -2.46443000 0.19212600 3.83656700

H -2.38126300 1.17957300 3.34275100

H -2.41057000 0.41454500 4.91860700

C -1.24702100 -0.62850400 3.45332000

C -0.05573500 0.04012500 3.12500800

H -0.04142900 1.13393500 3.15519000

C -1.22882200 -2.03450500 3.46699900

H -2.13505500 -2.58523700 3.73773900

C -12.85021900 -6.41583400 0.78848900

H -12.47371600 -6.79798900 1.75271700

H -13.71403400 -5.76506400 1.01883900

C -13.28452100 -7.57061500 -0.13001200

H -14.08849200 -8.15242600 0.35224500

H -12.43468300 -8.26580600 -0.26896900

C -13.74552300 -7.05165200 -1.50328600

H -14.66887600 -6.45515200 -1.37643400

H -14.00685200 -7.89494500 -2.16533100

C -12.66523900 -6.18060600 -2.16651600

H -11.76774600 -6.78535500 -2.39278200

H -13.02307500 -5.77090400 -3.12656100

C -8.90904000 -2.45829400 -1.97048200

H -8.81486300 -3.36425100 -1.36856800

C -7.77934000 -1.66331700 -2.24261100

C -7.98885900 -0.49350100 -2.99521500

H -7.14200400 0.15061100 -3.25478500

C -6.43198200 -2.06752600 -1.77288700

C -6.12325200 -3.42713100 -1.53697600

H -6.87998100 -4.19713700 -1.71269800

C -4.84518900 -3.82476100 -1.13354400

H -4.63979600 -4.88890400 -0.97241900

C -3.81496800 -2.88387300 -0.96457200

C -4.12156600 -1.53322700 -1.19181100

H -3.34507900 -0.78022900 -1.05736000

C -5.40124100 -1.12470900 -1.57221900

H -5.59633500 -0.05394500 -1.68213800

C -2.42199600 -3.30730600 -0.52630900

H -2.25923400 -4.36090900 -0.81739300

H -2.36588500 -3.29386100 0.57871700

C -1.30598100 -2.44598900 -1.08805200

C -1.12084800 -2.31380900 -2.47639400

H -1.79550500 -2.83685000 -3.16247600

C -0.44026700 -1.74390200 -0.23451000

H -0.58647400 -1.80829800 0.84488500

C -11.72669500 2.71164300 -6.48679600

H -12.69601400 2.32835900 -6.11734400

H -11.36526000 1.98914800 -7.23842300

C -11.91011800 4.10684100 -7.10702900

H -10.95470600 4.43427800 -7.55968400

H -12.64512400 4.05984700 -7.92864200

C -12.34932600 5.13450100 -6.04964300

H -13.36052500 4.87270100 -5.68429900

H -12.42980300 6.13927000 -6.49887300

C -11.37292400 5.16927200 -4.86210100

H -11.72076000 5.87199800 -4.08572300

H -10.37907300 5.52474100 -5.19125900

C -8.26940700 3.00648900 -1.15772700

H -7.98660600 2.92917100 -2.20997100

C -7.31211400 2.80164000 -0.14255700

C -7.76834800 2.87076400 1.18750800

H -7.06552200 2.73276500 2.01592100

C -5.89284600 2.52486400 -0.48351800

C -5.35351000 2.94510900 -1.71997500

H -5.96462900 3.53195900 -2.41150600

C -4.03926800 2.63659100 -2.08478900

H -3.66466100 2.95715700 -3.06301800

C -3.19257700 1.92334100 -1.22072000

C -3.70943100 1.54800500 0.03337200

H -3.08283500 0.98090700 0.72840800

C -5.03280000 1.82083500 0.38991600

H -5.40847300 1.42155100 1.33683400

C -1.78679200 1.52949000 -1.64855400

H -1.62267900 0.46273800 -1.42102300

H -1.70003300 1.62479900 -2.74309700

C -0.69186100 2.33868000 -0.97778300

C -0.11291200 3.45367900 -1.60866300

H -0.44792900 3.73786900 -2.61228000

C -0.23637700 1.99252600 0.30533200

H -0.64949500 1.11156900 0.80398400

C -12.15553700 4.52376100 4.95048600

H -11.60285900 5.47192300 4.83631000

H -12.99767900 4.55792900 4.23459400

C -12.68559200 4.36370400 6.38508900

H -11.84077000 4.43022600 7.09687300

H -13.36646500 5.19690100 6.62933400

C -13.39934100 3.01326000 6.56867600

H -14.31249400 2.99528400 5.94390500

H -13.73294800 2.89250400 7.61362000

C -12.48808200 1.83988000 6.17178700

H -11.61645700 1.78338100 6.84968700

H -13.02175100 0.87802600 6.25916900

O 11.87343900 -0.33627400 3.09339300

H 11.98909500 0.61596400 3.48009300

O 11.27552900 -3.39458300 -2.15994400

H 11.28771500 -4.16844200 -1.46670300

O 11.87775400 2.69044300 -1.62223400

H 11.84127500 2.43378400 -2.62136900

N 10.63325900 -4.08244300 2.16946100

N 10.52549500 -5.11064800 -0.43130700

N 10.45173700 -0.45244100 -4.85046700

N 10.97761000 2.19143400 -3.98055200

N 11.29316300 4.12521500 2.14816600

N 11.34275900 2.00217100 3.99987300

C 10.55161800 -0.51728700 2.93048500

C 10.07325000 -1.76018500 2.44167500

C 11.00996400 -2.85408900 2.13924900

H 12.04603700 -2.56137000 1.87999900

C 11.58276300 -5.10410000 1.75679300

H 12.49614300 -4.66314300 1.29729900

C 10.88799100 -5.98350800 0.67281400

H 9.97720200 -6.41285900 1.14193500

C 9.31173600 -4.66698700 -0.52421700

H 8.51256600 -5.04108200 0.14193000

C 8.96621700 -3.62034600 -1.47395300

C 9.99921700 -2.98896900 -2.24031500

C 9.66083800 -1.91139200 -3.09972500

C 10.71498100 -1.21715400 -3.85191100

H 11.74908400 -1.36691100 -3.48634700

C 11.55448800 0.29804800 -5.43054900

H 12.49803100 0.16139900 -4.85489400

C 11.17993500 1.80685400 -5.37087800

H 10.23454800 1.92490300 -5.94210600

C 9.77197300 2.33035500 -3.52952300

H 8.89570900 2.19645500 -4.19084000

C 9.51558500 2.68882100 -2.14201600

C 10.60572600 2.88182400 -1.23414200

C 10.33499800 3.31114900 0.09176900

C 11.43107100 3.45404100 1.06014400

H 12.36855600 2.91742100 0.81737500

C 12.36325200 4.04725200 3.12826600

H 13.21902300 3.43443400 2.76360200

C 11.78511200 3.33208600 4.39030100

H 10.92256000 3.94001600 4.73726800

C 10.08363400 1.78533700 3.78359900

H 9.32820400 2.57107600 3.97054700

C 9.61115700 0.50830500 3.27100800

C 8.23134900 0.27368100 3.10091600

H 7.52297100 1.06914700 3.35701200

C 7.75317000 -0.94312600 2.59490100

C 8.69175900 -1.93951500 2.27166400

H 8.34739500 -2.90627200 1.89358500

C 6.30339000 -1.24868800 2.44572700

C 5.75590800 -1.56036400 1.18770100

H 6.37106100 -1.45063400 0.28983500

C 4.43460400 -2.01315400 1.07619700

H 4.03346200 -2.24716800 0.08515600

C 3.63254500 -2.19059600 2.21648000

C 4.16089500 -1.81692800 3.46690700

H 3.54206700 -1.92212500 4.36450300

C 5.47275000 -1.34554100 3.58049000

H 5.88269600 -1.10118700 4.56590700

C 2.26286600 -2.83308700 2.11389300

H 2.02807700 -3.00904100 1.04678600

H 2.30280000 -3.83728500 2.57537500

C 1.10130800 -2.05985900 2.71245200

C 1.09863900 -0.65593100 2.75488400

H 1.99907200 -0.10347100 2.46895900

C -0.06892100 -2.73523500 3.11026500

H -0.08682700 -3.83062200 3.09535900

C 12.84281100 3.25617800 5.50318700

H 12.40447500 2.75463500 6.38291500

H 13.67657800 2.61827400 5.15574300

C 13.36376100 4.65576500 5.86897300

H 14.13883300 4.57580300 6.65027900

H 12.53903600 5.25114300 6.30509800

C 13.91744700 5.38449900 4.63250700

H 14.81554600 4.85147800 4.26657700

H 14.24607900 6.40339600 4.90023800

C 12.87058600 5.45051300 3.50827800

H 12.00566100 6.06226500 3.82445300

H 13.28810700 5.93236700 2.60764600

C 9.00545100 3.55065700 0.47703200

H 8.82929700 3.87785100 1.50582300

C 7.92071600 3.35164600 -0.39408600

C 8.19890300 2.91221900 -1.69914600

H 7.37469200 2.78565900 -2.41020000

C 6.52727100 3.64297300 0.02599800

C 6.19759300 4.89060300 0.59403800

H 6.98545900 5.63429900 0.75219200

C 4.87168200 5.20407800 0.90401700

H 4.63526700 6.18947200 1.32127300

C 3.83016500 4.28704700 0.66527800

C 4.16371200 3.02954600 0.13362700

H 3.37870900 2.28739400 -0.04427200

C 5.49187400 2.71158200 -0.17689000

H 5.73146900 1.71736700 -0.56245500

C 2.39804700 4.67589500 1.01868700

H 2.26057700 5.74844000 0.79501600

H 2.26927700 4.58296300 2.11325100

C 1.32197400 3.87184900 0.32159200

C 0.88072400 4.20772400 -0.97038700

H 1.31883800 5.07218400 -1.48126000

C 0.75517800 2.74569600 0.94128200

H 1.10937500 2.45623800 1.93613200

C 12.27886800 2.66685000 -6.01718100

H 13.20480500 2.56471600 -5.42131200

H 11.98308500 3.72859300 -5.96493300

C 12.53373000 2.23630800 -7.47113800

H 11.62922900 2.43721500 -8.07633800

H 13.34346600 2.84616200 -7.90698700

C 12.88017100 0.73998000 -7.55624100

H 13.84891900 0.56048700 -7.05245000

H 13.01311800 0.43393900 -8.60814500

C 11.79406600 -0.12343800 -6.89354900

H 12.07198800 -1.19116200 -6.91227700

H 10.84051900 -0.03308800 -7.44589500

C 8.32520600 -1.49049100 -3.17591000

H 8.10149200 -0.66164400 -3.85385100

C 7.29617900 -2.08849000 -2.42403300

C 7.64117700 -3.15874200 -1.57983600

H 6.87387400 -3.62344900 -0.95077100

C 5.91145300 -1.56052500 -2.48818300

C 5.68257200 -0.16864800 -2.52150500

H 6.53783400 0.51285800 -2.47659800

C 4.38580400 0.34894200 -2.56333600

H 4.23888700 1.43445100 -2.56819300

C 3.26471900 -0.50048000 -2.57591500

C 3.48867400 -1.88810500 -2.54431900

H 2.63356000 -2.57105800 -2.54908000

C 4.78767100 -2.41039200 -2.50090200

H 4.93176500 -3.49562100 -2.49113400

C 1.86405700 0.09292300 -2.66149000

H 1.84207800 1.05221400 -2.11257400

H 1.65196900 0.35000200 -3.71584100

C 0.76862700 -0.80348900 -2.12917100

C -0.10402400 -1.49733800 -2.98705500

H 0.01241300 -1.39248300 -4.07132100

C 0.59918800 -0.95885400 -0.74448500

H 1.26477500 -0.43109800 -0.05475800

C 11.81689400 -7.11757800 0.21454200

H 11.29986000 -7.72321900 -0.54924600

H 12.70311900 -6.67434900 -0.27651000

C 12.25059100 -7.98676600 1.40684200

H 11.36459400 -8.50023100 1.82653500

H 12.93811800 -8.77903800 1.06469900

C 12.91307600 -7.13707100 2.50520500

H 13.86397000 -6.72170900 2.12081900

H 13.17473600 -7.76589600 3.37350400

C 11.99889100 -5.98371900 2.95212900

H 11.08556400 -6.37972500 3.43303100

H 12.50245200 -5.34803100 3.70043600

**1**_3_**R**_6_(*anti*) (ΔG = 37.4 kcal/mol)

Total SCF energy (BP86-D3/6-311G(d)/PCM(chloroform)): -6680.710831 a.u.

Thermal correction to the Gibbs free energy at 298.15 K: 2.254134 a.u.

Gibbs free energy at 298.15 K (BP86-D3/6-311G(d)/PCM(chloroform)): -6678.456696 a.u.

O -11.50134200 0.02720900 -3.41978600

O -11.62508600 -3.43686500 1.64476400

O -11.79474900 2.74592600 1.99645900

N -9.88481900 -3.75177000 -3.46459500

N -11.17743800 -4.28580400 -0.74412000

N -10.35007500 -1.29732400 4.92301700

N -11.56906400 1.28966500 4.08001600

N -10.07836300 4.63985100 -1.21619400

N -11.20568100 2.54434400 -3.10400600

C -10.16421100 -0.08446900 -3.41550800

C -9.56120400 -1.36813500 -3.51011700

C -10.40120300 -2.57238000 -3.44536400

C -11.73554300 -4.76420700 -2.00916400

C -12.47772200 -6.09785500 -1.77576400

C -9.92038400 -4.01785300 -0.58652600

C -9.43354400 -3.38732200 0.63335300

C -10.32998300 -3.08332100 1.70601300

C -9.83954800 -2.36974800 2.83208500

C -10.76996000 -1.90742400 3.86975100

C -12.24409800 0.36785700 4.99226700

C -13.14755100 1.15026200 5.96774200

C -10.28092600 1.41959000 4.02638700

C -9.65553300 2.23795000 2.99506000

C -10.45672700 2.84846000 1.97676800

C -9.81388400 3.54629400 0.91832700

C -10.61333700 4.04224100 -0.20851400

C -11.85850400 3.82755000 -2.84954800

C -12.63309100 4.26753600 -4.11159700

C -9.91995000 2.38969600 -3.09644300

C -9.32718500 1.07135100 -3.28650700

C -7.92932300 0.92282400 -3.30122900

C -7.31990600 -0.33552700 -3.45380100

C -8.15911400 -1.46130800 -3.54249600

C -5.84481300 -0.47855700 -3.53730500

C -5.27107900 -1.34998500 -4.48624000

C -3.88486200 -1.47646900 -4.60065200

C -3.01144600 -0.75228100 -3.76863800

C -3.58251500 0.11169700 -2.81557900

C -4.97212400 0.25146800 -2.70388100

C -1.51533000 -0.98697700 -3.88436700

C -0.57545600 0.18405600 -3.64516600

C 0.74331200 -0.05988400 -3.22121800

C -0.94004000 1.51690800 -3.90965900

C -13.46983900 5.52854300 -3.84030300

C -12.59737500 6.67444900 -3.30071600

C -11.80413500 6.23305600 -2.06072900

C -10.94361300 4.98360400 -2.35566200

C -8.41459600 3.66923600 0.93242300

C -7.61104000 3.11015500 1.94308500

C -8.25679800 2.37492500 2.95431600

C -6.14313600 3.32867000 1.96459900

C -5.61297800 4.58471200 1.60138500

C -4.24064700 4.83944300 1.67865100

C -3.34572000 3.85786900 2.13790500

C -3.86866400 2.59567300 2.47477700

C -5.23857500 2.32736500 2.38149300

C -1.87130300 4.18856300 2.32361400

C -0.90520800 3.10188500 1.89211000

C -0.17143600 2.36183300 2.83568300

C -0.71520500 2.80784700 0.52966000

C -14.00930200 0.19323400 6.80901800

C -13.14414800 -0.83981500 7.55199700

C -12.22248600 -1.59965200 6.58365100

C -11.33678800 -0.62350700 5.77873700

C -8.49167800 -1.96779200 2.85410300

C -7.59876600 -2.24258900 1.80382900

C -8.09510700 -2.96817400 0.70520700

C -6.20155100 -1.74604500 1.82056200

C -5.45516500 -1.68700100 3.01559500

C -4.12729300 -1.24071800 3.01582700

C -3.49801800 -0.84393100 1.82475900

C -4.24788600 -0.88403500 0.63683300

C -5.57312900 -1.31789500 0.62964400

C -2.04704800 -0.39975900 1.79746000

C -1.12647100 -1.45914400 1.22231500

C -0.49897000 -2.39845900 2.05974600

C -0.89211300 -1.54204600 -0.16022400

C -13.23386400 -6.54510300 -3.03753600

C -12.29197400 -6.64638800 -4.24910600

C -11.52450000 -5.33341900 -4.46951700

C -10.74538400 -4.91387400 -3.20205900

O 12.00194100 1.20580600 -2.79955500

O 11.48171900 2.04475400 3.31731300

O 11.34274100 -3.52489500 0.74015400

N 10.68455800 4.78284600 -1.22646700

N 11.47948900 3.92898700 1.58336300

N 9.66777200 -1.22762600 4.98767000

N 10.95487700 -3.26507600 3.26099200

N 9.88753400 -3.67419800 -3.09300000

N 11.45895600 -1.11882200 -3.73676000

C 10.68792400 1.47927100 -2.84972700

C 10.20152200 2.71112900 -2.33583500

C 11.11124300 3.64878000 -1.66272400

C 12.28763500 4.83070500 0.76353600

C 13.04528100 5.82651900 1.66721900

C 10.20649700 3.75889900 1.41282000

C 9.49420800 2.69761800 2.11285300

C 10.19098300 1.83082400 3.01317800

C 9.50895700 0.71221900 3.56241500

C 10.24897900 -0.24367000 4.39483400

C 11.51884900 -2.88326900 4.55642400

C 12.27333200 -4.07014700 5.18928400

C 9.67760300 -3.32758200 3.05644500

C 9.13987500 -3.54777900 1.72123900

C 10.01064100 -3.58554200 0.58621500

C 9.44537300 -3.66537600 -0.71640600

C 10.33330900 -3.62005500 -1.88619800

C 11.94014500 -2.46520000 -4.04665400

C 12.81915200 -2.41189600 -5.31655400

C 10.20632200 -0.79564600 -3.78742500

C 9.75748100 0.53179300 -3.38837500

C 8.38811300 0.84905500 -3.43426000

C 7.89760600 2.06625200 -2.93459400

C 8.82001100 2.96969500 -2.38265600

C 6.44841200 2.38961400 -2.95432700

C 5.78667500 2.78574800 -1.77721700

C 4.43233200 3.13552400 -1.80128000

C 3.69927700 3.10852300 -2.99787500

C 4.35392000 2.68726500 -4.17126500

C 5.70473700 2.32900400 -4.15137400

C 2.23218000 3.47926500 -3.02692100

C 1.28739500 2.31545900 -3.29399100

C 1.66233800 0.98257500 -3.05557600

C -0.02634300 2.56196800 -3.73048500

C 13.48259000 -3.77001700 -5.59614500

C 12.43534700 -4.89122700 -5.70021100

C 11.54028200 -4.93201800 -4.45211400

C 10.85570800 -3.56908100 -4.19758900

C 8.04843700 -3.75309000 -0.85124300

C 7.17744200 -3.74766600 0.25426000

C 7.75093400 -3.62334600 1.53293900

C 5.71079700 -3.89918900 0.09360000

C 5.01330100 -3.32107900 -0.98852500

C 3.63886300 -3.52703000 -1.14933900

C 2.91099800 -4.31816100 -0.24147600

C 3.59770500 -4.86487400 0.85595400

C 4.97033300 -4.66122300 1.02155600

C 1.42782800 -4.58300100 -0.43813900

C 0.54094800 -3.49618400 0.14706300

C 0.32795600 -3.39770500 1.53349300

C -0.07701300 -2.55044100 -0.68479600

C 13.02025900 -3.63068600 6.46038000

C 12.07173900 -2.96282100 7.47136400

C 11.29883700 -1.79718200 6.83156400

C 10.52184400 -2.26843000 5.58320100

C 8.17404700 0.47227900 3.18851600

C 7.47504300 1.30347700 2.29405500

C 8.15696000 2.42138700 1.77967000

C 6.08551400 0.99395800 1.87164800

C 5.68041300 -0.32938900 1.59076000

C 4.36422300 -0.61466900 1.21150100

C 3.40524100 0.40821900 1.08341000

C 3.81304100 1.72745800 1.34846600

C 5.12543000 2.01673200 1.73571400

C 1.98215600 0.07662300 0.65167200

C 0.95077200 1.10458400 1.06842100

C 0.73925400 1.37946600 2.43070400

C 0.20009600 1.82735900 0.12450300

C 14.05066200 6.65996200 0.85532800

C 13.36434100 7.38712800 -0.31348300

C 12.57816200 6.40610300 -1.19762700

C 11.55032100 5.60199900 -0.36957300

H -11.70275100 1.04145600 -3.30446500

H -11.76480400 -3.84419700 0.70279000

H -12.02270900 2.15737800 2.82499600

H -11.47957000 -2.38619100 -3.29383400

H -12.50332700 -4.01263600 -2.28268200

H -11.73501500 -6.86391000 -1.48315700

H -13.16640800 -5.98026800 -0.92235000

H -9.17915400 -4.17225700 -1.38836900

H -11.84197300 -2.04324400 3.63498400

H -12.91134200 -0.23208300 4.34094200

H -12.50293900 1.76440900 6.62447700

H -13.77782300 1.85084200 5.39458300

H -9.60487800 0.86853800 4.70037200

H -11.69235400 3.81311200 -0.14900400

H -12.61240400 3.61756000 -2.06559100

H -11.90153600 4.46003300 -4.91902500

H -13.27010100 3.43386900 -4.45161800

H -9.22602600 3.22272300 -2.89493200

H -7.30686200 1.82039200 -3.21583300

H -7.72754700 -2.46491600 -3.60128000

H -5.92160800 -1.90897700 -5.16618000

H -3.46995800 -2.15045100 -5.35827400

H -2.93288000 0.69243000 -2.15256900

H -5.38409800 0.91450600 -1.93640800

H -1.30604600 -1.39768300 -4.88966800

H -1.24495100 -1.80410000 -3.19065100

H 1.06654000 -1.08572200 -3.01896500

H -1.95091300 1.74248600 -4.26190700

H -13.98951800 5.83920900 -4.76276500

H -14.25770500 5.28867500 -3.10117400

H -11.89038000 6.99885600 -4.08777600

H -13.21987900 7.55276000 -3.05796300

H -11.14369800 7.04036800 -1.70182800

H -12.49630100 5.99636100 -1.23046000

H -10.26202300 5.24521900 -3.18878700

H -7.95447700 4.20042800 0.09410500

H -7.66446900 1.91261300 3.75163200

H -6.28979000 5.38586000 1.28799100

H -3.86166100 5.82998400 1.40322600

H -3.19244200 1.80687500 2.81391200

H -5.59985000 1.31964000 2.61436100

H -1.64428500 5.11807100 1.77223400

H -1.69526600 4.42392600 3.38959400

H -0.31324700 2.56161000 3.90367300

H -1.28927200 3.35562200 -0.22601300

H -14.62130800 0.76922100 7.52397200

H -14.71805600 -0.33472400 6.14295100

H -12.52532600 -0.32173400 8.30916000

H -13.78239100 -1.55126200 8.10361900

H -11.57057400 -2.30474000 7.12649500

H -12.82669100 -2.20232000 5.87974300

H -10.75266600 -0.03198500 6.51094800

H -8.17013400 -1.36500800 3.70821100

H -7.43164700 -3.20969300 -0.13211400

H -5.90785800 -2.02520500 3.95314300

H -3.56707200 -1.21382400 3.95692800

H -3.78753800 -0.56820800 -0.30222800

H -6.13317900 -1.29881200 -0.31129100

H -1.95456200 0.52022200 1.19801500

H -1.71373700 -0.13961900 2.81425700

H -0.66156800 -2.34368900 3.14167800

H -1.33318500 -0.79939100 -0.83266600

H -14.03559800 -5.81454500 -3.25714400

H -13.73264700 -7.51199100 -2.85362400

H -12.85897500 -6.90956400 -5.15858400

H -11.56755000 -7.46605400 -4.08262100

H -10.81302100 -5.42417800 -5.30747800

H -12.22619200 -4.51986200 -4.73488700

H -10.05656900 -5.74428700 -2.94964100

H 12.10652400 0.24011700 -3.16348500

H 11.78936100 2.82999500 2.71022400

H 11.50921500 -3.42675200 1.76090400

H 12.13758800 3.27676800 -1.49044200

H 13.05063400 4.17470300 0.29711600

H 12.30509300 6.48801000 2.15565800

H 13.55277600 5.26793300 2.47152500

H 9.62793300 4.33730600 0.67325700

H 11.34471300 -0.09591600 4.40767800

H 12.27289600 -2.10719000 4.31604500

H 11.53897700 -4.86209400 5.42947900

H 12.96966100 -4.49533000 4.44701700

H 8.94241000 -3.14850000 3.85706200

H 11.40483100 -3.50017800 -1.64750200

H 12.60735700 -2.73633600 -3.20544000

H 13.57486900 -1.61759000 -5.19665100

H 12.17889800 -2.11681000 -6.16932900

H 9.42101200 -1.51616700 -4.07065500

H 7.68682400 0.10501900 -3.82817500

H 8.47297700 3.92037400 -1.96742500

H 6.32793700 2.78107000 -0.82654700

H 3.93515500 3.41903000 -0.86911900

H 3.79356100 2.64177200 -5.11140600

H 6.20440000 2.02850600 -5.07816700

H 2.06024200 4.26006400 -3.79001000

H 1.95114200 3.93852300 -2.06110700

H 2.67929800 0.75942400 -2.71995700

H -0.34186600 3.59163700 -3.93319100

H 14.19008500 -4.00375600 -4.77798600

H 14.08113000 -3.71155000 -6.52128700

H 11.80544800 -4.72314300 -6.59435100

H 12.92757300 -5.86854100 -5.84327200

H 10.76060400 -5.70661300 -4.54540400

H 12.14102300 -5.19251100 -3.55988900

H 10.26205300 -3.33041400 -5.10175400

H 7.65125700 -3.84617900 -1.86645700

H 7.10411600 -3.56026000 2.41490400

H 5.54997000 -2.68571700 -1.69940500

H 3.12079400 -3.06671600 -1.99772800

H 3.05691100 -5.49072400 1.57391700

H 5.48487800 -5.14550200 1.85724400

H 1.21406700 -4.68451000 -1.51542800

H 1.17093300 -5.55187800 0.02362500

H 0.81100200 -4.11330300 2.20756100

H 0.08902900 -2.61028400 -1.76438400

H 13.52326600 -4.49891200 6.91936800

H 13.81838000 -2.91679000 6.18134700

H 11.34989800 -3.71310500 7.84645100

H 12.63578900 -2.60559100 8.35001000

H 10.58610800 -1.35314200 7.54687300

H 12.00040200 -0.99305500 6.54059500

H 9.83799600 -3.07511300 5.91257500

H 7.68781800 -0.41161900 3.61187800

H 7.66236700 3.07069300 1.04900600

H 6.41139300 -1.14294300 1.64438400

H 4.08019800 -1.65063100 0.99828400

H 3.08297600 2.53912200 1.27907200

H 5.40123200 3.05106800 1.96690000

H 1.95102900 -0.04677800 -0.44493800

H 1.70328700 -0.91088100 1.06051100

H 1.30939900 0.82319400 3.18232500

H 0.33184900 1.62036800 -0.94061700

H 14.83748300 5.99064500 0.45836300

H 14.55870900 7.38352900 1.51546900

H 14.10883200 7.92986700 -0.92094400

H 12.66988700 8.14992100 0.08684100

H 12.04507800 6.93677100 -2.00427200

H 13.26895300 5.69370300 -1.68740200

H 10.87759200 6.33384700 0.12029400
